# Dual PI3K/ERK inhibition induces necroptotic cell death of Hodgkin Lymphoma cells through IER3 downregulation

**DOI:** 10.1038/srep35745

**Published:** 2016-10-21

**Authors:** Silvia Laura Locatelli, Giuseppa Careddu, Giuliano Giuseppe Stirparo, Luca Castagna, Armando Santoro, Carmelo Carlo-Stella

**Affiliations:** 1 Humanitas Cancer Center, Humanitas Clinical and Research Center, Rozzano, Italy; 2 Humanitas University, Rozzano, Italy; 3 Department of Medical Biotechnology and Translational Medicine, University of Milan, Milan, Italy

## Abstract

PI3K/AKT and RAF/MEK/ERK pathways are constitutively activated in Hodgkin lymphoma (HL) patients, thus representing attractive therapeutic targets. Here we report that the PI3K/ERK dual inhibitor AEZS-136 induced significant cell proliferation inhibition in L-540, SUP-HD1, KM-H2 and L-428 HL cell lines, but a significant increase in necroptotic cell death was observed only in two out of four cell lines (L-540 and SUP-HD1). In these cells, AEZS-136-induced necroptosis was associated with mitochondrial dysfunction and reactive oxygen species (ROS) production. JNK was activated by AEZS-136, and AEZS-136-induced necroptosis was blocked by the necroptosis inhibitor necrostatin-1 or the JNK inhibitor SP600125, suggesting that JNK activation is required to trigger necroptosis following dual PI3K/ERK inhibition. Gene expression analysis indicated that the effects of AEZS-136 were associated with the modulation of cell cycle and cell death pathways. In the cell death-resistant cell lines, AEZS-136 induced the expression of immediate early response 3 (IER3) both *in vitro* and *in vivo*. Silencing of *IER3* restored sensitivity to AEZS-136-induced necroptosis. Furthermore, xenograft studies demonstrated a 70% inhibition of tumor growth and a 10-fold increase in tumor necrosis in AEZS-136-treated animals. Together, these data suggest that dual PI3K/ERK inhibition might be an effective approach for improving therapeutic outcomes in HL.

Approximately 9,300 new cases of Hodgkin lymphoma (HL) and 1,200 resulting deaths are estimated to occur each year in the United States^1^. Combination chemotherapy with or without radiotherapy cures approximately 80% of advanced-stage HL cases^2^. However, 20–30% of patients are initially refractory to chemotherapy or experience early or late disease relapse and are not cured using modern treatments^3^. Second-line high-dose salvage chemotherapy (HDC) and autologous stem cell transplantation have established roles in the management of refractory/relapsed HL and lead to long-term complete remission in approximately 50% of relapsed patients and a minority of refractory patients^4^. Refractory/resistant HL patients represent an unmet medical need requiring the development of effective salvage regimens^5^.

Several molecularly targeted agents, including histone deacetylase (HDAC) inhibitors^6^, mammalian target of rapamycin (mTOR) inhibitors^7^, and immunomodulatory drugs^8^, have been tested in phase I/II trials. Used as single agents, these molecules have a limited efficacy^9^. More recently, the alkylating agent bendamustine^10^, the anti-CD30 antibody-drug conjugate brentuximab vedotin^11^,^12^, and the anti-programmed cell death protein-1 (PD-1) antibody nivolumab^13^,^14^ have demonstrated extraordinary efficacy. However, limited evidence has been provided for long-term disease control using these agents, suggesting that either combination therapy or a single agent with multitargeting capacity is required^15^.

Aberrant regulation of the phosphatidylinositol 3-kinase (PI3K)/AKT pathway has frequently been observed in Hodgkin Reed-Sternberg (HRS) cells,^16^,^17^ suggesting that PI3K is an attractive therapeutic target^18^,^19^,^20^. Cancer cells frequently exhibit increased oxidative stress and are likely to be more sensitive to the damage promoted by reactive oxygen species (ROS)^21^. We recently demonstrated that upon HDAC and MEK/ERK inhibition, ROS production is critically involved in lymphoma cell death via necroptosis^22^. Additionally, several studies have implicated MAPKs, PI3K/AKT, and NF-kB in the regulation of cell death^23^.

To investigate the therapeutic potential of PI3K and ERK dual inhibition, we used AEZS-136 [kindly provided by Æterna Zentaris (Frankfurt, Germany, EU)] in preclinical models of HL. AEZS-136 concurrently inhibits Erk1/2 and Pl3K by an ATP competitive mode of action. AEZS-136 is a dual Pl3K/Erk inhibitor based on a pyridopyrazine scaffold. The anti-proliferative efficacy of AEZS-136 was evaluated in more than 40 human tumor cell lines and physio-chemical as well as *in-vitro* ADMET properties were widely assessed. Furthermore, the *in vivo* pharmacokinetics and anti-tumor efficacy was explored. AEZS-136 was well tolerated and showed dose dependent inhibition of human colon tumor growth of up to 72% in a Hct116 mouse model (I. Seipelt, Aeterna Zentaris, personal communication)^24^.

We report herein that AEZS-136 potently induced the dephosphorylation of MAPK and PI3K/AKT pathway components, leading to caspase-independent necroptosis. Besides downregulating the phosphorylated form of the anti-apoptotic proteins Mcl-1 and ERK1/2, AEZS-136 strongly increased JNK expression. These activities were dependent on potent, early, and time-dependent ROS generation and translated into significant antitumor activity *in vivo*. Immediate early response 3 (IER3) was identified as a key signaling molecule that mediated resistance to AEZS-136-induced oxidative death.

## Results

### AEZS-136 modulates the phosphorylation of MAPK and PI3K/AKT pathway constituents

**Figure 1:**
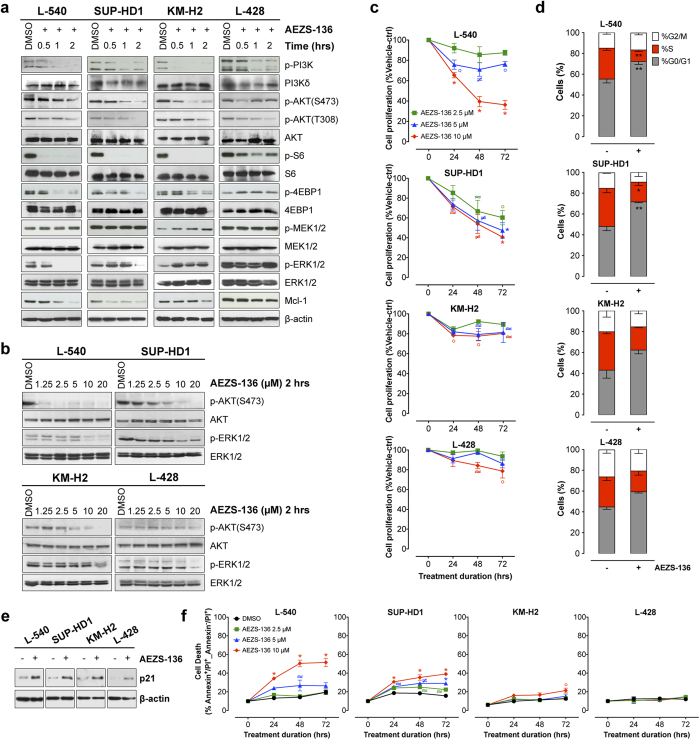
AEZS-136 treatment affects the PI3K/AKT and MAPK pathways, reduces cell proliferation, and increases cell death. (**a**) Immunoblots of extracts from HL cells treated with AEZS-136 (10 μM) or DMSO vehicle for 0.5, 1, or 2 hours. Equal protein loading was confirmed by immunoblotting for β-actin. The experiments were repeated twice with similar results. Representative blots are shown. (**b**) Dose-dependent effect of AEZS-136 (1.25–20 μM) on phosphorylated and total AKT and ERK after exposure to AEZS-136 or DMSO vehicle for 2 hours in HL cells. (**c**) HL cells were exposed to 2.5 μM (green), 5 μM (blue), or 10 μM (red) AEZS-136 for 24, 48, or 72 hours, after which, the cell proliferation was assessed as described in the Methods section. The mean (±SEM) values refer to three independent experiments. *P ≤ 0.0001, ≠*P* ≤ 0.001, °*P* ≤ 0.01, and ≈*P* ≤ 0.05 compared with vehicle-treated cells. (**d**) Cell cycle analysis of AEZS-136 (10 μM)-treated L-540, SUP-HD1, KM-H2, and L-428 cells or vehicle-treated controls after 48 hours. Flow cytometry was performed to measure the cell cycle distribution of treated cells compared with untreated controls. The mean (±SEM) values refer to three independent experiments. *P ≤ 0.05 and **P ≤ 0.01 compared with vehicle-treated cells. (**e**) Immunoblots of extracts from HL cells treated with AEZS-136 (10 μM) or vehicle-treated controls for 48 hours. Equal protein loading was confirmed by immunoblotting for β-actin. The experiments were repeated twice with similar results. Representative blots are shown. (**f**) HL cells were exposed to 2.5 μM (green), 5 μM (blue), and 10 μM (red) AEZS-136 for 24, 48, and 72 hours, after which, the percentage of dead cells (Annexin V+/PI+ plus Annexin V−/PI+) was obtained as described in the Methods section. The mean (±SEM) values correspond to three independent experiments. *P ≤ 0.0001, ≠*P* ≤ 0.001, °*P* ≤ 0.01, and ≈*P* ≤ 0.05 compared with the vehicle-treated control cells.

Preliminary experiments involving a panel of HL cell lines (L-540, SUP-HD1, KM-H2, L-428, HDLM-2; Supplementary Fig. S1) and lymph node biopsies from HL patients (n = 10; Supplementary Fig. S2) demonstrated constitutively high phosphorylation levels of ERK1/2 and AKT with no differences between chemosensitive and chemoresistant patients, suggesting that combined ERK1/2 and AKT inhibition with AEZS-136 might have therapeutic relevance. Time-course experiments showed that ERK1/2 phosphorylation was downregulated in a time-dependent manner in L-540 and SUP-HD1 cells, whereas it was upregulated although at different degrees in KM-H2 and L-428 cells (Fig. 1a). Similarly, the phosphorylation levels of PI3K and its downstream targets (AKT, S6, and 4EBP1) were inhibited in all HL cell lines (Fig. 1a). Since the PI3K/Akt and Raf/MEK/ERK pathways regulate members of the Bcl-2 family of proteins, such as Mcl-1^25^, we examined whether AEZS-136 would affect Mcl-1 expression. AEZS-136 significantly downregulated Mcl-1 expression in the L-540 and SUP-HD1 cells, but not in the KM-H2 or L-428 cells (Fig. 1a). Dose-dependent experiments confirmed that increasing concentrations of AEZS-136 (1.25 to 20 μM) inhibited AKT phosphorilation in all of the tested cell lines, with L-540 and SUP-HD1 being more responsive to AEZS-136 than the KM-H2 and L-428 cells (Fig. 1b). However, AEZS-136 dose-dependently reduced pERK1/2 expression in L-540 and SUP-HD1 cells, whereas only high concentration of AEZS-136 (20 μM) was able to reduce ERK1/2 activation in KM-H2 and L-428 cells.

### *In vitro* antiproliferative activity of AEZS-136

Incubating L-540 and SUP-HD1 cell lines for up to 72 hours with increasing doses of AEZS-136 (2.5–10 μM) resulted in a significant dose- and time-dependent decrease in cell proliferation (Fig. 1c). For both cell lines, the peak of the cytostatic effect was detected upon incubation with 10 μM of AEZS-136 for 72 hours, when the cell proliferation of L-540 and SUP-HD1 cells was significantly (*P* ≤ 0.0001) decreased to 64 ± 4% and 59 ± 1% (mean ± SEM), respectively. Under the same experimental conditions, the cell proliferation of both KM-H2 and L-428 cells was modestly affected by AEZS-136. Cell cycle analysis of L-540 and SUP-HD1 cells exposed to AEZS-136 (10 μM, 48 hours) resulted in a significant reduction of cells in S phase of 63% and 49%, respectively, compared with vehicle-treated controls and significant G0/G1 cell cycle arrest (Fig. 1d) that was associated with the induction of p21 (Fig. 1e). A similar trend was detected in the KM-H2 and L-428 cells.

### Mechanisms of AEZS-136-induced cell death

AEZS-136 treatment resulted in a significant time- and dose-dependent increase in cell death in L-540 and SUP-HD1 cells but not in KM-H2 or L-428 cells (Fig. 1f). Whereas modest effects were detected upon exposure to 2.5 or 5 μM AEZS-136, incubation with 10 μM AEZS-136 for 72 hours significantly increased cell death over vehicle-treated controls in both the L-540 (52 ± 4 vs. 19 ± 3%, P ≤ 0.0001) and SUP-HD1 (40 ± 1% vs. 16 ± 1%) cell lines (*P* ≤ 0.0001; Fig. 1f, Supplementary Fig. S3).

Previous studies investigating the mechanism(s) of MAPK or AKT inhibitor-induced cell death in NHL and HL cell lines have demonstrated the induction of different death pathways involving both caspase-dependent and caspase-independent mechanisms^26^, ROS generation^27^, and JNK activation^28^. AEZS-136-induced cell death in responsive cell lines was not associated with caspase-3 processing or PARP cleavage (Supplementary Fig. S4), and it was not reversed by the pan-caspase inhibitor Z-VAD-FMK (Supplementary Fig. S4), strongly suggesting that AEZS-136 acted via a caspase-independent pathway. Additionally, AEZS-136 treatment led to marked mitochondrial depolarization (up to 40%), which was detected in the cell death-susceptible cells even in the presence of Z-VAD-FMK (Supplementary Fig. S4).

### AEZS-136-induced cell death is associated with ROS generation

**Figure 2:**
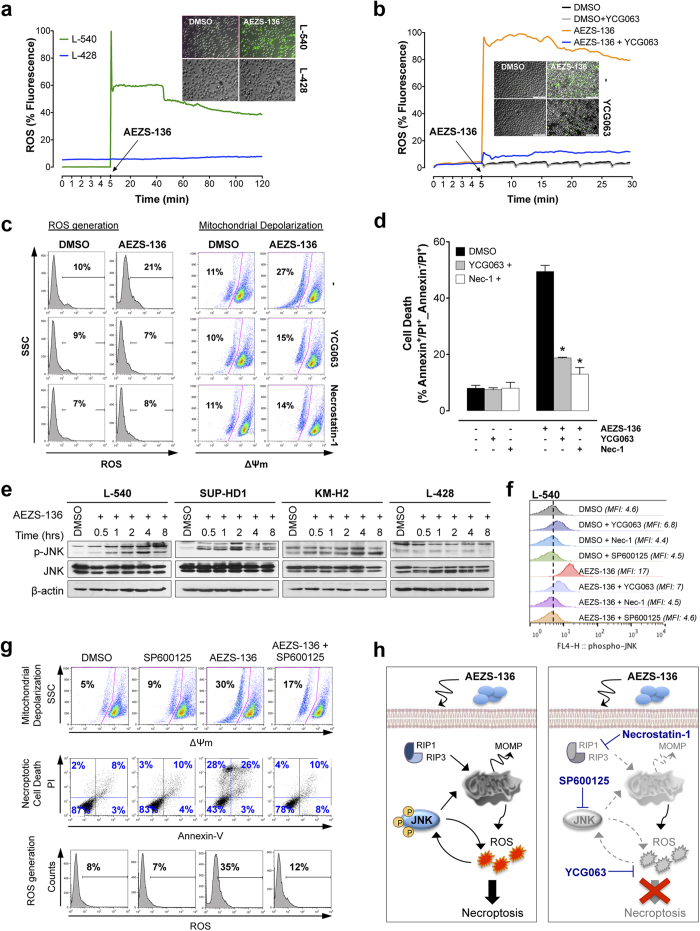
Cell death induced by AEZS-136 correlates with JNK-dependent ROS production. (**a**) Live cell imaging of ROS production in AEZS-136 (10 μM)-treated L-540 and L-428 cells. Representative image captured 120 minutes after drug treatment are shown. Objective lens, 1.0 NA oil objective; original magnification, 40x. (**b**) L-540 cells were pre-labeled with H2DCFDA and then treated with the ROS inhibitor YCG063 (20 μM). After 5 min, the indicated samples were stimulated with 10 μM AEZS-136 or DMSO vehicle. ROS inhibition was quantified using Cell R 5 software. Representative image captured 30 min after drug treatment are shown. Objective lens, 1.0 NA oil objective; original magnification, 40x. (**c,d**) L-540 cells were treated with or without the ROS inhibitor YCG063 (20 μM) or necrostatin-1 (60 μM). After 1 hour, the appropriate samples were stimulated with 10 μM AEZS-136 or DMSO vehicle for 48 hours and (**c**) ROS generation or mitochondrial membrane depolarization were assessed. The experiments were repeated twice with similar results. Representative dot plots are shown. (**d**) Cell death was measured via Annexin-V/PI double-staining. The mean (±SEM) values represent three independent experiments. *P ≤ 0.0001 compared with the cells treated with AEZS-136 and AEZS-136 plus YCG063 or necrostatin-1. Vehicle-treated controls are shown. (**e**) Western blot analysis of extracts from HL cells treated with 10 μM AEZS-136 or DMSO vehicle for the indicated periods. Total levels of JNK and β-actin are shown. (**f**) L-540 cells were treated with or without the ROS inhibitor YCG063 (20 μM), necrostatin-1 (60 μM), or the JNK inhibitor SP600125 (12.5 μM). After 1 hour, the indicated samples were stimulated with 10 μM AEZS-136 or DMSO vehicle for 15 min. JNK expression was measured via flow cytometry. (**g**) L-540 cells were incubated for 1 hour in the presence or absence of the JNK inhibitor SP600125 (12.5 μM). Following exposure to AEZS-136 (10 μM, 24 hours) or DMSO vehicle, mitochondrial membrane depolarization, cell death, and ROS generation were analyzed via flow cytometry. (**h**) Proposed model for the mechanism of action of AEZS-136. Activation is represented by continuous black lines, and inhibition is represented by dotted gray lines.

To further investigate the molecular mechanisms by which AEZS-136 triggered cell death we chose the most and the least cell death-responsive, i.e., L-540 and L-428 cell lines. Upon AEZS-136 exposure, the cell death-susceptible L-540 cells, but not L-428 cells, showed a marked (up to 90%) time-dependent increase in ROS production (Fig. 2a), which was prevented by the ROS inhibitor YCG063 (Fig. 2b). Since ROS act as secondary messengers in the necroptotic pathway^27^,^29^, we analyzed the effects of YCG063 and necrostatin-1 on cells exposed to AEZS-136. The exposure of L540 cells to AEZS-136, in combination with either YCG063 or necrostatin-1, prevented ROS generation and mitochondrial membrane depolarization (Fig. 2c) and reduced cell death to baseline levels (Fig. 2d). Overall, these results indicated that ROS generation are involved in mitochondrial perturbation and necroptotic cell death.

### JNK activation is involved in AEZS-136-induced ROS generation

JNK, a member of the MAPK family, plays an essential role in cell death^30^ and interacts with ROS through a JNK-ROS feedback loop that is involved in ROS-mediated cell death^31^. To investigate the role of JNK in AEZS-136-induced necroptosis via ROS, experiments were performed using the specific JNK inhibitor SP600125, the ROS inhibitor YCG063, and necrostatin-1. AEZS-136 upregulated p-JNK in L-540 and SUP-HD1 cells but not in KM-H2 and L-428 cells (Fig. 2e), a phenomenon that was counteracted by SP600125 (Fig. 2f). Flow cytometry showed that SP600125 prevented AEZS-136-induced mitochondrial depolarization, necroptotic cell death, and ROS generation (Fig. 2g), suggesting JNK-dependent ROS production. Moreover, necrostatin-1 and YCG063 inhibited the expression of p-JNK (Fig. 2f), supporting the existence of a JNK-ROS loop in AEZS-136-induced HL necroptosis (Fig. 2h).

### AEZS-136 modulates gene expression

To gain further insights into the molecular mechanism(s) of action of AEZS-136, the gene expression profiles of vehicle-treated controls and AEZS-136-treated cell lines were analyzed. Supervised hierarchical clustering of HL cell lines demonstrated that AEZS-136 exposure resulted in early effects, as shown by the significantly modulated genes detected after a 2-hour incubation (Supplementary Fig. S5), with a substantial increase in significantly modulated genes detected after 24 hours (Supplementary Fig. S5). A Venn diagram showed that the majority of the 2-hour modulated genes remained modulated at the 24-hour time point (L-540 and L-428 cell lines), and almost all genes were concordantly modulated (upregulated or downregulated) at both time points (Supplementary Fig. S5). These changes were validated via qRT-PCR using selected genes (Supplementary Fig. S6). Biological processes significantly involved both at early (2 hours) and late (24 hours) time points included signal transduction and kinase activity, cell cycle, and cell death, as well as transcription and translation (Supplementary Fig. S7, Supplementary Table S1).

**Figure 3:**
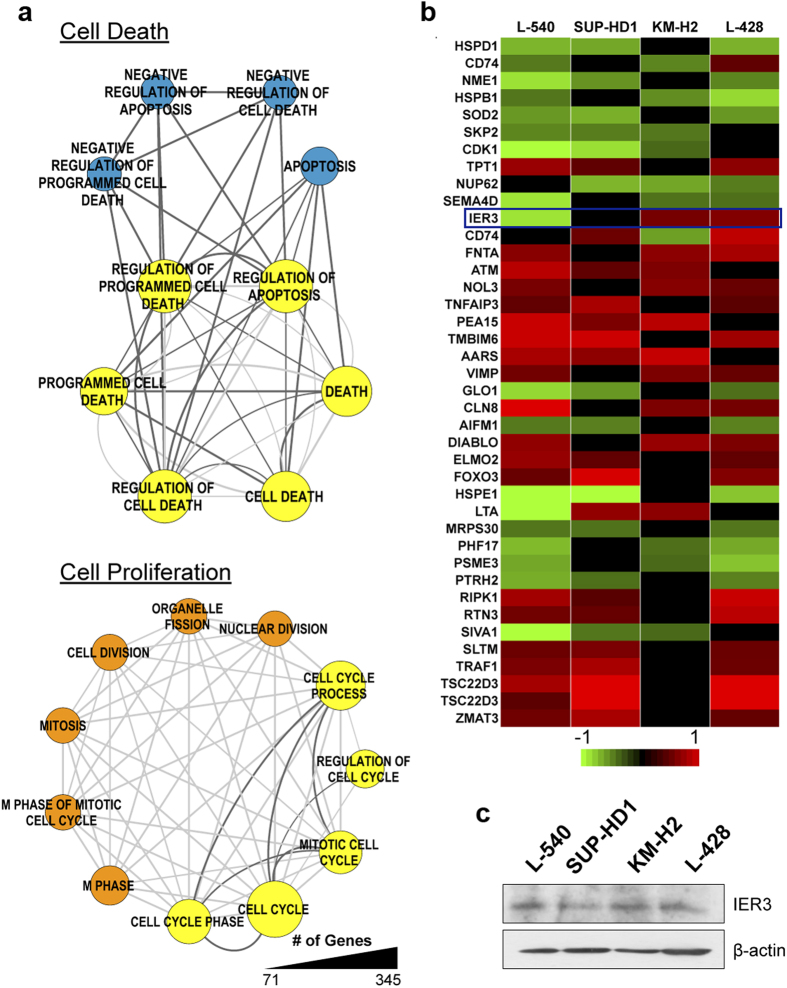
Specific and common biological processes involved in AEZS-136 sensitivity or resistance. (**a**) The enriched biological processes listed in Supplemental Table 2A,B were analyzed using the Cytoscape EnrichmentMap plugin and were grouped into two main categories: Cell Death and Cell Cycle. Specific L-540/SUP-HD1 cell-associated and KM-H2/L-428 cell-associated biological processes are indicated by orange and blue nodes, respectively; common processes are indicated by yellow nodes. The light and dark gray edges show the interaction between the L-540/SUP-HD1 cell-associated and the KM-H2/L-428 cell-associated biological processes, respectively. The node size reflects the number of genes. (**b**) Heat map of genes listed in Supplemental Table 3. Shown are the genes that were differentially expressed in at least three HL cell lines. Oppositely modulated gene between the L-540/SUP-HD1 cells and between the KM-H2/L-428 cells is indicated by blue rectangle. (**c**) Western blot analysis of IER3 basal expression in HL cell lines. Equal protein loading was confirmed by immunoblotting for β-actin.

To explain the intrinsic resistance to AEZS-136-induced necroptotic cell death observed in the KM-H2 and L-428 cell lines, we used data from the 24-hour time point to perform a stringent gene ontology analysis with differentially expressed genes. By comparing gene ontology results, we identified specific and common biological processes involved in AEZS-136 sensitivity or resistance (Fig. 3a, Supplementary Table S2). These data led to the identification of a subset of genes involved in cell death and cell proliferation (Supplementary Table S2). Genes involved in the “cell cycle” were specifically enriched in L-540 and SUP-HD1 cells (Fig. 3a, orange nodes), whereas genes involved in the “cell cycle” and “regulation of cell death” were enriched in all HL cells (Fig. 3a, yellow nodes). Interestingly, negative “regulation of cell death, programmed cell death and apoptosis” were specifically enriched in KM-H2 and L-428 cells (Supplementary Table S3 and Fig. 3a, blue nodes), suggesting a potential role of these processes in resistance to AEZS-136 treatment. Considering only the genes enriched in the blue nodes (156 genes, Supplementary Table S3 and Fig. 3a), we selected genes expressed in at least three cell lines (40 genes, Fig. 3b) and subsequently focused on genes showing an opposite regulation between the cell death-resistant (KM-H2 and L-428) and cell death-sensitive (L-540 and SUP-HD1) cell lines (the absence of regulation was not considered). Interestingly, the immediate early response 3 (*IER3*) gene was the only gene that fulfilled our filtering criteria. *IER3* was significantly upregulated by AEZS-136 in the cell death-resistant cell lines, whereas it was downregulated in the AEZS-136-sensitive L-540 and SUP-HD1 cell lines (Fig. 3b), regardless of the similar IER3 basal expression in all HL cell lines (Fig. 3c).

**Figure 4:**
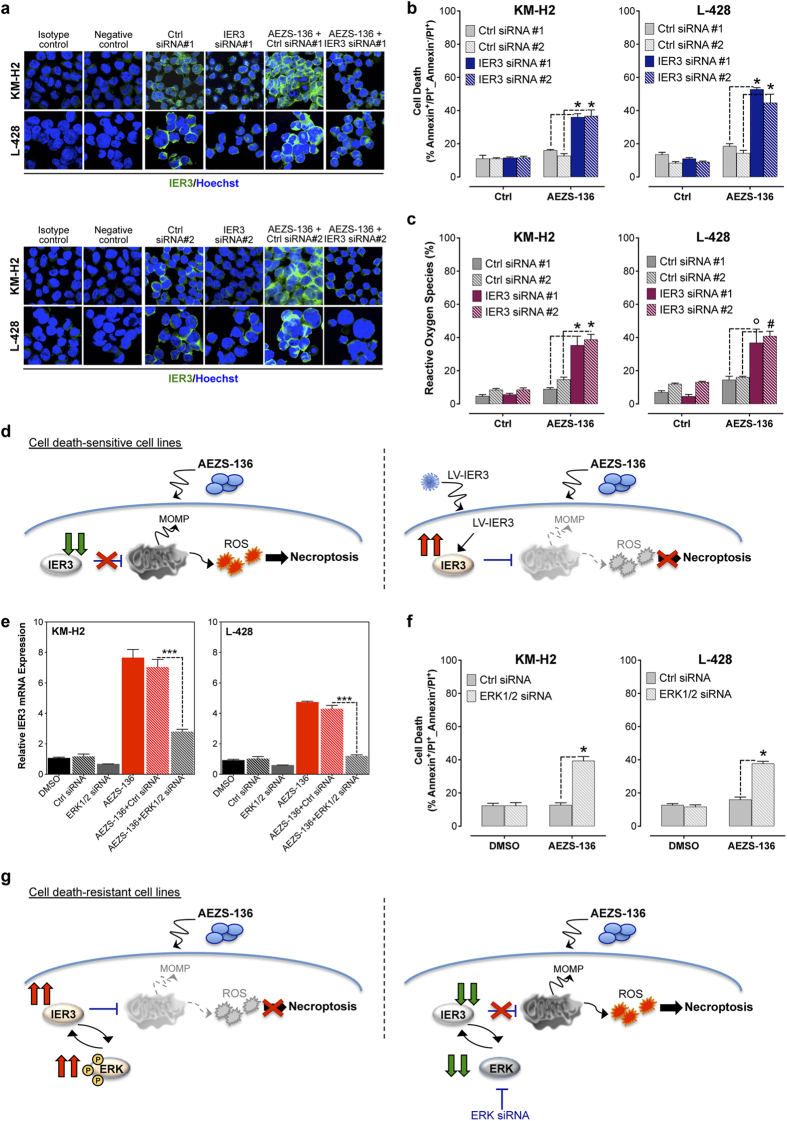
IER3-directed siRNA enhances AEZS-136-induced ROS production and cell death. (**a**) KM-H2 and L-428 cells were transfected with IER3 siRNA #1 and #2 or control siRNA 1 and #2 and treated with AEZS-136 or DMSO vehicle. After 24 hours, the efficiency of IER3-directed siRNA inhibition was analyzed via immunofluorescence using an IER3-specific antibody. Cell nuclei (*blue*) were detected using Hoechst dye. Objective lens, 1.0 NA oil objective; original magnification, 60x; zoom, 3x; scale bar, 20 μm. In the isotype control, the cells were incubated with a Goat IgG isotype control (Alexa Fluor 488) (antibodies-online GmbH, Germany, EU). In the negative control, the IER3 antibody was omitted, and the cells were incubated with secondary antibody alone. (**b**) Cell death after 48 hours was measured as described in the Methods section. The mean (±SEM) values correspond to three independent experiments. *P ≤ 0.0001 compared with control siRNA. (**c**) The generation of ROS after 48 hours was measured. *P ≤ 0.0001, #*P* ≤ 0.001, °*P* ≤ 0.01 compared with control siRNA. (**d**) Proposed model for ectopic IER3 upregulation: (*left panel*) AEZS-136 causes IER3 downregulation in the cell death-sensitive cell lines, leading to necroptotic cell death. (*right panel*) Lentiviral IER3 transduction ectopically increased IER3 expression, preventing AEZS-136-induced necroptotic cell death. Activation is represented by continuous lines, and inhibition is represented by dotted lines. (**e**) KM-H2 and L-428 cells were transfected with ERK1/2 siRNA or control siRNA and treated with AEZS-136 or DMSO vehicle. After 48 hours, the efficiency of ERK1/2-directed siRNA inhibition on IER3 was analyzed via real-time RT-PCR. PCR data were normalized to the expression of β2-microglobulin as a housekeeping gene. (**f**) Cell death after 48 hours was measured as described in the Methods section. The mean (±SEM) values correspond to three independent experiments. ***P ≤ 0.0001. (**g**) Proposed model for the mechanism of IER3 upregulation: (*left panel*) AEZS-136 causes pERK1/2 upregulation in the cell death-resistant cell lines. (*right panel*) ERK siRNA restores AEZS-136-induced necroptotic cell death. Activation is represented by continuous lines, and inhibition is represented by dotted lines.

Next, we investigated the impact of IER3 on cell survival in the AEZS-136-resistant cell lines. We depleted KM-H2 and L-428 cells in IER3 using two specific siRNAs (Fig. 4a, Supplementary Fig. S8), and cell death (Fig. 4b) and ROS production (Fig. 4c) were monitored after AEZS-136 treatment. IER3 depletion in AEZS-136-treated cells resulted in massive increase in cell death (Fig. 4b) and ROS production (Fig. 4c), suggesting that IER3 is a cell death-protective protein against AEZS-136 treatment. Finally, to confirm that IER3 is involved in the resistance to AEZS-136 treatment, a genetic rescue was designed. AEZS-136-sensitive L-540 cells transduced with lentivirus designed to overexpress IER3 (Supplementary Fig. S9) were treated with AEZS-136, and cell death was monitored. No cell death prevention was observed with the Scrambled vector, whereas a 50% cell death reduction was observed in LV IER3-transduced L-540 cells (Supplementary Fig. S9). Together, these data strongly suggest that IER3 upregulation mediates resistance to AEZS-136 (Fig. 4d).

Since we have shown that AEZS-136 induced upregulation of both IER3 and phospho-ERK expression in the AEZS-136-resistant cell lines, we investigated the IER3/ERK1/2 relationships. When AEZS-136-treated KM-H2 and L-428 cells were exposed to the ERK1/2 siRNA, both ERK1/2 (Supplementary Fig. S10) and IER3 levels were reduced (Fig. 4e), leading to significantly increased cell death (Fig. 4f). Additionally, IER3 silencing was associated with decreased ERK expression (Supplementary Fig. S10). In keeping with previously reported data showing that IER3 and ERK regulate each other’s activities, our data further support that IER3 acts both as an ERK downstream effector affecting survival and as a regulator of ERK activation (Fig. 4g)^32^.

### Effects of AEZS-136 on the growth of HL subcutaneous xenografts

**Figure 5:**
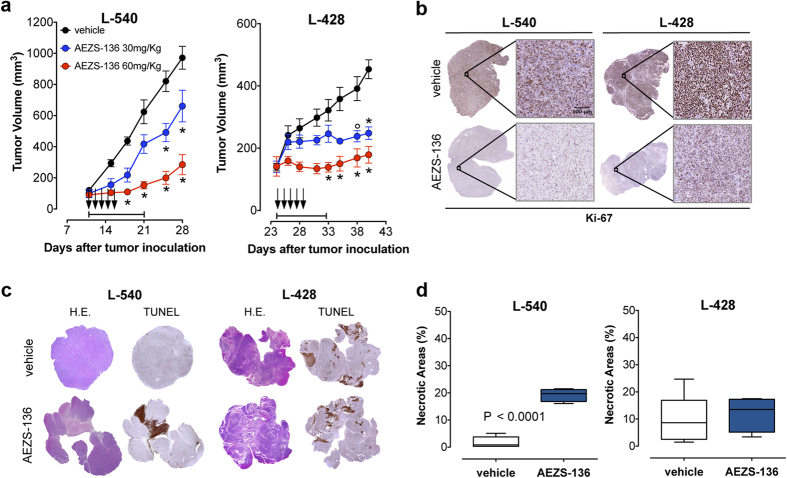
Effect of AEZS-136 on tumor growth and tumor necrosis. (**a**) NOD/SCID mice bearing 100-mm^3^ SC tumor nodules were randomly assigned to receive 10-day treatment with AEZS-136 (30 mg/kg, 5 days/week for 2 weeks, PO, blue or 60 mg/kg, 5 days/week for 2 weeks, PO, red) or vehicle control treatment (black). Each experiment was performed on at least two separate occasions using 5 mice per treatment group. For each cell line, the treatment duration is indicated by horizontal capped black lines (days 11–21 and days 24–32 for the L-540 and L-428 xenografts, respectively). The mean (±SEM) tumor volumes are shown. *P ≤ 0.0001 and °*P* ≤ 0.01 compared with the controls. (**b**) Ki-67 staining of the L-540 and L-428 tumors treated with AEZS-136 (60 mg/kg/day, 5 days) or the vehicle control. In the Ki-67-stained section, brown staining represents a positive signal within the tumor (the blue cells are Ki-67-negative, living cells). Objective lens, 0.75 NA dry objective; original magnification, 20x. Scale bar, 100 μm. (**c**) NOD/SCID mice bearing SC tumor nodules (100 mm^3^) were randomly assigned to receive 5 days of treatment with AEZS-136 (60 mg/kg/day, PO) or the vehicle control. Representative histological images of entire tumor sections. Tumor tissue morphology was detected via H&E staining. The tumor necrotic areas were detected via TUNEL staining and were visualized as brown. Objective lens, 0.08 NA dry objective; original magnification, 2x. (**d**) Digitally acquired TUNEL-stained sections were analyzed using ImageJ software for the quantification of the percentage of tumor necrosis. At least three sections from different animals were analyzed per treatment group. The boxes extend from the 25^th^ to the 75^th^ percentiles, the lines indicate the median values, and the whiskers indicate the range of the values. *P* ≤ 0.0001 compared with the controls.

AEZS-136 affected in a dose-dependent manner not only the *in vivo* growth of the AEZS-136-sensitive L-540 xenografts [tumor growth inhibition (TGI) = 72% at 60 mg/kg] but also the growth of the AEZS-136-resistant L-428 xenografts (TGI = 60% at 60 mg/kg) (Fig. 5a). To understand the unexpected results detected for L-428 xenografts, we first evaluated Ki-67 expression and tumor necrosis in both L-540 and L-428 tumor xenografts. Ki-67 expression was strongly reduced in both tumor xenografts (Fig. 5b), demonstrating that AEZS-136 inhibited cell proliferation as expected based on *in vitro* data. Tumor necrosis was increased by 10-fold compared with controls (19 ± 2% vs. 1.7 ± 2%, *P* ≤ 0.0001) in L-540 nodules, whereas no evidence of *in vivo* cytotoxicity could be detected in the AEZS-136-unresponsive L-428 tumors (Fig. 5c,d), showing that L-428 cells were resistant to AEZS-136-induced cell death both *in vitro* and *in vivo*.

**Figure 6:**
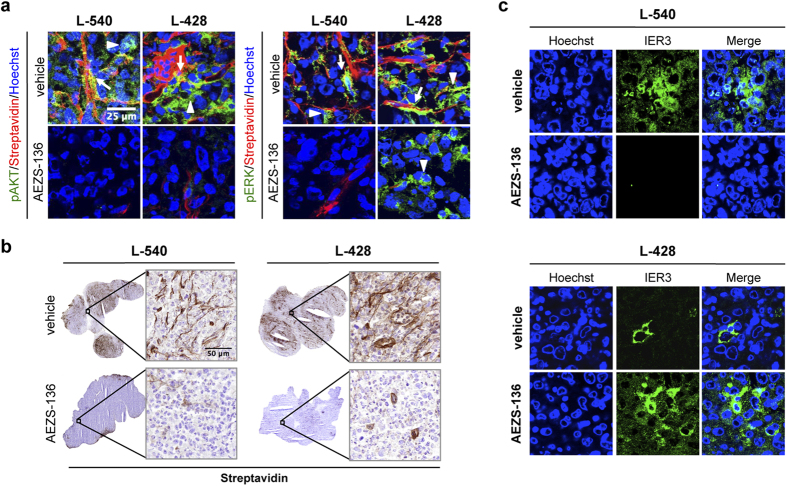
Antivascular activity by AEZS-136 and quantification of IER3 expression in the entire tumor sections. (**a**) Ninety-six hours after treatment with AEZS-136 and 3 hours after the last AEZS-136 administration, NOD/SCID mice were IV injected with 0.2 ml of sulfo-NHS-LC-biotin (5 mg/ml) to biotinylate the tumor vasculature. Tumors were then excised, embedded in cryoembedding compound and freshly snap-frozen in isopentane. Tumor sections were double-stained with streptavidin (red) and phospho-ERK1/2 (green) or phospho-AKT (green). Nuclei were detected with Hoechst (blue). Arrows indicate phospho-ERK1/2 or phospho-AKT expression by endothelial cells; arrowheads indicate phospho-ERK1/2 or phospho-AKT expression by tumor cells. Representative images are shown. Objective lens, 1.0 NA oil objective; original magnification, 60x. Scale bar, 25 μm. (**b**) NOD/SCID mice bearing SC tumor nodules (100 mm^3^) were randomly assigned to receive 5 days of treatment with AEZS-136 (60 mg/kg/day, PO) or the vehicle control. Representative histological images of tumors from *in vivo* biotinylated mice receiving AEZS-136 or vehicle-control are shown. Biotinylated endothelia were revealed by sequentially incubating frozen sections with HRP-streptavidin and 3,3′-diaminobenzidine for light microscopy analysis. Objective lens, 0.75 NA dry objective; original magnification, 20x. Scale bar, 50 μm. (**c**) Representative confocal images of tumors from the vehicle-treated and AEZS-136-treated animals that were processed via double immunofluorescence staining are shown. Cell nuclei (*blue*) were detected using Hoechst dye. IER3 expression (*green*) was detected using the anti-IER3 antibody and an Alexa Fluor 488-conjugated secondary antibody. Cell nuclei (*blue*) were detected using Hoechst dye. Objective lens, 1.0 NA oil objective; original magnification, 60x; zoom, 3x.

We then hypothesized that the marked AEZS-136-induced TGI in both L-540 and L-428 xenografts might be linked to the inhibition of tumor angiogenesis triggered by activation of the PI3K/AKT and RAS/MEK/ERK pathways^33^. AEZS-136 strongly reduced AKT and ERK1/2 phosphorylation in tumor and vascular cells in both HL cell lines (Fig. 6a). As compared with controls, both L-540 and L-428 tumors showed vessels smaller in length, lacking in sproutings and substantially less arborized and dishomogeneously distributed within the tumor (Fig. 6b), suggesting that AEZS-136 affected not only tumor cells but also tumor angiogenesis. Antiangiogenetic effects of AEZS-136 might therefore significantly contribute to the *in vivo* antitumor activity detected in the AEZS-136-resistant cell line. Consistent with the *in vitro* data, we detected marked IER3 downregulation in L-540 AEZS-136-treated tumors, whereas we found strong IER3 upregulation in L-428 AEZS-136-treated tumors (Fig. 6c), compared with controls. Taken together, these results suggest that the *in vivo* antitumor efficacy of AEZS-136 is associated with tumor cell death, resulting from decreased IER3 expression in tumor cells.

## Discussion

The PI3K/AKT/mTOR and RAS/MEK/ERK pathways are two of the most important signaling cascades that are dysregulated by genetic lesions or hyperactivation in the majority of human cancers^34^. Carracedo and colleagues demonstrated the crossover and compensatory mechanisms that exist between these two important pathways^35^. Furthermore, genetic and pharmacological analyses have shown that the RAS/RAF1/MEK1/MEK2 arm of the MAPK signaling pathway is responsible for ERK/AKT signaling downstream of S6K1/PI3K when mTORC1 activity is lost, indicating that targeting the MAPK pathway might improve the efficacy of mTORC1 inhibition^36^. Therefore, the concept of rationally combining molecular targeted therapeutics, to disrupt either a specific pathway at different levels or two different pathways acting in a complementary manner, has attracted considerable interest^37^.

In this study, we elucidated the molecular mechanism(s) by which the PI3K/ERK dual inhibitor AEZS-136 exerted a variety of antitumor effects both *in vitro* and in HL xenografts. These antitumor effects were achieved using AEZS-136 concentrations of 10 μM. While pharmacokinetics data are not available in humans, studies in cynomolgus monkeys have shown that a 10 μM plasma concentration can be reached upon oral AEZS-136 administration at 90–300 mg/kg (I. Seipelt, Aeterna Zentaris, personal communication). Potent cell death induction by AEZS-136 was associated with the transcriptional modulation of cell cycle- and cell death-associated pathways, ultimately causing caspase-independent necroptotic cell death, which was associated with a striking increase in ROS production, with JNK activation, and with mitochondrial injury. These findings were consistent with our previous studies demonstrating the synergistic anti-HL activities of sorafenib and perifosine, including the inhibition of the MAPK and AKT pathways and the promotion of necroptosis^27^.

We investigated the mechanism of HL cell resistance to the necroptotic cell death induced by the concomitant PI3K and ERK inhibition. We show that elevated IER3 and phospho-ERK expression were associated with decreased ROS levels in the AEZS-136-resistant HL cell lines (i.e., KM-H2 and L-428). The stress-inducible gene IER3 was indeed identified as the primary mediator of resistance to AEZS-136-induced oxidative death both *in vitro* and *in vivo*^38^. This molecular mechanism is similar to what observed with drug resistance to RAF inhibitors which is due to hyperactivation rather than inhibition of the ERK cascade. In fact, non-saturating concentrations of the inhibitor lead to a partial inhibition of the target which in turn results in its strong hyperactivation^39^. IER3 gene play a role in the ERK signaling pathway by inhibiting the dephosphorylation of ERK by phosphatase PP2A-PPP2R5C holoenzyme^40^. Acts also as an ERK downstream effector mediating survival^32^. As a member of the NUPR1/RELB/IER3 survival pathway, may provide pancreatic ductal adenocarcinoma with remarkable resistance to cell stress, such as starvation or gemcitabine treatment^41^.

IER3 regulates cell cycle progression, cell proliferation, and cell death^42^. Additionally, in cells under stress IER3 participates in multiple cell signaling pathways, such as ERK signaling prolongation, nuclear Mcl-1 accumulation, and DNA repair^32^,^43^. Notably, IER3 depletion using a specific siRNA significantly restored AEZS-136-induced oxidative cell death in these cell lines, as well as decreased ERK phosphorylation, indicating that the activation of this stress-inducible gene played a major role in the mechanism underlying resistance to AEZS-136. Novel findings have correlated IER3 overexpression with mitochondrial F_1_F_o_-ATPase inhibitor (IF1) degradation, increased ATP synthase/ATPase activity and sustained Δψm in a polarized state, thus protecting cells from mitochondrion-dependent apoptosis^44^. By contrast, a lack of IER3 stabilizes IF1 and decreases F1Fo-ATPase activity and has been associated with increased ROS production^44^,^45^. Thus, we can speculate that IER3 overexpression blocks AEZS-136-induced cell death by inhibiting ROS generation and inducing pERK upregulation.

AEZS-136 induced ROS generation and a potent oxidative stress associated with necroptosis^46^. There are many studies that provide evidence for a role of ROS as critical mediators of necroptosis^29^. Schenk and Fulda showed that necroptotic cell death induced by treatment with TNFα and the Smac mimetic BV6 depends on ROS as critical regulators of necroptotic signaling and cell death^47^. Moreover, TNFα-induced ROS generation and cytotoxicity were inhibited by a mitochondrial respiratory chain inhibitor but not by a NADPH oxidase inhibitor, supporting that mitochondrial rather than cytosolic ROS are involved in mediating TNFα-induced necroptosis^48^. Additional studies on components of the mitochondrial permeability transition (MPT) pore underlined the link existing between ROS production and necroptotic cell death. Opening of the MPT pore has been shown to result in defective oxidase phosphorylation, generation of ROS and cell death^49^. The MPT is composed of cyclophilin D and genetic evidence derived from cyclophilin D knockout mice underscored the critical role of cyclophilin D in the regulation of necrotic cell death^49^. Also, TNFα-induced necroptosis has been reported to be partially reduced in mouse embryonic fibroblasts derived from cyclophilin D knockout mice^50^.

ROS are believed to oxidize MAPK phosphatases (MKPs), the normal function of which is to downregulate JNK pathway signaling^51^, thereby resulting in prolonged JNK activation and cell death. *In vitro*, AEZS-136 significantly increased ROS levels, mitochondrial depolarization, and JNK phosphorylation in AEZS-136-sensitive cells but not in AEZS-136-resistant cells. These events were prevented by treatment with the ROS inhibitor YCG063 or necrostatin-1, suggesting that ROS production was a prerequisite for AEZS-136-mediated necroptotic cell death and strongly supporting the involvement of JNK in AEZS-136-induced cell death. In agreement with previously published data^52^ showing the involvement of JNK in necroptotic cell death, we found that JNK inhibition using the JNK inhibitor SP600125 prevented AEZS-136-induced mitochondrial membrane depolarization, ROS generation, and necroptotic cell death, strongly supporting the reciprocal activation of ROS and JNK via a positive feedback loop ultimately causing necroptotic cell death.

Although HL cell lines have been instrumental in identifying genetic lesions and deregulated signaling in HRS cells, the differences between these cell lines and primary HRS cells should also be considered when extrapolating to patients the results obtained in HL cell lines^53^. New preclinical models are needed to validate the therapeutic potential of AEZS-136. The *in vivo* xenograft data reported herein suggested that AEZS-136 induced antivascular effects and reduction of tumor growth in all HL models. According to our *in vitro* data, tumor necrosis and IER3 downregulation were shared only by cell death-sensitive AEZS-136 cells, suggesting a key role of IER3 in AEZS-136-iduced resistance. Notably, compared with treatment with each agent alone, the combined treatment with perifosine and sorafenib induced severe IER3 inhibition in the AEZS-136-responsive HL cells (Supplementary Fig. S11). Therefore, it is tempting to speculate that IER3 downregulation correlates with the effectiveness of concomitant PI3K and ERK pathway inhibition.

Recently, the role of IER3 in patients with myelodysplastic^54^ and Sézary^45^ syndromes has been described. Mice overexpressing IER3 spontaneously developed T-cell lymphoma, likely due to altered apoptosis sensitivity (including susceptibility to death receptor ligation), cell cycle progression, and/or proliferation^55^. Collectively, our data suggested that IER3 expression in tumor samples after AEZS-136 treatment might serve as a biomarker of treatment response allowing the identification of AEZS-136 unresponsive patients treatment^56^. The next step would be to understand better the cause of increased IER3 expression in AEZS-136-resistant HL to identify the optimal combination therapy, including therapies that would induce apoptosis irrespective of IER3 levels (cisplatin, gemcitabine, or doxorubicin) or that would increase ROS levels^57^. Indeed, we are currently investigating this potential strategy to restore the efficacy of AEZS-136 therapy in HL cells displaying increased IER3 expression.

In conclusion, our data suggested that AEZS-136 might be clinically effective for HL and other hematological malignancies characterized by PI3K and ERK phosphorylation. Our findings could have significant implications for the clinical development of AEZS-136.

## Methods

### Reagents

AEZS-136 was provided by Æterna Zentaris (Frankfurt, Germany, EU). Z-VAD-FMK and necrostatin-1 (R&D Systems, Minneapolis, MN, USA). Tetramethylrhodamine ethyl ester (TMRE) and 6-carboxy-2′,7′-dichlorodihydrofluorescein diacetate di(acetoxymethyl ester) (H2DCFDA) (Invitrogen, Milan, Italy, EU). YCG063 and SP600125 (Merck Millipore, Billerica, MA, USA). Stock solutions of each reagent were prepared in 100% dimethylsulfoxide (DMSO) and subsequently a working solution was obtained by diluting (at least 1:1000, v/v) in RPMI.

### Cell lines

The L-540, SUP-HD1, KM-H2 and L-428 cell lines were purchased from the German Collection of Microorganisms and Cell Cultures (DSMZ, Braunschweig, Germany, EU). Cell lines were cultured in RPMI-1640 supplemented with 20% fetal bovine serum (FBS). The identity of the cell lines was authenticated by multiplex PCR of minisatellite markers that revealed a unique DNA profile. Cell cultures were also tested for the presence of Mycoplasma (Mycoplasma Detection Kit, Invivogen) before the initiation of this study.

### Cell proliferation assay

Cell proliferation was evaluated by Quick Cell Proliferation Assay Kit II (BioVision Research Products, CA, USA), following the manufacturer’s instructions. Cell proliferation inhibition rate was calculated as the percentage of proliferation reduction in comparison to the proliferation of vehicle-controls.

### Cell death assay

Dead cells (Annexin-V+/PI+ and Annexin-V-/PI+ cells) were detected via Annexin-V/PI double-staining and flow cytometry^58^. Cells were analyzed using a FACSCalibur flow cytometry system (Becton-Dickinson, San Jose, CA, USA). The data were analyzed using FlowJo software (Tree Star, Inc., Ashland, OR, USA), version 8.7.1.

### Cell cycle analysis

The cells were cultured under the appropriate conditions for 48 hours, fixed in 70% ethanol, and stained with 2.5 μg/ml PI (Calbiochem, Darmstadt, Germany). Cell cycle status was measured using a FACSCalibur flow cytometry system (BD) and was analyzed using FlowJo software.

### Western blot analysis

Western blot analysis was performed using antibodies against Mcl-1, JNK (56G8), pJNK (81E11), pMEK (41G9), MEK (D1A4), pERK1/2 (197G2), ERK1/2 (137F5), pAKT (S473) (193H12), pAKT (T308) (244F9), AKT, pS6 (Ser235/236), S6, p4EBP-1 (Thr70), 4EBP-1, pPI3K p85 (Tyr458)/p55(Tyr199) (Cell Signaling Technology, Danvers, MA, USA), PI3K p110 delta (Novus Biologicals), caspase-3, p21, IER3 (Santa Cruz Biotechnologies, San Diego, CA, USA), and poly(ADP-ribose) polymerase (PARP) (BD).

### Measurement of the mitochondrial membrane potential (ΔΨ_m_)

Mitochondrial membrane depolarization was determined using the fluorescent probe TMRE (Invitrogen) and was analyzed by flow cytometry.

### Measurement of ROS

ROS generation was detected as previously described^22^. An Olympus IX81 confocal microscope and an Olympus oil immersion objective lens (40x/1.3 Plan-Apochromat) (Olympus, Milan, Italy, EU) were used. The images were acquired (1 frame every 30 seconds, 140 frames total) and processed using Xcellence software (Olympus).

### Genome-wide expression profiling

The cells were treated with AEZS-136 (10 μM) for 2 or 24 hours. RNA integrity and the purity of the treated cells were assessed using a Bioanalyzer (Agilent Technologies, Milan, Italy, EU). Hybridization to an Illumina Microarray (Illumina, San Diego, CA, USA) was performed. The gene expression data are available in the NCBI Gene Expression Omnibus database (accession numbers GSE71150 and GSE58899). Robust spline normalization and L2T were performed in R software, using the lumi package from Bioconductor open source software (http://www.bioconductor.org/). The significantly differentially expressed genes were identified using Student’s t-test adjusted according to the Benjamini-Hochberg (bh) procedure by considering account transcripts displaying Padj <0.05. Furthermore, transcripts displaying a log_2_ fold-change in expression of >−0.4 or <0.4 were filtered out. One-way hierarchical clustering of the significantly modulated genes was performed on the gene-wise median-centered normalized intensities, using Cluster software, version 3.0. Centered correlation and complete linkage clustering were used as the distance metrics. Heat maps were visualized using Java Treeview software. Functional annotation of the significantly differentially expressed genes identified, based on microarray analysis, was performed using DAVID Bioinformatics Resources (david.abcc.ncifcrf.gov). Cytoscape software, version 2.8 (http://www.cytoscape.org/), and the EnrichmentMap plugin were used to perform network analysis.

### Quantitative real-time PCR (qRT-PCR)

mRNA isolation, cDNA reverse transcription, and qRT-PCR were performed as described previously^27^. qRT-PCR was performed using TaqMan gene expression assays for *DCTPP1* (Hs00225433_m1), *HNRNPAB* (Hs00954054_g1), *CCNA2* (Hs00996788_m1), *MAPK3* (Hs00385077_m1) and *IER3* (Hs04187506_g1) (Applied Biosystems, Foster City, CA, USA) and an ABI ViiA7 sequence detection system (Applied Biosystems). The data were normalized to the expression levels of the housekeeping gene β2-microglobulin.

### siRNA-mediated gene silencing

KM-H2 and L-428 cells were transfected with 100 nM of either Silencer Select IER3-directed small interference RNA (siRNA) or Silencer Select Negative Control siRNA (Ambion, Carlsbad, CA, USA); SignalSilence p44/42 MAPK (ERK1/2) siRNA or Control siRNA (Cell Signaling Technology) according to the manufacturer’s instructions. Twenty-four hours after transfection, the cells were treated with 10 μM AEZS-136. The effects of gene silencing were evaluated by immunofluorescence, mRNA and cell death as described above after 24 and 48 hours of drug exposure.

### Lentiviral transduction

Lentiviral vectors including a lentivirus empty cassette (LV-Scrambled) and a lentivirus encoding the human IER3 gene (LV-IER3) were purchased from (Cellomics Technology, MD, USA). Transduction was performed in 48-well plates. L-540 was seeded at (0.4 × 10^6^ cells/ml), 10^5^ cells per well. One day later, the cells were transduced with a final multiplicity of infection (MOI) of 20 (PFU)/cell of both LV-Scrambled and LV-IER3 for 24 hrs. Six days after transduction, the efficiency was measured by detecting IER3 using immunofluorescence analysis.

### Activity of AEZS-136 in tumor-bearing non-obese, diabetic/severe combined immunodeficient (NOD/SCID) mice

Six- to eight-week-old NOD/SCID mice with body weights of 20–25 g were purchased from Charles River (Milan, Italy, EU) and were xenografted with L-540 or L-428 cells. Animal experiments were performed according to EU 86/109 Directive (D.L. 116/92 and following additions) and approved by the institutional Ethical Committee for Animal Experimentation of the Humanitas Clinical and Research Center. The activity of AEZS-136 was analyzed in the subcutaneous (SC) xenograft models. L-540 (25 × 10^6^ cells/mouse) or L-428 (20 × 10^6^ cells/mouse) cells were inoculated into the left flank of each mouse. When the tumor volume reached approximately 100 mm^3^, mice were randomly assigned to receive either short- or long-term treatment with AEZS-136 [working solution containing 10% DMSO (v/v)] or vehicle [10% DMSO solution (v/v)]. As previously reported^26^, mice treatment with a 10% DMSO solution failed to affect tumor cell signaling likely due to *in vivo* dilution of DMSO as well as its rapid metabolic clearance. The short-term treatment consisted of AEZS-136 (60 mg/kg/5 days, PO) and was used to assess necrotic areas and tumor cell proliferation. *In vivo* biotinylation of tumor vasculature was performed when appropriate 3 hours after the last AEZS-136 administration. Tumor nodules were then excised and processed for histological analysis. The long-term treatment consisted of AEZS-136 (30 or 60 mg/kg/5 days for 2 wks, PO). The tumor volumes were calculated using the following formula: (a × b^2^)/2, where a and b represent the longest and shortest diameters, respectively. Tumor growth inhibition (TGI) was defined as (1 − [T/C] × 100), where T and C represent the mean tumor volumes in the treated and untreated control groups, respectively. Each experiment was performed on at least two separate occasions using five mice per treatment group.

### Immunofluorescence and confocal microscopy

Cells were cytospun onto positively charged glass slides, fixed with cold acetone, and blocked with 2% BSA. Otherwise, epitope retrieval was performed on formalin-fixed, paraffin-embedded (FFPE) nodules using EDTA buffer. The samples were incubated in a goat anti-human IER3 (1:50) (Santa Cruz Biotechnology, Heidelberg, Germany, EU), -pAKT (S473), -pERK1/2 and -pS6 (Cell Signaling Technology, Leiden, The Netherlands, EU) antibodies, followed by the appropriate Alexa Fluor 488- and 568-conjugated secondary antibody (Invitrogen). Cryostat sections (4-μm thick) of *in vivo* biotinylated tumor nodules were double-stained with Alexa Fluor 568-streptavidin (Invitrogen) and anti-human phospho-AKT (Ser473) or phospho-ERK1/2 (Cell Signaling). Tumor/endothelial p-AKT or p-ERK1/2 expression was revealed by Alexa Fluor 488-conjugated secondary antibody (Invitrogen). Finally, the sections were labeled with Hoechst nuclear dye (1:10,000) (Invitrogen) and examined under a confocal microscope (Nikon A1R, Nikon Instruments, Florence, Italy, EU). Image processing was performed using NIS-Elements AR software (Nikon Instruments).

### Histological analysis and immunohistochemistry

Sections (2-μm thick) from FFPE human tumor xenografts and lymph node biopsies obtained from consenting patients at the time of diagnostic work-up were stained with hematoxylin and eosin (H&E) or with anti-human Ki-67 antibody (Dako, Milan, Italy, EU), -pAKT (S473), -pAKT (T308), -pERK1/2 or -pS6 (Cell Signaling) antibodies. Chemosensitive and chemorefractory patients were defined according to the criteria of German Hodgkin Lymphoma Study Group^59^. Study was approved by the by the institutional Ethical Committee of the Humanitas Clinical and Research Center. Tumor necrosis was detected via TUNEL staining (Roche, Milan, Italy, EU). Cryostat sections (4-μm thick) of *in vivo* biotinylated tumor nodules were processed as previously described^28^. The sections were examined under a light microscope (IX51; Olympus, Tokyo, Japan). Image analysis was performed using ImageJ software^60^.

### Statistical analyses

Statistical analysis was performed using Prism 6 software (GraphPad Software, inc., La Jolla, CA, USA) and a Macintosh Pro personal computer (Apple Computer). To test the probability of significant differences between the untreated and treated samples, two-way analysis of variance (ANOVA) was used, and individual group comparisons were evaluated using Bonferroni’s test. The TUNEL staining data were analyzed via one-way analysis of variance (ANOVA), and individual group comparisons were evaluated using Bonferroni’s test. The differences were considered significant at *P* ≤ 0.05.

## Additional Information

**How to cite this article**: Locatelli, S. L. *et al.* Dual PI3K/ERK inhibition induces necroptotic cell death of Hodgkin Lymphoma cells through IER3 downregulation. *Sci. Rep.* **6**, 35745; doi: 10.1038/srep35745 (2016).

## Supplementary Material

Supplementary Information
