# Supplementary material for: Dual PI3K/ERK inhibition induces necroptotic cell death of Hodgkin Lymphoma cells through IER3 downregulation
Source: Sci Rep. 2016 Oct 21;6:35745. doi: 10.1038/srep35745 (PMC5073341; doi:10.1038/srep35745)
Supplement: Supplementary Information [file srep35745-s1.pdf]

Dual PI3K/ERK inhibition induces necroptotic cell death of Hodgkin Lymphoma cells through IER3 downregulation

*Silvia Laura Locatelli,[1] Giuseppa Careddu,[1] Giuliano Giuseppe Stirparo,[1] Luca Castagna,[1] Armando Santoro,[1,2] *Carmelo Carlo-Stella[1,3]

[1] Humanitas Cancer Center, Humanitas Clinical and Research Center, Rozzano, Italy; [2] Humanitas University, Rozzano, Italy; [3] Department of Medical Biotechnology and Translational Medicine, University of Milan, Milan, Italy

*Correspondence and requests for materials should be addressed to C. C.-S. (e-mail: carmelo.carlostella@cancercenter.humanitas.it) or S.L.L. (e-mail: silvia.locatelli@cancercenter.humanitas.it)

This supplemental material has been provided by the authors to provide readers with additional information regarding the study.

Supplemental Appendix

I. **Supplementary Figures and Legends**

II. **Supplementary Tables and Legends**

I. Supplementary Figures and Legends

Supplementary Fig. S1.

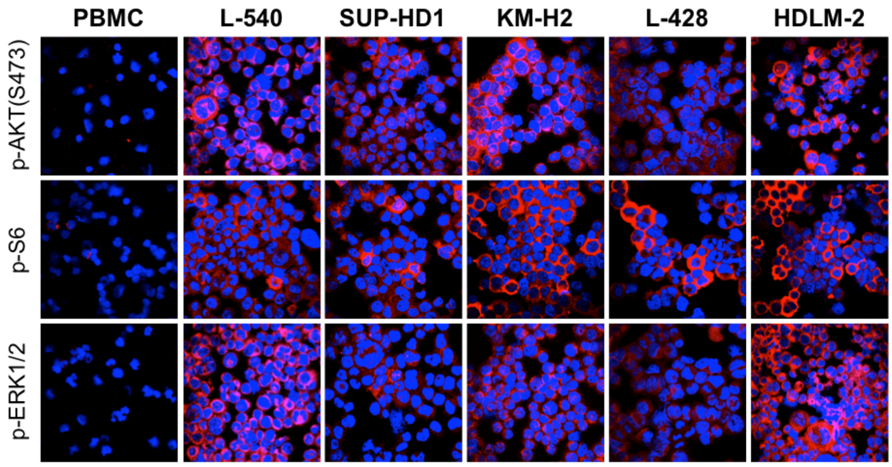

Supplementary Fig. S1. Cell expression profile of representative HL cell lines. Cell nuclei (*blue*) were detected using Hoechst. Protein expression (*red*) was detected using anti-pAKT, -pS6 and -pERK1/2 antibodies and Alexa Fluor 568-conjugated secondary antibodies. Peripheral mononuclear cells (PBMCs) were used as negative controls. Objective lens, original magnification: 1.0 NA oil objective, 60x.

Supplementary Fig. S2.

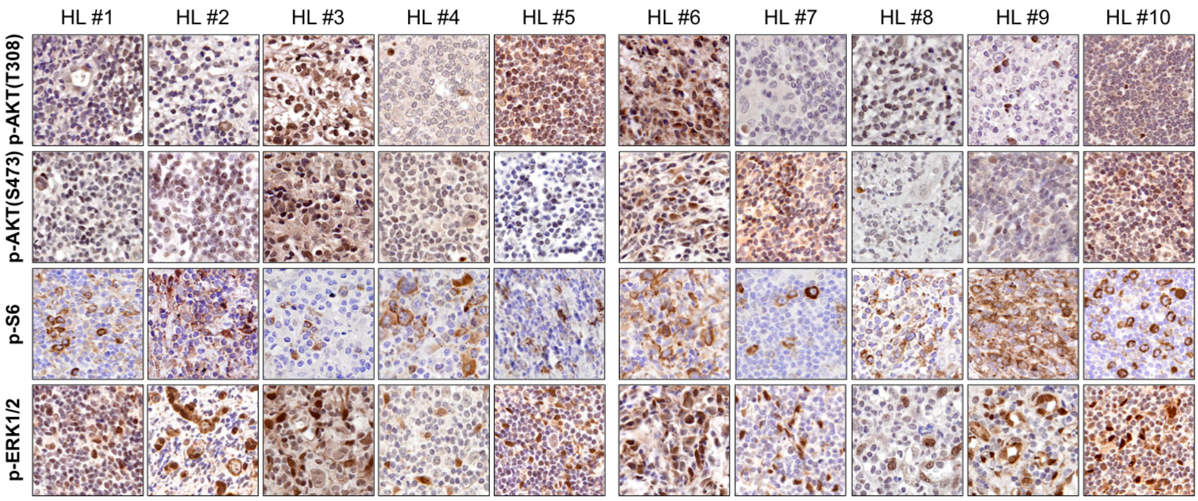

Supplementary Fig. S2. Tissue expression profiles of the PI3/AKT and MAPK/ERK pathways. Tissue expression profile of 10 representative cases of Hodgkin lymphoma are shown here (HL#1-5 chemosensitive patients; HL#6-10 chemoresistant patients). The choice of markers was based on the current literature on constitutively activate pathways in Hodgkin lymphoma (PI3/AKT and MAPK/ERK). Nuclear and cytoplasmic p-AKT, p-S6 and p-ERK provide evidence of activation of these pathways in the majority of HL cases evaluated in this study. Original magnification, 20x; zoom, 40x.

Supplementary Fig. S3.

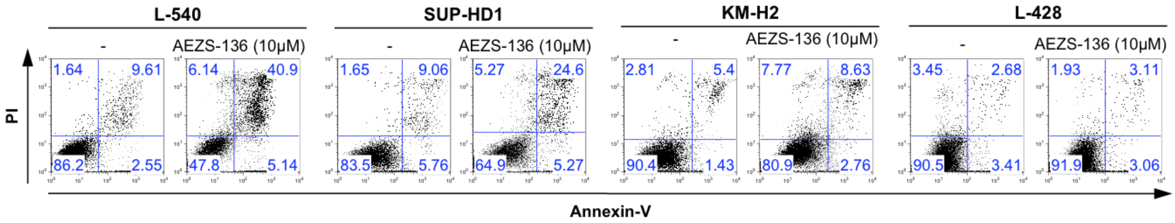

Supplementary Fig. S3. AEZS-136 exposure significantly increases caspase-independent HL cell death. Representative dot plots of cell death in vehicle-treated controls and AEZS-136-treated (10 μM) cell lines after 24 hours of exposure.

Supplementary Fig. S4.

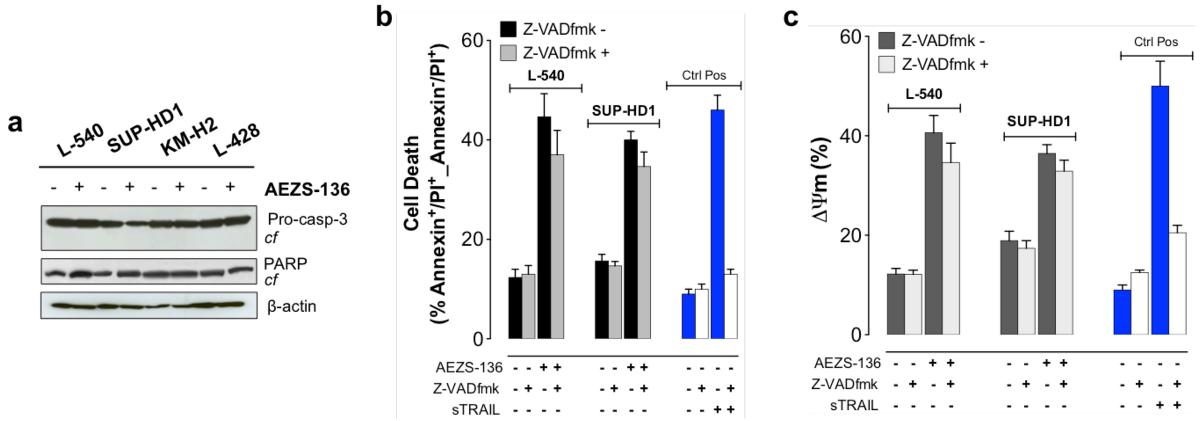

Supplementary Fig. S4. AEZS-136 exposure significantly increases caspase-independent HL cell death. (**a**) L-540, SUP-HD1, KM-H2, and L-428 cells were treated with 10 µM AEZS-136 or DMSO vehicle for 48 hours. Whole-cell lysates were obtained, and western blot analysis was performed to monitor caspase-3 cleavage/activation and PARP degradation. *Cf* indicates the cleaved fragments. The experiments were repeated twice with similar results. Representative blots are shown. L-540 and SUP-HD1 cells were pre-treated with 50 µM Z-VAD-FMK for 1 hour and then treated with 10 µM AEZS-136 or DMSO vehicle for 48 hours. Following this treatment, the cell death (**b**) and $\Delta\Psi_m$ (**c**) were assessed. KMS-11 cells exposed to 10 ng/ml sTRAIL were used as a positive control for the prevention of cell death and mitochondrial depolarization after Z-VAD-FMK treatment. The mean (± SEM) values correspond to three independent experiments.

Supplementary Fig. S5.

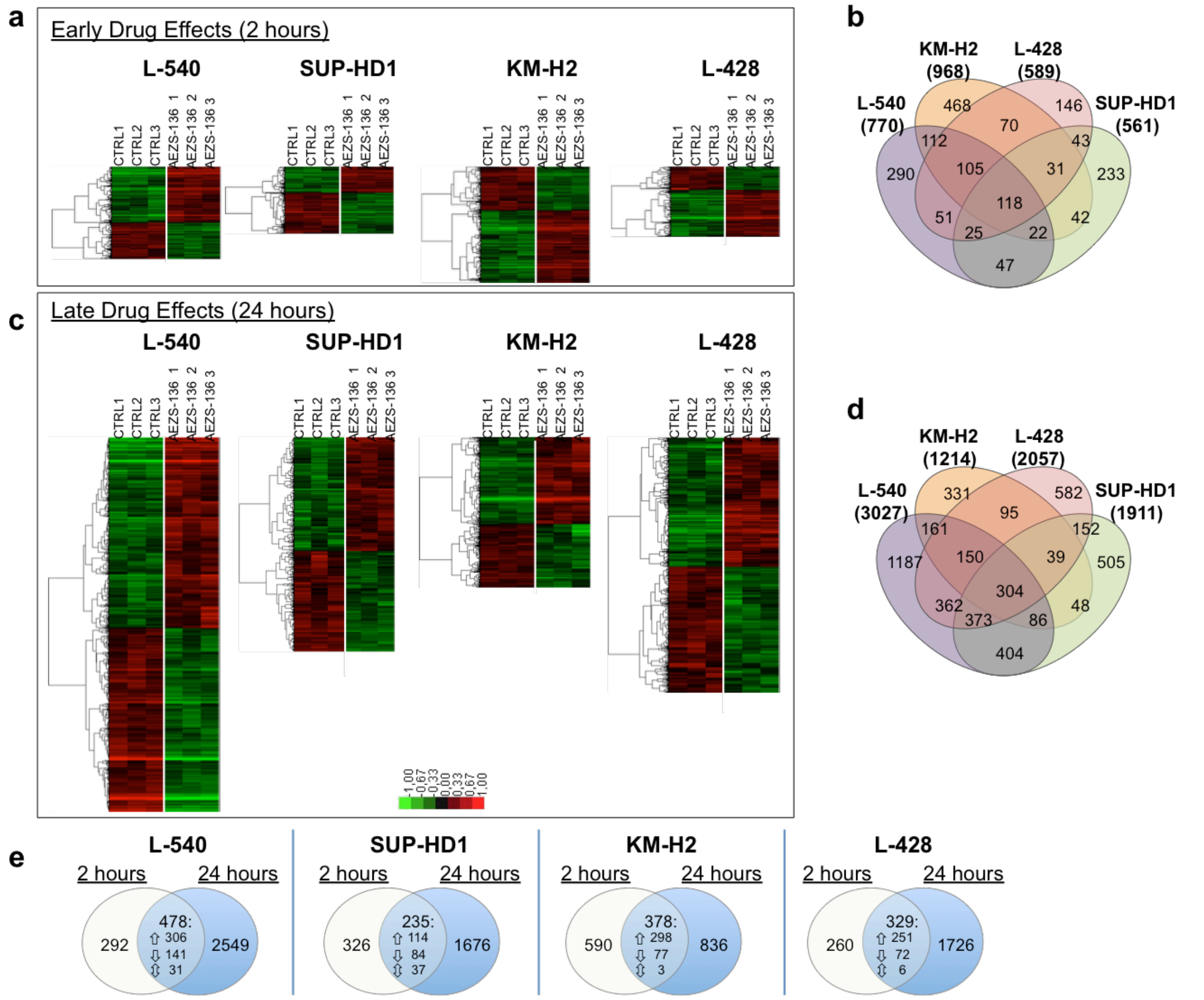

Supplementary Fig. S5. AEZS-136 modulates gene expression. (**a, c**) HL cells were exposed to 10 µM AEZS-136 or DMSO vehicle for 2 and 24 hours, and gene expression profiles were then analyzed. One-way hierarchical clustering of genes revealed significant modulation (adjusted P-value < 0.05) upon AEZS-136 treatment. The gene-wise median-centered normalized intensities (in log space) of the untreated cells (Ctrl_1-3) and the cells treated with AEZS-136 (AEZS-136_1-3) are shown. The heat map was clustered using centered correlation as the distance metric and using complete linkage clustering. (**b, d**) Venn diagram analysis of genes that were significantly modulated by AEZS-136 in the HL cells after 2 hours (**b**) and 24 hours (**d**). (**e**) Venn diagram analysis of genes that remained modulated by AEZS-136 between 2 hours and 24 hours in each cell line. Arrows indicated genes modulation at both 2 and 24 hours (⇧upregulated genes, ⇩ downregulated genes, ⇳ genes with opposite modulation).

Supplementary Fig. S6.

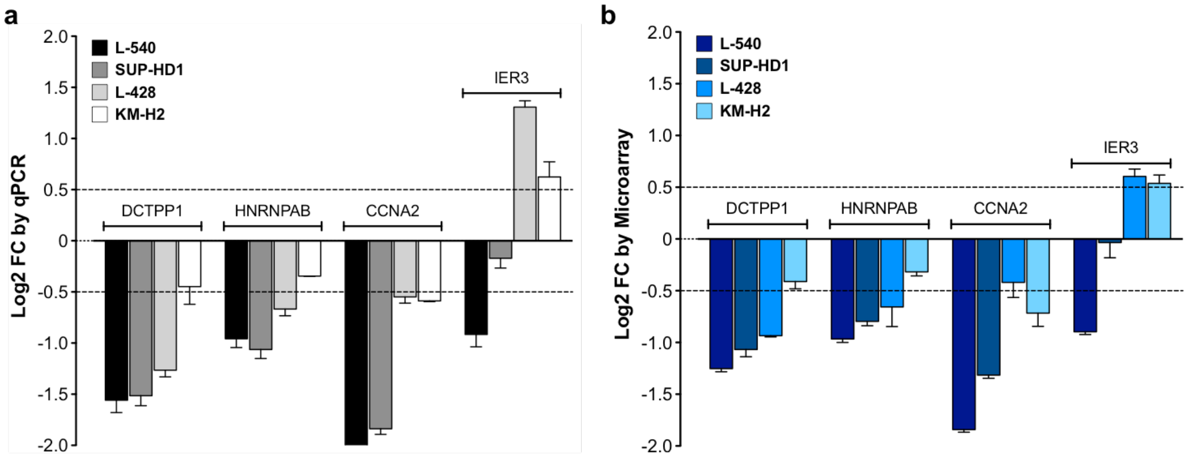

Supplementary Fig. S6. Validation of selected genes in HL cells treated with AEZS-136. The bar chart shows the gene expression patterns (presented as the fold-change relative to the control levels) of selected significantly differentially expressed genes (24 hours), calculated via real-time RT-PCR (**a**) and microarray analysis (**b**). All PCR data were normalized to the expression of β2-microglobulin as a housekeeping gene. Each histogram bar (± SEM) represents three independent experiments.

Supplementary Fig. S7.

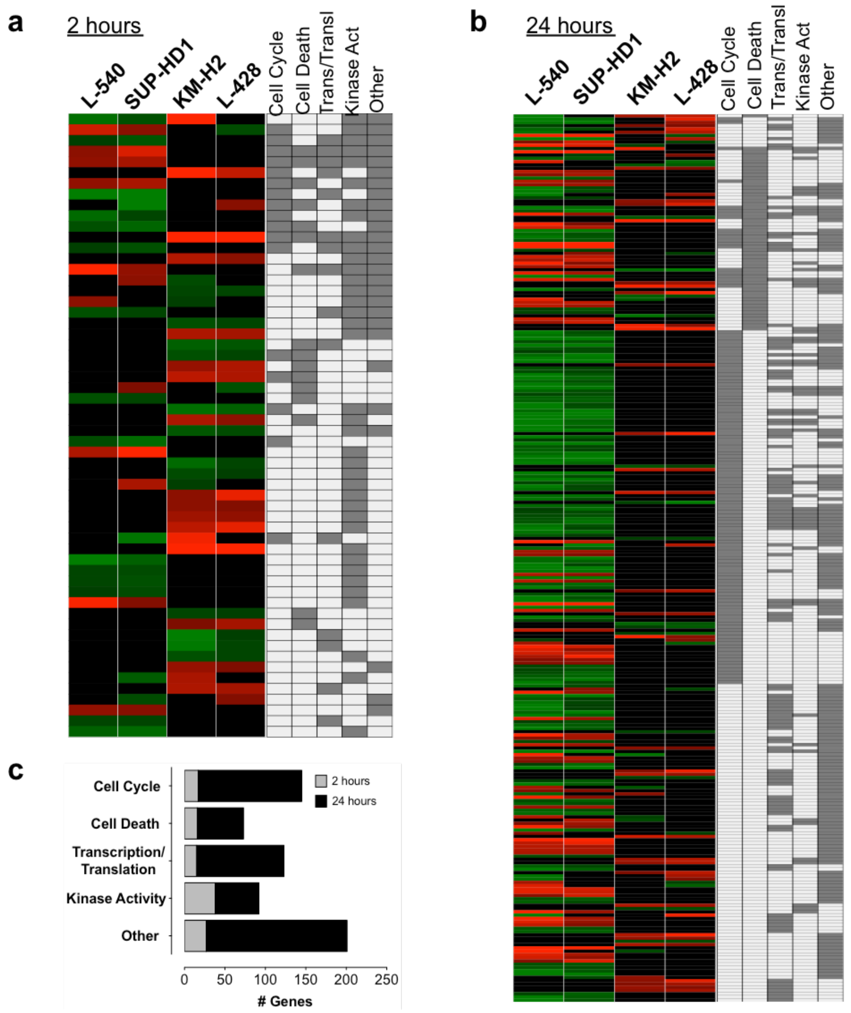

Supplementary Fig. S7. AEZS-136 modulated biological processes. (**a-b**) Heat map of filtered, differentially modulated genes listed in Supplemental table 1. Shown are the genes that were differentially expressed in at least two HL cell lines involved in significant biological processes (dark gray). (**c**) Histogram comparing the numbers of genes involved in the selected biological processes between 2 and 24 hours.

Supplementary Fig. S8.

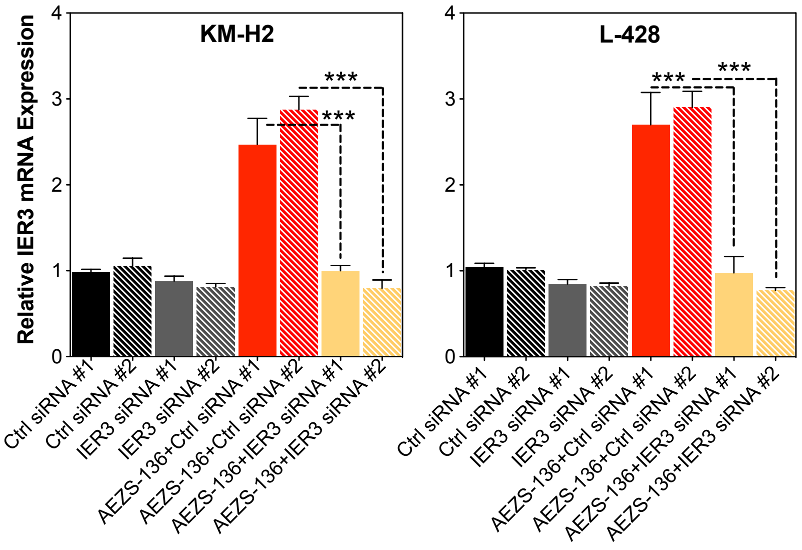

Supplementary Fig. S8. IER3 silencing. KM-H2 and L-428 cells were transfected with IER3-directed siRNA (100 nM) or control siRNA (100 nM) overnight. After transfection, the cells were treated with 10 µM AEZS-136 or DMSO vehicle. After 24 hours, the efficiency of IER3-directed siRNA inhibition was analyzed via real-time RT-PCR. *** $P \leq 0.0001$.

Supplementary Fig. S9.

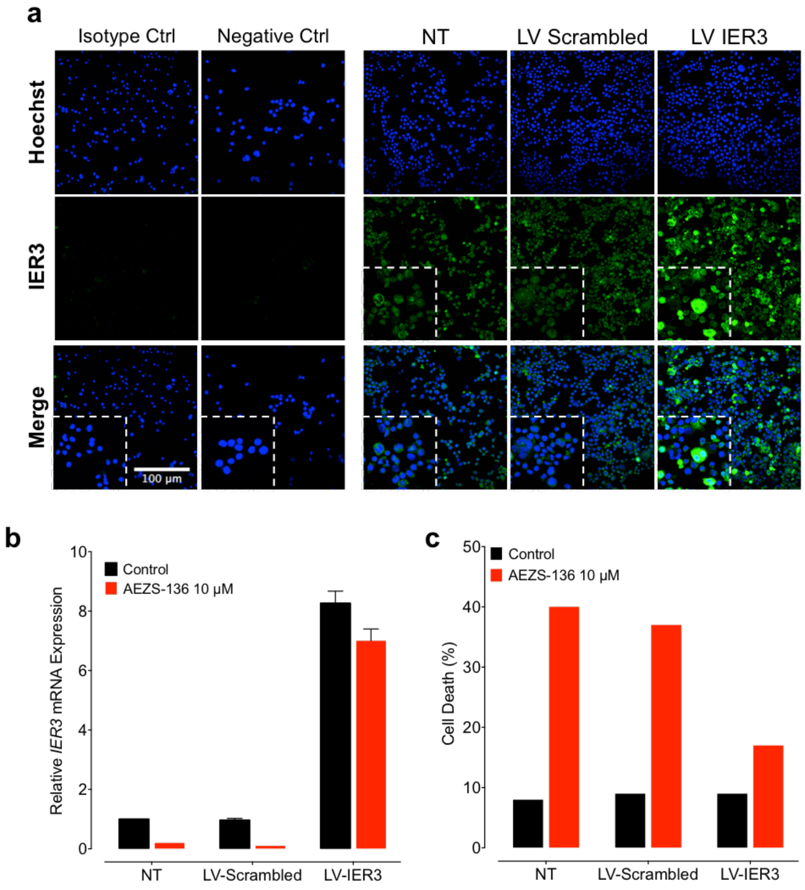

Supplementary Fig. S9. Monitoring IER3 lentiviral transduction efficiency of L-540 cells. Lentiviral vector-mediated *IER3* overexpression was visualized by (**a**) fluorescence microscopy six days after transfection, using an IER3-specific antibody and an Alexa Fluor 488-conjugated secondary antibody. Cell nuclei (*blue*) were detected using Hoechst dye. Original magnification, 20x; Insert, zoom 4x; scale bar, 100 µm. In the isotype control, the cells were incubated with a Goat IgG isotype control (Alexa Fluor 488) (antibodies-online GmbH, Germany, EU). In the negative control, the IER3 antibody was omitted, and the cells were incubated with a Alexa Fluor 488-conjugated anti-goat secondary antibody alone. NT, negative control group without transfected cells; LV Scrambled, negative control group with cells transfected with lentiviral vector alone; LV IER3, Study group (*IER3* overexpression group) with cells transfected with *IER3* lentiviral vector. (**b-c**) Seven days after transduction, cells were treated with AEZS-136 10 µM for 24 hrs, after that, (**b**) *IER3* mRNA expression and (**c**) cell death were assessed. Data are from one experiment representative of at least three independent experiments.

Supplementary Fig. S10.

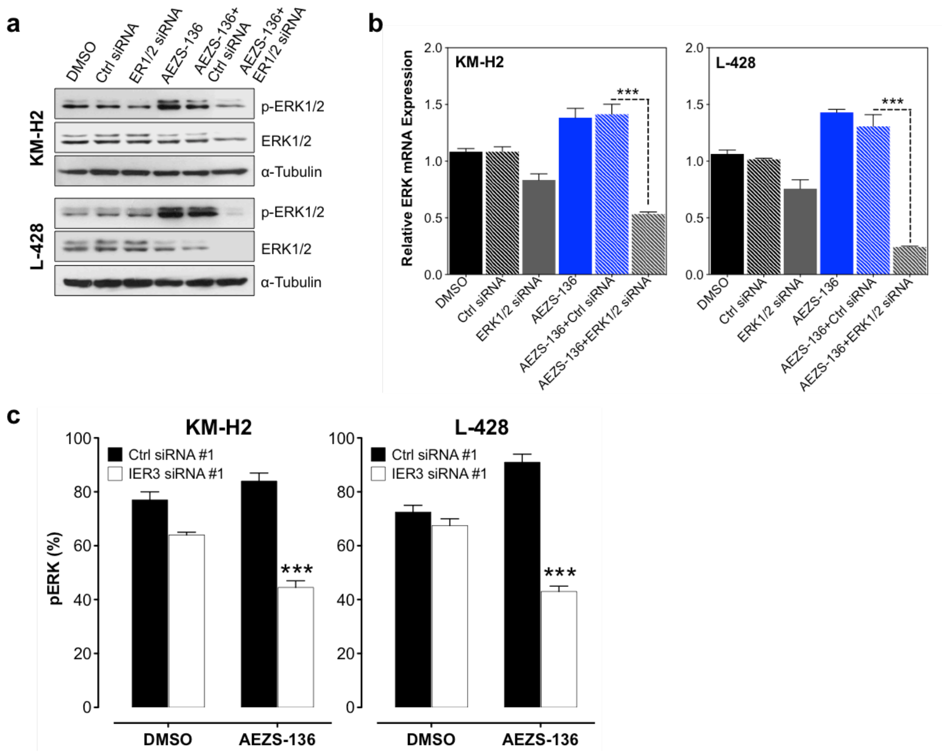

Supplementary Fig. S10. ERK1/2 expression after IER3 silencing. KM-H2 and L-428 cells were transfected with ERK1/2-directed siRNA (100 nM) or control siRNA (100 nM) overnight. After transfection, the cells were treated with 10 µM AEZS-136 or DMSO vehicle. After 48 hours, the efficiency of ERK1/2-directed siRNA inhibition on ERK1/2 was analyzed via western blot (**a**) and real-time RT-PCR (**b**). PCR data were normalized to the expression of β2-microglobulin as a housekeeping gene. (**c**) KM-H2 and L-428 cells were transfected with IER3-directed siRNA (100 nM) or control siRNA (100 nM) overnight. After transfection, the cells were treated with 10 µM AEZS-136 or DMSO vehicle. After 24 hours the expression of pERK1/2 was analyzed by flow cytometry. *** $P \leq 0.0001$ compared with control siRNA. Each histogram bar (± SEM) represents three independent experiments.

Supplementary Fig. S11.

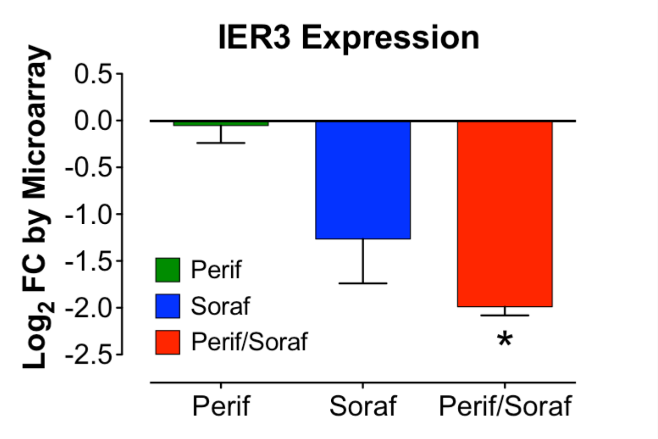

Supplementary Fig. S11. IER3 expression in L-540 cells treated with both perifosine and sorafenib. The bar chart shows the gene expression patterns (presented as the fold-changes relative to the control levels) of the *IER3* gene (24 hours) calculated via microarray analysis. Each histogram bar (± SEM) represents three independent experiments.

II. Supplementary Tables and Legends

Supplementary Table S1.

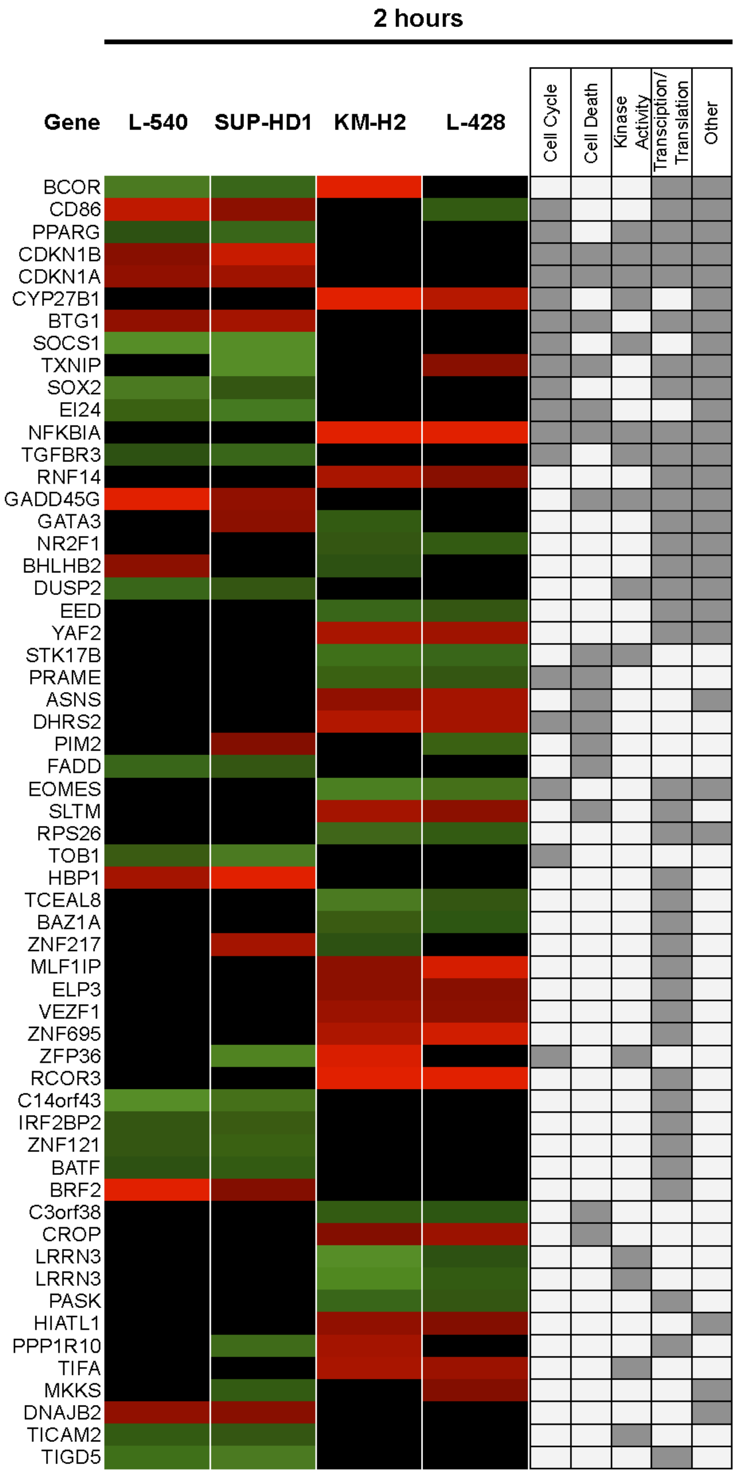

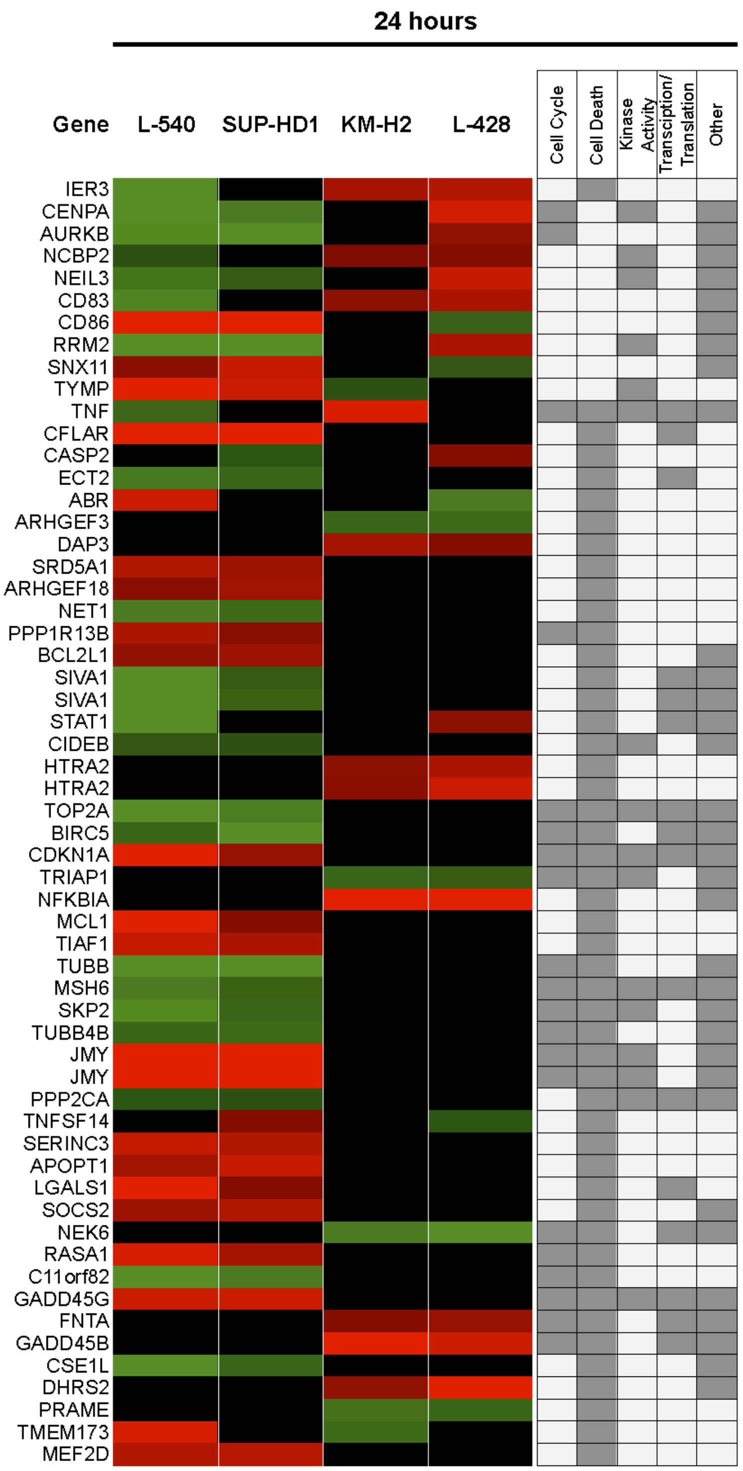
24 hours
Gene
L-540
SUP-HD1
KM-H2
L-428
Cell Cycle
Cell Death
Kinase Activity
Transciption/ Translation
Other
IER3
CENPA
AURKB
NCBP2
NEIL3
CD83
CD86
RRM2
SNX11
TYMP
TNF
CFLAR
CASP2
ECT2
ABR
ARHGEF3
DAP3
SRD5A1
ARHGEF18
NET1
PPP1R13B
BCL2L1
SIVA1
SIVA1
STAT1
CIDEB
HTRA2
HTRA2
TOP2A
BIRC5
CDKN1A
TRIAP1
NFKBIA
MCL1
TIAF1
TUBB
MSH6
SKP2
TUBB4B
JMY
JMY
PPP2CA
TNFSF14
SERINC3
APOPT1
LGALS1
SOCS2
NEK6
RASA1
C11orf82
GADD45G
FNTA
GADD45B
CSE1L
DHRS2
PRAME
TMEM173
MEF2D

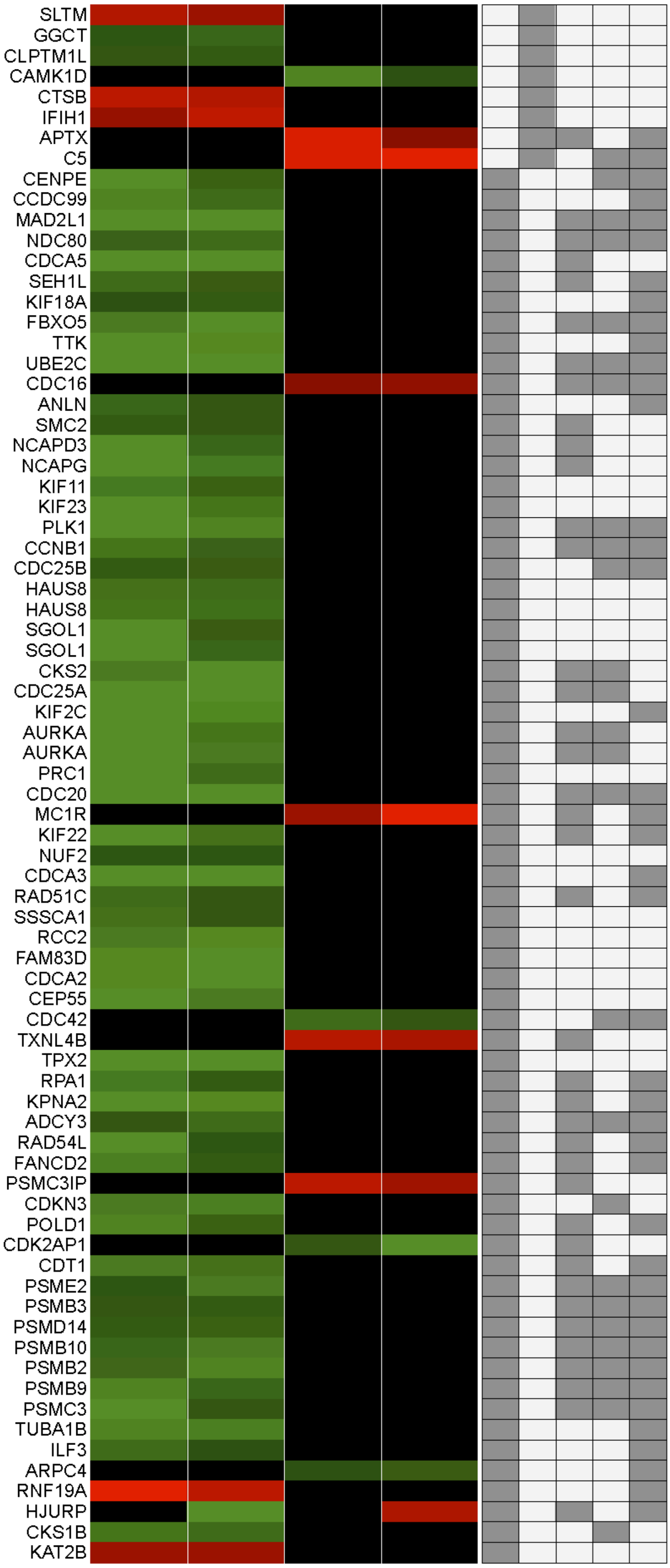
SLTM
GGCT
CLPTM1L
CAMK1D
CTSB
IFIH1
APTX
C5
CENPE
CCDC99
MAD2L1
NDC80
CDCA5
SEH1L
KIF18A
FBXO5
TTK
UBE2C
CDC16
ANLN
SMC2
NCAPD3
NCAPG
KIF11
KIF23
PLK1
CCNB1
CDC25B
HAUS8
HAUS8
SGOL1
SGOL1
CKS2
CDC25A
KIF2C
AURKA
AURKA
PRC1
CDC20
MC1R
KIF22
NUF2
CDCA3
RAD51C
SSSCA1
RCC2
FAM83D
CDCA2
CEP55
CDC42
TXNL4B
TPX2
RPA1
KPNA2
ADCY3
RAD54L
FANCD2
PSMC3IP
CDKN3
POLD1
CDK2AP1
CDT1
PSME2
PSMB3
PSMD14
PSMB10
PSMB2
PSMB9
PSMC3
TUBA1B
ILF3
ARPC4
RNF19A
HJURP
CKS1B
KAT2B

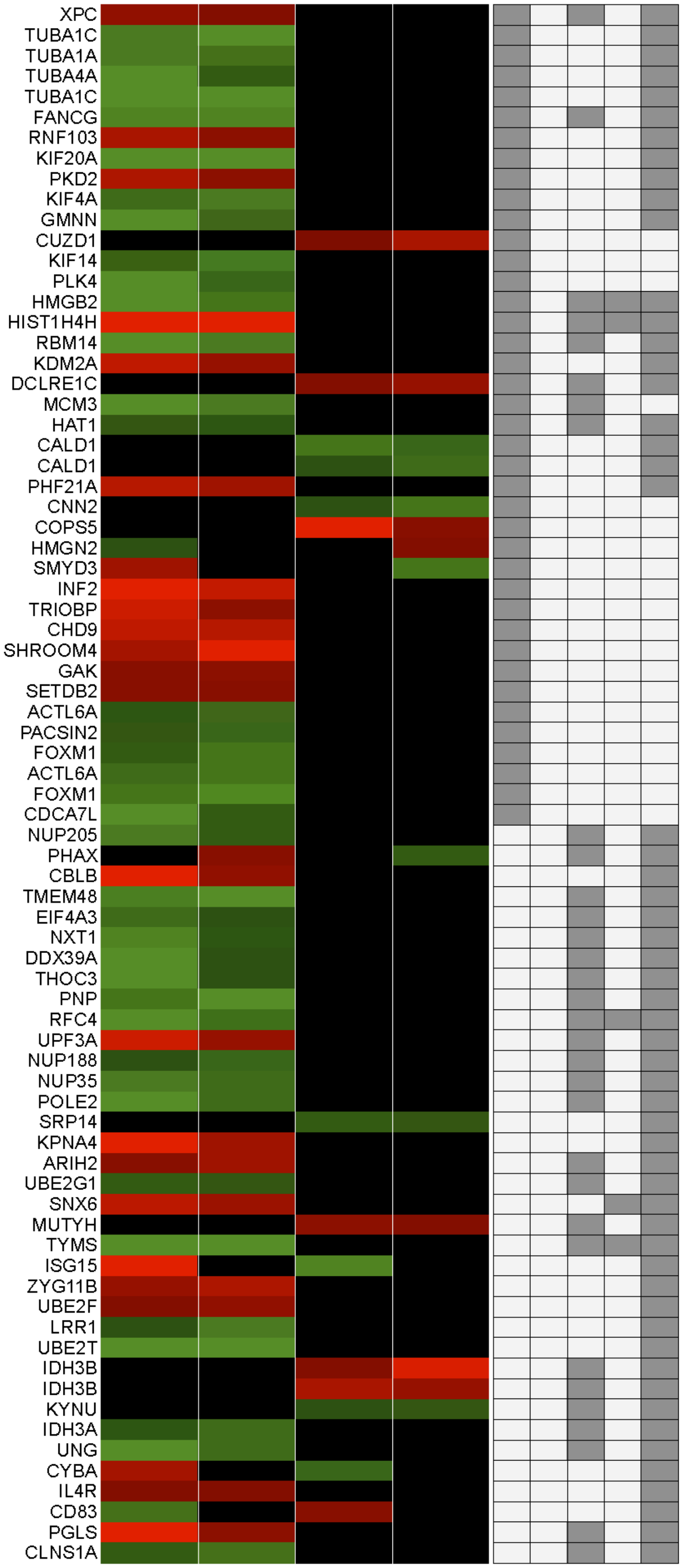
XPC
TUBA1C
TUBA1A
TUBA4A
TUBA1C
FANCG
RNF103
KIF20A
PKD2
KIF4A
GMNN
CUZD1
KIF14
PLK4
HMGB2
HIST1H4H
RBM14
KDM2A
DCLRE1C
MCM3
HAT1
CALD1
CALD1
PHF21A
CNN2
COPS5
HMGN2
SMYD3
INF2
TRIOBP
CHD9
SHROOM4
GAK
SETDB2
ACTL6A
PACSIN2
FOXM1
ACTL6A
FOXM1
CDCA7L
NUP205
PHAX
CBLB
TMEM48
EIF4A3
NXT1
DDX39A
THOC3
PNP
RFC4
UPF3A
NUP188
NUP35
POLE2
SRP14
KPNA4
ARIH2
UBE2G1
SNX6
MUTYH
TYMS
ISG15
ZYG11B
UBE2F
LRR1
UBE2T
IDH3B
IDH3B
KYNU
IDH3A
UNG
CYBA
IL4R
CD83
PGLS
CLNS1A

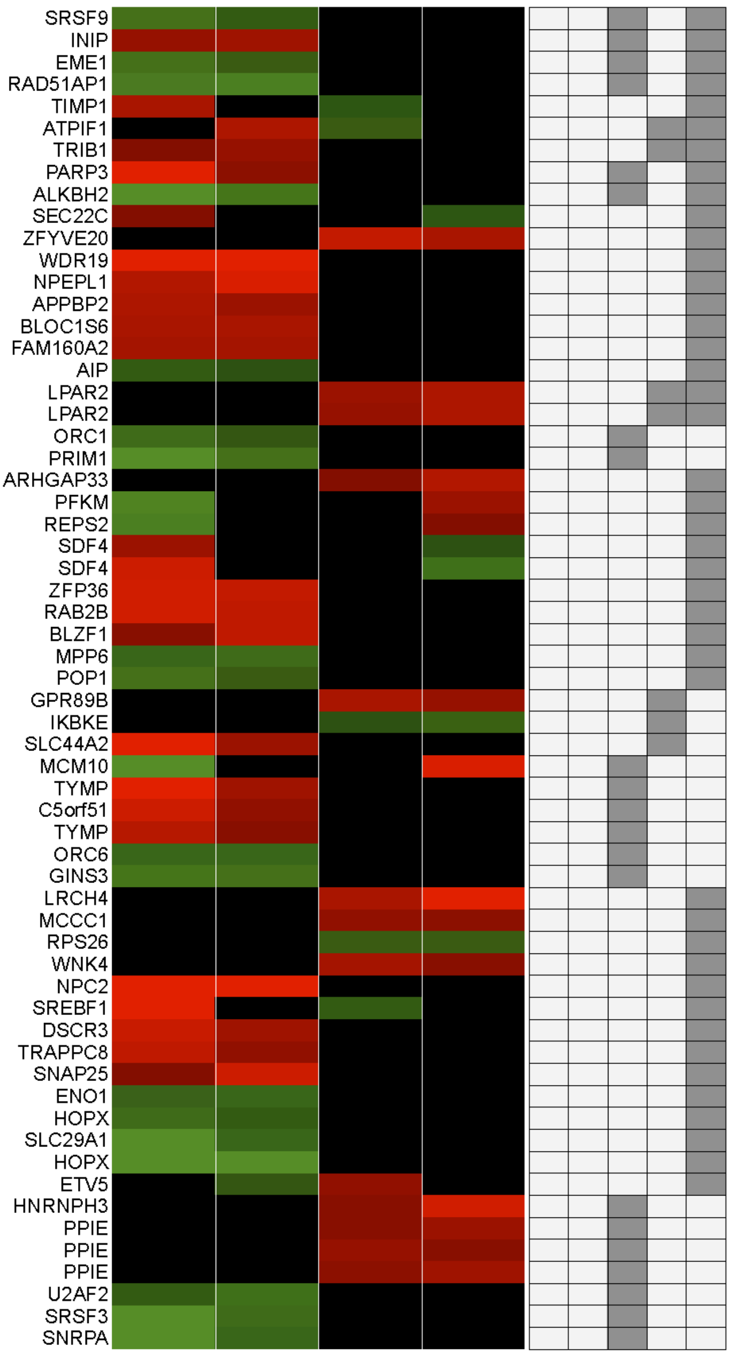

Supplementary Table S1. Filtered biological processes in HL cells 2 and 24 hours after AEZS-136 treatment. Heat map of filtered differentially modulated genes. Shown are genes expressed in at least two cell lines and with opposite modulation between AEZS-136-sensitive (L-540 and SUP-HD1) and AEZS-136-resistant (KM-H2 and L-428) cell lines, which are involved in significant (*P* < 0.05) biological processes (dark gray). The genes in non-significant processes were filtered out. Processes were grouped together according to their pertinence (cell cycle, cell death, signal transduction and kinase activity, transcription and translation).

Supplementary Table S2.

L-540/SUPHD-1				
Process	% Genes	Genes	Fold Enrichment	Benjamini
cell cycle	8.87	NM_052988, NM_182931, NM_000601, NM_017518, NM_182620, NM_006875, NM_018101, NM_002094, NM_002093, NM_006342, NM_002876, NM_006341, NM_002388, NM_080668, NM_015895, NM_005483, NM_016948, NM_032117, NM_145060, NM_000882, NM_145061, NM_182513, NM_002497, NM_013299, NM_007044, NM_032637, NM_006472, NM_001003786, NM_006325, NM_017668, NM_024094, NM_058216, NM_078468, NM_078467, NM_004358, NM_001032290, NM_004217, NM_004219, NM_001002800, NM_000249, NM_002795, NM_007317, NM_002794, NM_181042, NM_001007468, NM_000389, NM_005983, NM_004516, NM_030919, NM_002473, NM_018685, NM_002266, NM_031459, NM_007068, NM_002788, NM_024333, NM_002787, NM_013277, NM_016567, NM_012177, NM_000633, NM_001040694, NM_030900, NM_004343, NM_006888, NM_004701, NM_004336, NM_002691, NM_021145, NM_006600, NM_006086, NM_005789, NM_002791, NM_003318, NM_013282, NM_006704, NM_001042426, NM_004237, NM_145800, NM_003390, NM_016237, NM_012291, NM_015261, NM_145018, NM_198436, NM_198434, NM_018097, NM_001008938, NM_173083, NM_018492, NM_014814, NM_012197, NM_001083617, NM_001005413, NM_005192, NM_016038, NM_004725, NM_005591, NM_001621, NM_079421, NM_021953, NM_001789, NM_007111, NM_032485, NM_005590, NM_001786, NM_001761, NM_013345, NM_014303, NM_003884, NM_003883, NM_001760, NM_015276, NM_002263, NM_000234, NM_001790, NM_006624, NM_002358, NM_003503, NM_003504, NM_014708, NM_015169, NM_007221, NM_022809, NM_001037281, NM_025193, NM_152858, NM_032477, NM_006461, NM_015282, NM_031217, NM_181716, NM_006263, NM_024808, NM_013975, NM_024800, NM_006031, NM_006559, NM_181803, NM_002748, NM_006845, NM_002648, NM_194260, NM_025180, NM_022092, NM_181800, NM_018454, NM_002208, NM_001255, NM_014624, NM_001013437, NM_020536, NM_021205, NM_015714, NM_005915, NM_078487, NM_001010932, NM_000075, NM_178014, NM_170713, NM_020739, NM_015517, NM_006530, NM_020242, NM_004159, NM_000476, NM_015329, NM_000179, NM_152431, NM_001269, NM_006027, NM_012257, NM_001070, NM_002911, NM_198795, NM_002105, NM_001168, NM_016343, NM_017785, NM_001007793, NM_018171, NM_006400, NM_002417, NM_018715, NM_002710, NM_006503, NM_003707, NM_005667, NM_018571, NM_001667, NM_001012413, NM_000051, NM_015255, NM_000430, NM_014750, NM_002721, NM_152524, NM_003903, NM_138443, NM_203401, NM_001005753, NM_006396, NM_000057, NM_182776, NM_017760, NM_013374, NM_004036, NM_016195, NM_004450, NM_018193, NM_004856, NM_005563, NM_199413, NM_152513, NM_004127, NM_202003, NM_002945, NM_004064, NM_152405, NM_003183, NM_014762, NM_033028, NM_018410, NM_003579, NM_005255, NM_015374, NM_018944, NM_002808, NM_002809, NM_022346, NM_001826, NM_002804, NM_001827, NM_021732, NM_199246, NM_002800, NM_002801, NM_014865, NM_018140, NM_005445, NM_003920, NM_001018115, NM_005441, NM_002956, NM_012218, NM_024057, NM_139286, NM_030928, NM_004523, NM_003914, NM_002818, NM_005964, NM_001009944, NM_001813, NM_018136, NM_001950, NM_018031, NM_018131, NM_001011699, NM_002816, NM_002810, NM_012112, NM_002812, NM_004935, NM_031299, NM_198971, NM_004417, NM_001042550, NM_001031685, NM_005132, NM_145697, NM_005431, NM_001013836, NM_152132, NM_001098525, NM_138484, NM_005030, NM_182687, NM_199122, NM_004943, NM_152562, NM_003173, NM_006101, NM_031423, NM_005805, NM_017858, NM_033417, NM_000297, NM_031965, NM_031966	2.09	4.30E-33
cell cycle phase	5.57	NM_052988, NM_018454, NM_000601, NM_001255, NM_017518, NM_182620, NM_001013437, NM_006875, NM_018101, NM_021205, NM_002094, NM_078487, NM_001010932, NM_006342, NM_002876, NM_006341, NM_000075, NM_178014, NM_080668, NM_015517, NM_006530, NM_020242, NM_032117, NM_145060, NM_145061, NM_015329, NM_000179, NM_182513, NM_152431, NM_001269, NM_002497, NM_006027, NM_013299, NM_007044, NM_032637, NM_001070, NM_198795, NM_002105, NM_001168, NM_006325, NM_016343, NM_017785, NM_017668, NM_024094, NM_058216, NM_001007793, NM_006400, NM_078467, NM_004358, NM_002417, NM_018715, NM_004217, NM_004219, NM_001002800, NM_000249, NM_003707, NM_007317, NM_181042, NM_000389, NM_005983, NM_001012413, NM_000051, NM_004516, NM_030919, NM_015255, NM_000430, NM_002473, NM_014750, NM_002721, NM_002266, NM_152524, NM_018685, NM_138443, NM_003903, NM_007068, NM_024333, NM_203401, NM_012177, NM_000633, NM_001040694, NM_006396, NM_000057, NM_030900, NM_017760, NM_004036, NM_004701, NM_004336, NM_002691, NM_006600, NM_006086, NM_016195, NM_005563, NM_004856, NM_003318, NM_006704, NM_199413, NM_152513, NM_004237, NM_002945, NM_003390, NM_004064, NM_016237, NM_012291, NM_003183, NM_015261, NM_003579, NM_198436, NM_015374, NM_198434, NM_018944, NM_018097, NM_001008938, NM_022346, NM_018492, NM_001827, NM_199246, NM_001005413, NM_014865, NM_005192, NM_005445, NM_003920, NM_004725, NM_001018115, NM_002956, NM_005591, NM_012218, NM_079421, NM_001789, NM_024057, NM_001786, NM_005590, NM_139286, NM_001761, NM_014303, NM_013345, NM_003883, NM_004523, NM_002263, NM_003914, NM_001813, NM_001790, NM_018136, NM_001950, NM_018131, NM_001011699, NM_012112, NM_002358, NM_003503, NM_014708, NM_031299, NM_015169, NM_198971, NM_001042550, NM_005132, NM_007221, NM_022809, NM_145697, NM_025193, NM_005431, NM_001013836, NM_006461, NM_015282, NM_181716, NM_031217, NM_138484, NM_024808, NM_005030, NM_182687, NM_013975, NM_199122, NM_004943, NM_006031, NM_006559, NM_152562, NM_181803, NM_006845, NM_006101, NM_194260, NM_002648, NM_025180, NM_031423, NM_017858, NM_033417, NM_031966, NM_181800	2.47	1.97E-29
cell cycle process	6.96	NM_052988, NM_182931, NM_000601, NM_017518, NM_182620, NM_006875, NM_018101, NM_002094, NM_002093, NM_006342, NM_002876, NM_006341, NM_080668, NM_032117, NM_145060, NM_000882, NM_145061, NM_182513, NM_002497, NM_013299, NM_007044, NM_032637, NM_006325, NM_017668, NM_024094, NM_058216, NM_078467, NM_004358, NM_004217, NM_004219, NM_001002800, NM_000249, NM_002795, NM_002794, NM_007317, NM_181042, NM_000389, NM_005983, NM_030919, NM_004516, NM_002473, NM_018685, NM_002266, NM_031459, NM_007068, NM_024333, NM_002788, NM_013277, NM_002787, NM_012177, NM_000633, NM_001040694, NM_030900, NM_004343, NM_004701, NM_004336, NM_002691, NM_006086, NM_006600, NM_005789, NM_002791, NM_003318, NM_001042426, NM_006704, NM_004237, NM_003390, NM_016237, NM_012291, NM_015261, NM_145018, NM_198436, NM_198434, NM_018097, NM_001008938, NM_018492, NM_014814, NM_001005413, NM_005192, NM_016038, NM_004725, NM_005591, NM_079421, NM_001789, NM_005590, NM_001786, NM_001761, NM_013345, NM_014303, NM_003884, NM_003883, NM_002263, NM_001790, NM_002358, NM_003503, NM_014708, NM_015169, NM_007221, NM_022809, NM_025193, NM_006461, NM_015282, NM_181716, NM_031217, NM_006263, NM_024808, NM_013975, NM_006031, NM_006559, NM_181803, NM_006845, NM_194260, NM_002648, NM_025180, NM_181800, NM_018454, NM_001255, NM_001013437, NM_020536, NM_021205, NM_078487, NM_001010932, NM_000075, NM_178014, NM_170713, NM_015517, NM_006530, NM_020242, NM_004159, NM_000476, NM_015329, NM_000179, NM_152431, NM_001269, NM_012257, NM_006027, NM_001070, NM_198795, NM_002105, NM_001168, NM_016343, NM_017785, NM_001007793, NM_006400, NM_002417, NM_018715, NM_006503, NM_003707, NM_001012413, NM_000051, NM_015255, NM_000430, NM_002721, NM_014750, NM_152524, NM_003903, NM_138443, NM_203401, NM_006396, NM_000057, NM_017760, NM_004036, NM_016195, NM_004856, NM_005563, NM_199413, NM_152513, NM_002945, NM_004064, NM_152405, NM_003183, NM_014762, NM_033028, NM_003579, NM_015374, NM_018944, NM_002808, NM_002809, NM_022346, NM_002804, NM_001827, NM_199246, NM_002800, NM_014865, NM_002801, NM_018140, NM_005445, NM_003920, NM_001018115, NM_002956, NM_012218, NM_024057, NM_139286, NM_004523, NM_003914, NM_002818, NM_001009944, NM_005964, NM_001813, NM_018136, NM_001950, NM_018031, NM_018131, NM_001011699, NM_002816, NM_002810, NM_012112, NM_002812, NM_031299, NM_198971, NM_001042550, NM_005132, NM_145697, NM_005431, NM_001013836, NM_152132, NM_138484, NM_005030, NM_182687, NM_199122, NM_004943, NM_152562, NM_006101, NM_005805, NM_031423, NM_017858, NM_033417, NM_000297, NM_031966	2.26	1.55E-30
cell death	5.11	NM_002305, NM_000165, NM_024900, NM_152778, NM_001066, NM_006281, NM_000600, NM_015331, NM_007051, NM_018844, NM_006875, NM_014959, NM_002094, NM_000417, NM_005605, NM_020247, NM_001540, NM_016561, NM_001024465, NM_024091, NM_145341, NM_198057, NM_144781, NM_003842, NM_001168, NM_021709, NM_014634, NM_018319, NM_021999, NM_031301, NM_001047160, NM_133328, NM_001013739, NM_016454, NM_002513, NM_005920, NM_016553, NM_024544, NM_015318, NM_003028, NM_016077, NM_001067, NM_015316, NM_021076, NM_001008540, NM_025137, NM_007315, NM_000247, NM_032992, NM_016640, NM_014341, NM_004330, NM_138578, NM_003467, NM_022157, NM_021127, NM_000595, NM_002727, NM_000594, NM_000391, NM_000051, NM_030782, NM_006698, NM_030660, NM_004708, NM_182962, NM_032983, NM_006290, NM_001092, NM_001097577, NM_000633, NM_014430, NM_144599, NM_152240, NM_032989, NM_005658, NM_030900, NM_013374, NM_016424, NM_001033030, NM_016009, NM_005789, NM_002133, NM_003311, NM_181598, NM_006705, NM_024755, NM_018098, NM_001037333, NM_004064, NM_201559, NM_001008213, NM_012291, NM_002157, NM_002156, NM_014762, NM_003879, NM_021960, NM_033027, NM_033339, NM_145018, NM_015367, NM_024051, NM_003820, NM_024948, NM_004740, NM_003680, NM_006570, NM_003789, NM_001621, NM_133171, NM_079421, NM_145813, NM_201430, NM_012215, NM_013258, NM_133369, NM_003810, NM_005041, NM_012486, NM_006427, NM_024065, NM_001226, NM_197962, NM_004935, NM_001375, NM_019887, NM_002654, NM_001031685, NM_198120, NM_001013843, NM_001040668, NM_182471, NM_004089, NM_032477, NM_020919, NM_003897, NM_003374, NM_012329, NM_001098525, NM_033294, NM_014281, NM_153719, NM_201397, NM_002222, NM_003946, NM_003805, NM_003804, NM_199122, NM_001183, NM_005428, NM_181676, NM_021830, NM_003768, NM_003217, NM_004960, NM_198282, NM_139266, NM_031365, NM_001316, NM_002583, NM_018941, NM_005118, NM_012138	1.30	0.012160412
cellular macromolecular complex assembly	3.46	NM_021059, NM_002014, NM_000801, NM_003534, NM_006109, NM_018087, NM_003531, NM_178014, NM_003537, NM_003535, NM_020243, NM_021062, NM_005483, NM_006009, NM_006938, NM_015135, NM_002495, NM_001079866, NM_006026, NM_001070, NM_002105, NM_015721, NM_002106, NM_000377, NM_016343, NM_005348, NM_054014, NM_006925, NM_177925, NM_003543, NM_003542, NM_014165, NM_003545, NM_003544, NM_016338, NM_005138, NM_015629, NM_003548, NM_004719, NM_000249, NM_005359, NM_016437, NM_001667, NM_003516, NM_003514, NM_003513, NM_032704, NM_000101, NM_003096, NM_003512, NM_003095, NM_018685, NM_003518, NM_003517, NM_015380, NM_001097577, NM_012177, NM_003011, NM_006000, NM_001013699, NM_001039619, NM_001034077, NM_002129, NM_004343, NM_001040874, NM_003524, NM_003521, NM_006087, NM_006086, NM_003522, NM_006088, NM_006082, NM_001293, NM_003529, NM_001042426, NM_004069, NM_006831, NM_024658, NM_003201, NM_002156, NM_014260, NM_018410, NM_001017980, NM_000024, NM_024102, NM_080593, NM_015465, NM_005441, NM_032177, NM_001042581, NM_024120, NM_001017990, NM_001042588, NM_001017991, NM_001813, NM_005969, NM_002154, NM_004935, NM_016101, NM_024831, NM_030752, NM_007362, NM_199069, NM_004492, NM_138609, NM_181716, NM_001017963, NM_001005464, NM_005324, NM_001123375, NM_004893, NM_007166, NM_003172, NM_003769, NM_005320, NM_005322, NM_001078166, NM_001316, NM_005318, NM_005319, NM_018154, NM_198576, NM_004552, NM_004818	1.99	1.58E-10
cellular macromolecular complex subunit organization	3.79	NM_021059, NM_002014, NM_000801, NM_001013437, NM_003534, NM_006109, NM_018087, NM_003531, NM_178014, NM_003537, NM_003535, NM_020243, NM_021062, NM_005483, NM_001545, NM_006009, NM_006938, NM_015135, NM_002495, NM_001079866, NM_006026, NM_001070, NM_002105, NM_015721, NM_002106, NM_000377, NM_016343, NM_005348, NM_054014, NM_003543, NM_006925, NM_177925, NM_003542, NM_014165, NM_003545, NM_003544, NM_016338, NM_005138, NM_015629, NM_003548, NM_004719, NM_000249, NM_005359, NM_016437, NM_001667, NM_003516, NM_003514, NM_003513, NM_032704, NM_000101, NM_003096, NM_003512, NM_003095, NM_022909, NM_018685, NM_003518, NM_003517, NM_015380, NM_203401, NM_001097577, NM_012177, NM_003011, NM_006000, NM_001013699, NM_001039619, NM_001034077, NM_019041, NM_004343, NM_002129, NM_001040874, NM_003524, NM_003521, NM_006087, NM_006086, NM_199177, NM_003522, NM_006088, NM_005563, NM_006082, NM_001293, NM_003529, NM_001042426, NM_004069, NM_006831, NM_024658, NM_003201, NM_002156, NM_014260, NM_018410, NM_001017980, NM_000024, NM_024102, NM_080593, NM_015465, NM_005441, NM_032177, NM_001042581, NM_024120, NM_001017990, NM_001042588, NM_001017991, NM_001813, NM_005969, NM_003594, NM_002154, NM_004935, NM_016101, NM_024831, NM_030752, NM_007362, NM_199069, NM_004492, NM_138609, NM_031217, NM_181716, NM_018230, NM_001017963, NM_001005464, NM_005324, NM_001123375, NM_004893, NM_007166, NM_003172, NM_003769, NM_006845, NM_005320, NM_005322, NM_001078166, NM_001316, NM_005318, NM_005319, NM_018154, NM_198576, NM_004552, NM_004818	1.95	8.37E-11
cellular response to stress	5.21	NM_003350, NM_003146, NM_004579, NM_017518, NM_001923, NM_006666, NM_001014436, NM_001033002, NM_002907, NM_023015, NM_002093, NM_002876, NM_015517, NM_005483, NM_000477, NM_005484, NM_001042618, NM_006479, NM_199420, NM_005485, NM_001024465, NM_000179, NM_006020, NM_006027, NM_001071, NM_032444, NM_002911, NM_002105, NM_016448, NM_006328, NM_002916, NM_018319, NM_001003931, NM_001039847, NM_080876, NM_001001655, NM_058216, NM_031482, NM_001040439, NM_078468, NM_001527, NM_078467, NM_022166, NM_181573, NM_152633, NM_181471, NM_001042697, NM_024544, NM_181696, NM_001067, NM_004219, NM_001042600, NM_182640, NM_000249, NM_000247, NM_001990, NM_007317, NM_000389, NM_021141, NM_018571, NM_001080449, NM_030811, NM_002896, NM_080649, NM_002528, NM_198948, NM_001127218, NM_000594, NM_000051, NM_006793, NM_030660, NM_006515, NM_018975, NM_016567, NM_178150, NM_004793, NM_001357, NM_000633, NM_001605, NM_001031716, NM_014430, NM_000057, NM_182776, NM_006297, NM_152240, NM_002129, NM_006304, NM_001144, NM_004454, NM_004029, NM_002691, NM_001204, NM_080911, NM_002692, NM_002693, NM_002133, NM_018193, NM_013282, NM_016395, NM_181738, NM_004127, NM_016290, NM_004237, NM_006705, NM_198336, NM_002947, NM_002946, NM_002945, NM_201559, NM_152405, NM_014063, NM_021821, NM_003392, NM_003579, NM_001752, NM_014311, NM_198954, NM_001618, NM_003925, NM_001083617, NM_014967, NM_139076, NM_000123, NM_012094, NM_005445, NM_003920, NM_001018115, NM_005441, NM_004628, NM_004629, NM_005591, NM_181652, NM_001006622, NM_079421, NM_145813, NM_005590, NM_001786, NM_001003686, NM_000234, NM_001005291, NM_014502, NM_197962, NM_013451, NM_005395, NM_198971, NM_001893, NM_005431, NM_025195, NM_052966, NM_024068, NM_005531, NM_173627, NM_201397, NM_014481, NM_013975, NM_145043, NM_152463, NM_144729, NM_003804, NM_024800, NM_015920, NM_021218, NM_001641, NM_007370, NM_003362, NM_025180, NM_007027, NM_014071, NM_018445, NM_018248, NM_017858, NM_012138, NM_001029835	1.69	1.12E-09
chromosome organization	4.49	NM_012461, NM_182931, NM_021059, NM_024900, NM_018454, NM_006666, NM_001013437, NM_020536, NM_018101, NM_003534, NM_006109, NM_003531, NM_002876, NM_004964, NM_003537, NM_080668, NM_003535, NM_005483, NM_021062, NM_006530, NM_018081, NM_015329, NM_015013, NM_000179, NM_002497, NM_006473, NM_006026, NM_019088, NM_002105, NM_002106, NM_006328, NM_016343, NM_017785, NM_024094, NM_003543, NM_177925, NM_001039847, NM_003542, NM_058216, NM_024165, NM_003545, NM_001007793, NM_003544, NM_001527, NM_003548, NM_003642, NM_020920, NM_001067, NM_004219, NM_001002800, NM_021078, NM_003707, NM_001990, NM_181042, NM_001007468, NM_019071, NM_021141, NM_003516, NM_002896, NM_003514, NM_031915, NM_002893, NM_003513, NM_003512, NM_022909, NM_014750, NM_152524, NM_003518, NM_003517, NM_006515, NM_002931, NM_018975, NM_177989, NM_003011, NM_001013699, NM_000057, NM_001039619, NM_001034077, NM_017760, NM_002129, NM_001040874, NM_003524, NM_003521, NM_016621, NM_003522, NM_017553, NM_003529, NM_001042426, NM_183359, NM_016290, NM_001363, NM_002945, NM_152299, NM_002634, NM_005644, NM_012291, NM_015261, NM_012308, NM_018410, NM_003579, NM_015265, NM_001618, NM_022346, NM_001018077, NM_005056, NM_018405, NM_139076, NM_001005413, NM_014865, NM_003074, NM_005445, NM_004301, NM_080593, NM_004725, NM_001018115, NM_005441, NM_005591, NM_024644, NM_145813, NM_001017990, NM_005590, NM_003884, NM_003883, NM_001017991, NM_002263, NM_015276, NM_016374, NM_025134, NM_005189, NM_001813, NM_005969, NM_003609, NM_001379, NM_002358, NM_012117, NM_015169, NM_001042550, NM_005132, NM_022743, NM_181716, NM_138609, NM_031217, NM_001005464, NM_005324, NM_001123375, NM_004893, NM_003173, NM_006101, NM_005320, NM_005517, NM_005322, NM_005318, NM_005319, NM_018154, NM_003168, NM_031965, NM_015695	1.69	2.30E-08
death	5.11	NM_002305, NM_000165, NM_024900, NM_152778, NM_001066, NM_006281, NM_000600, NM_015331, NM_007051, NM_018844, NM_006875, NM_014959, NM_002094, NM_000417, NM_005605, NM_020247, NM_001540, NM_016561, NM_001024465, NM_024091, NM_145341, NM_198057, NM_144781, NM_003842, NM_001168, NM_021709, NM_014634, NM_018319, NM_021999, NM_031301, NM_001047160, NM_133328, NM_001013739, NM_016454, NM_002513, NM_005920, NM_016553, NM_024544, NM_015318, NM_003028, NM_016077, NM_001067, NM_015316, NM_021076, NM_001008540, NM_025137, NM_007315, NM_000247, NM_032992, NM_016640, NM_014341, NM_004330, NM_138578, NM_003467, NM_022157, NM_021127, NM_000595, NM_002727, NM_000594, NM_000391, NM_000051, NM_030782, NM_006698, NM_030660, NM_004708, NM_182962, NM_032983, NM_006290, NM_001092, NM_001097577, NM_000633, NM_014430, NM_144599, NM_152240, NM_032989, NM_005658, NM_030900, NM_013374, NM_016424, NM_001033030, NM_016009, NM_005789, NM_002133, NM_003311, NM_181598, NM_006705, NM_024755, NM_018098, NM_001037333, NM_004064, NM_201559, NM_001008213, NM_012291, NM_002157, NM_002156, NM_014762, NM_003879, NM_021960, NM_033027, NM_033339, NM_145018, NM_015367, NM_024051, NM_003820, NM_024948, NM_004740, NM_003680, NM_006570, NM_003789, NM_001621, NM_133171, NM_079421, NM_145813, NM_201430, NM_012215, NM_013258, NM_133369, NM_003810, NM_005041, NM_012486, NM_006427, NM_024065, NM_001226, NM_197962, NM_004935, NM_001375, NM_019887, NM_002654, NM_001031685, NM_198120, NM_001013843, NM_001040668, NM_182471, NM_004089, NM_032477, NM_020919, NM_003897, NM_003374, NM_012329, NM_001098525, NM_033294, NM_014281, NM_153719, NM_201397, NM_002222, NM_003946, NM_003805, NM_003804, NM_199122, NM_001183, NM_005428, NM_181676, NM_021830, NM_003768, NM_003217, NM_004960, NM_198282, NM_139266, NM_031365, NM_001316, NM_002583, NM_018941, NM_005118, NM_012138	1.29	0.01644353

DNA metabolic process	5.67	NM_182931, NM_012461, NM_003350, NM_003146, NM_018518, NM_152308, NM_017518, NM_001923, NM_006666, NM_173518, NM_018845, NM_002201, NM_001033002, NM_002907, NM_023015, NM_005915, NM_002876, NM_002388, NM_006190, NM_015517, NM_017660, NM_005483, NM_006739, NM_005484, NM_018081, NM_002501, NM_001042618, NM_006479, NM_032117, NM_005485, NM_199420, NM_004774, NM_001024465, NM_000179, NM_004153, NM_152431, NM_006020, NM_006027, NM_001071, NM_032444, NM_002911, NM_198795, NM_002105, NM_016448, NM_006328, NM_006325, NM_016343, NM_002916, NM_001003931, NM_018319, NM_024094, NM_001001655, NM_058216, NM_078468, NM_181573, NM_152633, NM_181471, NM_001042697, NM_003143, NM_001067, NM_004219, NM_003707, NM_000249, NM_001990, NM_007317, NM_001007468, NM_021130, NM_021141, NM_001113755, NM_001080449, NM_001113756, NM_002896, NM_080649, NM_002893, NM_002528, NM_021137, NM_198948, NM_001127218, NM_000051, NM_002897, NM_002824, NM_002266, NM_003860, NM_001009934, NM_007068, NM_030660, NM_006515, NM_018975, NM_016567, NM_178150, NM_003011, NM_004793, NM_006397, NM_001031716, NM_000057, NM_182776, NM_003258, NM_006297, NM_002129, NM_006304, NM_178863, NM_002691, NM_080911, NM_002692, NM_002693, NM_018193, NM_001552, NM_013282, NM_017443, NM_016290, NM_001363, NM_004237, NM_006705, NM_002947, NM_002946, NM_002634, NM_002945, NM_152405, NM_002156, NM_003201, NM_000946, NM_003579, NM_014311, NM_198954, NM_001618, NM_173083, NM_003925, NM_014321, NM_024945, NM_014967, NM_139076, NM_000123, NM_005445, NM_001018115, NM_005441, NM_004628, NM_004629, NM_005591, NM_001006622, NM_079421, NM_001789, NM_145813, NM_001033, NM_032485, NM_005590, NM_175921, NM_022770, NM_030928, NM_001003686, NM_000234, NM_001953, NM_014502, NM_001790, NM_002553, NM_001379, NM_197962, NM_003503, NM_001375, NM_003504, NM_005395, NM_198971, NM_005132, NM_001893, NM_022809, NM_025193, NM_005431, NM_030755, NM_024068, NM_001034, NM_173627, NM_014481, NM_013975, NM_145043, NM_152463, NM_015920, NM_021218, NM_021830, NM_001641, NM_007370, NM_003362, NM_033222, NM_007027, NM_014071, NM_003621, NM_018248, NM_017858, NM_022092	2.05	1.01E-19
establishment of protein localization	5.38	NM_004162, NM_032520, NM_020673, NM_020150, NM_018844, NM_004355, NM_001013437, NM_006114, NM_001033002, NM_004910, NM_002093, NM_015231, NM_018087, NM_018314, NM_152841, NM_015517, NM_001042535, NM_020243, NM_000319, NM_007342, NM_012456, NM_005488, NM_015135, NM_004206, NM_003135, NM_014488, NM_016131, NM_018261, NM_152227, NM_003139, NM_170662, NM_005829, NM_006327, NM_001003786, NM_138361, NM_006325, NM_016343, NM_031482, NM_016338, NM_021168, NM_016553, NM_002868, NM_003128, NM_014933, NM_005667, NM_001025159, NM_152233, NM_001136232, NM_018571, NM_001001433, NM_017966, NM_024844, NM_144636, NM_015254, NM_006817, NM_000594, NM_001980, NM_032846, NM_001981, NM_020750, NM_002473, NM_002266, NM_001283, NM_002267, NM_194356, NM_002268, NM_012414, NM_014758, NM_018668, NM_145648, NM_001005753, NM_152244, NM_001660, NM_005796, NM_152240, NM_199344, NM_004343, NM_013374, NM_030805, NM_014044, NM_003666, NM_001970, NM_016106, NM_005370, NM_015049, NM_004069, NM_014669, NM_153485, NM_001008213, NM_024658, NM_018090, NM_212535, NM_003405, NM_032970, NM_014765, NM_018206, NM_004637, NM_012388, NM_006380, NM_022575, NM_018144, NM_033542, NM_018047, NM_020401, NM_003826, NM_006573, NM_014396, NM_005638, NM_032177, NM_001042581, NM_006827, NM_016226, NM_006826, NM_003292, NM_015869, NM_024057, NM_001031677, NM_012215, NM_001042588, NM_003299, NM_138712, NM_001813, NM_025132, NM_012486, NM_003917, NM_014700, NM_014453, NM_002455, NM_004935, NM_001037633, NM_178270, NM_015044, NM_198971, NM_004085, NM_015026, NM_033375, NM_003164, NM_025196, NM_015354, NM_013248, NM_031217, NM_001013839, NM_153719, NM_024923, NM_018230, NM_182916, NM_006178, NM_198883, NM_012479, NM_005698, NM_001098794, NM_005128, NM_005733, NM_001183, NM_031899, NM_024663, NM_006347, NM_004604, NM_014177, NM_013321, NM_004603, NM_016154, NM_006947, NM_018445, NM_001316, NM_004813, NM_004371, NM_014169, NM_016530, NM_138285, NM_005115, NM_003977	1.28	0.018074739
intracellular transport	4.85	NM_032520, NM_020150, NM_018844, NM_004355, NM_006114, NM_001033002, NM_002093, NM_015231, NM_152841, NM_001687, NM_001042535, NM_020243, NM_000319, NM_000271, NM_007342, NM_012456, NM_015327, NM_005488, NM_015135, NM_004206, NM_003135, NM_003139, NM_170662, NM_005829, NM_002911, NM_170665, NM_006327, NM_001003786, NM_006325, NM_000377, NM_017668, NM_005348, NM_016338, NM_017983, NM_003026, NM_198204, NM_003128, NM_014933, NM_014939, NM_001025159, NM_000046, NM_152233, NM_001136232, NM_018571, NM_138578, NM_001001433, NM_015254, NM_006817, NM_000594, NM_001980, NM_020750, NM_000430, NM_002473, NM_002266, NM_001283, NM_002267, NM_194356, NM_002268, NM_012414, NM_002470, NM_014232, NM_000633, NM_003011, NM_002475, NM_018384, NM_005796, NM_006597, NM_004343, NM_030808, NM_030805, NM_001970, NM_016106, NM_005370, NM_015049, NM_004069, NM_153485, NM_001008213, NM_024658, NM_033028, NM_003405, NM_032970, NM_014765, NM_018206, NM_012388, NM_080599, NM_005198, NM_006052, NM_006380, NM_000024, NM_022575, NM_018144, NM_153201, NM_032361, NM_018047, NM_020401, NM_003826, NM_014396, NM_005638, NM_012310, NM_032177, NM_001042581, NM_006827, NM_006826, NM_003292, NM_006432, NM_014251, NM_012215, NM_001042588, NM_005964, NM_025132, NM_003917, NM_002455, NM_015459, NM_004935, NM_001037633, NM_002355, NM_178270, NM_015044, NM_004085, NM_015026, NM_001177, NM_001081491, NM_003164, NM_025196, NM_007362, NM_020919, NM_030755, NM_013248, NM_015542, NM_018230, NM_182916, NM_001017963, NM_198883, NM_012479, NM_005698, NM_001098794, NM_005128, NM_015685, NM_006362, NM_001183, NM_012140, NM_005125, NM_005804, NM_024663, NM_006347, NM_004604, NM_004603, NM_005895, NM_006947, NM_001316, NM_018445, NM_004813, NM_004371, NM_130811, NM_001697, NM_003977	1.35	0.003432295
macromolecular complex assembly	5.41	NM_000165, NM_021059, NM_004781, NM_002293, NM_002014, NM_000801, NM_004355, NM_173515, NM_173039, NM_003534, NM_006109, NM_018310, NM_018087, NM_003531, NM_178014, NM_003537, NM_003535, NM_020243, NM_005483, NM_000319, NM_004865, NM_021062, NM_006009, NM_006938, NM_002495, NM_015135, NM_004774, NM_001079866, NM_001024465, NM_006473, NM_006026, NM_001070, NM_002105, NM_015721, NM_016447, NM_002106, NM_000377, NM_016343, NM_005348, NM_054014, NM_003543, NM_177925, NM_006925, NM_014166, NM_003542, NM_014165, NM_003545, NM_003544, NM_016338, NM_001042546, NM_015629, NM_005138, NM_003548, NM_001080975, NM_001902, NM_004719, NM_000249, NM_004140, NM_001025159, NM_005359, NM_032390, NM_016437, NM_001667, NM_003516, NM_003514, NM_032704, NM_003513, NM_000101, NM_003512, NM_003096, NM_003095, NM_018685, NM_002267, NM_003518, NM_015380, NM_003517, NM_182962, NM_001097577, NM_012177, NM_004793, NM_003011, NM_006000, NM_001013699, NM_001359, NM_080651, NM_000057, NM_001039619, NM_001034077, NM_005658, NM_002086, NM_004343, NM_002129, NM_001040874, NM_001144, NM_003524, NM_004029, NM_006087, NM_003521, NM_006086, NM_003522, NM_006088, NM_001293, NM_006082, NM_003529, NM_001042426, NM_012426, NM_014225, NM_181598, NM_004069, NM_006831, NM_005644, NM_024658, NM_003201, NM_002156, NM_014260, NM_018410, NM_014317, NM_001017980, NM_001752, NM_000024, NM_024102, NM_080593, NM_003826, NM_015465, NM_004726, NM_005441, NM_032177, NM_001042581, NM_024120, NM_001033, NM_001017990, NM_001042588, NM_001629, NM_001017991, NM_001813, NM_005969, NM_006232, NM_015459, NM_002154, NM_004935, NM_016101, NM_014708, NM_024831, NM_033091, NM_152854, NM_022745, NM_030752, NM_007362, NM_001025235, NM_004493, NM_004492, NM_199069, NM_181716, NM_138609, NM_001034, NM_001017963, NM_001005464, NM_005324, NM_002567, NM_001014840, NM_001123375, NM_000289, NM_004893, NM_007166, NM_032151, NM_004269, NM_021830, NM_003172, NM_003769, NM_001250, NM_006347, NM_032940, NM_004604, NM_005320, NM_005322, NM_014071, NM_001078166, NM_001316, NM_005318, NM_005319, NM_178191, NM_018154, NM_198576, NM_004169, NM_004552, NM_001014838, NM_005119, NM_004818	1.49	5.47E-06
macromolecular complex subunit organization	5.74	NM_021059, NM_002293, NM_004355, NM_173039, NM_003534, NM_003531, NM_003537, NM_003535, NM_021062, NM_005483, NM_002495, NM_001024465, NM_006473, NM_016447, NM_000377, NM_005348, NM_177925, NM_003543, NM_003542, NM_014166, NM_003545, NM_014165, NM_003544, NM_001042546, NM_003548, NM_001080975, NM_001902, NM_004719, NM_000249, NM_005359, NM_001025159, NM_016437, NM_003516, NM_003514, NM_003513, NM_032704, NM_003096, NM_003512, NM_000101, NM_003095, NM_022909, NM_018685, NM_002267, NM_003518, NM_003517, NM_015380, NM_182962, NM_012177, NM_080651, NM_001039619, NM_002086, NM_004343, NM_001040874, NM_003524, NM_003521, NM_006087, NM_006086, NM_006088, NM_003522, NM_006082, NM_001042426, NM_003529, NM_181598, NM_012426, NM_006831, NM_003201, NM_014317, NM_001752, NM_001017980, NM_000024, NM_024102, NM_004726, NM_001017990, NM_024120, NM_001629, NM_001017991, NM_006232, NM_014708, NM_024831, NM_033091, NM_152854, NM_001025235, NM_004493, NM_004492, NM_138609, NM_031217, NM_181716, NM_001017963, NM_001014840, NM_004893, NM_021830, NM_003769, NM_001250, NM_006845, NM_006347, NM_004604, NM_178191, NM_001014838, NM_000165, NM_004781, NM_002014, NM_000801, NM_173515, NM_001013437, NM_018310, NM_006109, NM_018087, NM_178014, NM_004865, NM_000319, NM_020243, NM_006009, NM_001545, NM_006938, NM_015135, NM_001079866, NM_004774, NM_006026, NM_001070, NM_015721, NM_002105, NM_002106, NM_016343, NM_054014, NM_006925, NM_016338, NM_005138, NM_015629, NM_004140, NM_032390, NM_001667, NM_203401, NM_001097577, NM_006000, NM_004793, NM_003011, NM_001013699, NM_001359, NM_000057, NM_005658, NM_019041, NM_001034077, NM_002129, NM_001144, NM_004029, NM_199177, NM_005563, NM_001293, NM_014225, NM_004069, NM_024658, NM_005644, NM_002156, NM_018410, NM_014260, NM_003826, NM_080593, NM_015465, NM_005441, NM_032177, NM_001042581, NM_001033, NM_001042588, NM_001813, NM_005969, NM_003594, NM_002154, NM_015459, NM_004935, NM_016101, NM_022745, NM_030752, NM_007362, NM_199069, NM_001034, NM_018230, NM_001005464, NM_002567, NM_005324, NM_000289, NM_001123375, NM_007166, NM_032151, NM_003172, NM_004269, NM_032940, NM_005320, NM_005322, NM_014071, NM_001078166, NM_001316, NM_005318, NM_005319, NM_018154, NM_198576, NM_004169, NM_004552, NM_005119, NM_004818	1.48	3.42E-06
mitotic cell cycle	5.18	NM_052988, NM_018454, NM_000601, NM_001255, NM_017518, NM_182620, NM_001013437, NM_018101, NM_021205, NM_002094, NM_078487, NM_001010932, NM_006341, NM_000075, NM_178014, NM_080668, NM_015517, NM_006530, NM_020242, NM_004159, NM_145060, NM_145061, NM_015329, NM_182513, NM_001269, NM_002497, NM_013299, NM_007044, NM_032637, NM_001168, NM_006325, NM_016343, NM_017785, NM_017668, NM_024094, NM_001007793, NM_006400, NM_078467, NM_004358, NM_018715, NM_004217, NM_004219, NM_001002800, NM_006503, NM_003707, NM_002795, NM_002794, NM_007317, NM_181042, NM_000389, NM_005983, NM_001012413, NM_030919, NM_000430, NM_002721, NM_014750, NM_002266, NM_018685, NM_138443, NM_003903, NM_024333, NM_002788, NM_002787, NM_203401, NM_012177, NM_000633, NM_001040694, NM_006396, NM_000057, NM_030900, NM_017760, NM_004701, NM_004336, NM_002691, NM_006600, NM_006086, NM_016195, NM_005789, NM_004856, NM_005563, NM_002791, NM_003318, NM_001042426, NM_006704, NM_199413, NM_003390, NM_004064, NM_016237, NM_012291, NM_003183, NM_015261, NM_198436, NM_015374, NM_198434, NM_018944, NM_018097, NM_002808, NM_001008938, NM_002809, NM_022346, NM_018492, NM_014814, NM_002804, NM_199246, NM_001005413, NM_002800, NM_014865, NM_002801, NM_005192, NM_005445, NM_003920, NM_004725, NM_002956, NM_079421, NM_001789, NM_024057, NM_001786, NM_139286, NM_013345, NM_001761, NM_014303, NM_004523, NM_002263, NM_003914, NM_002818, NM_001813, NM_001790, NM_018136, NM_001950, NM_018131, NM_001011699, NM_002816, NM_002810, NM_012112, NM_002812, NM_002358, NM_003503, NM_014708, NM_031299, NM_015169, NM_198971, NM_001042550, NM_007221, NM_022809, NM_145697, NM_001013836, NM_006461, NM_152132, NM_015282, NM_181716, NM_031217, NM_138484, NM_006263, NM_024808, NM_005030, NM_182687, NM_199122, NM_006559, NM_152562, NM_181803, NM_006845, NM_006101, NM_194260, NM_002648, NM_025180, NM_005805, NM_031423, NM_017858, NM_033417, NM_031966, NM_181800	2.56	1.61E-29
mRNA metabolic process	3.66	NM_173797, NM_006527, NM_005008, NM_005156, NM_138558, NM_001007230, NM_002094, NM_006109, NM_025265, NM_001033521, NM_003017, NM_006938, NM_015327, NM_003016, NM_001031732, NM_002911, NM_005826, NM_015721, NM_006328, NM_005346, NM_020528, NM_006925, NM_018077, NM_032102, NM_015629, NM_033109, NM_003142, NM_031203, NM_005839, NM_004719, NM_000249, NM_014740, NM_002138, NM_014884, NM_002896, NM_003096, NM_001127394, NM_003095, NM_003092, NM_001358, NM_001357, NM_001039619, NM_002687, NM_031263, NM_019108, NM_016424, NM_004501, NM_016199, NM_003089, NM_018109, NM_005850, NM_023010, NM_023011, NM_001293, NM_007279, NM_012426, NM_004596, NM_006831, NM_014871, NM_017612, NM_003407, NM_006445, NM_001112, NM_080599, NM_024102, NM_014110, NM_032864, NM_032361, NM_018047, NM_001077442, NM_015465, NM_080632, NM_032177, NM_001042581, NM_194247, NM_004247, NM_194431, NM_012199, NM_007006, NM_003587, NM_001042588, NM_014502, NM_006232, NM_005968, NM_003594, NM_001098477, NM_006372, NM_080687, NM_006627, NM_024831, NM_001031684, NM_006857, NM_015496, NM_012321, NM_007362, NM_152858, NM_052965, NM_014281, NM_001324, NM_004559, NM_001325, NM_015542, NM_003946, NM_001033505, NM_021177, NM_004697, NM_006559, NM_003769, NM_031369, NM_184234, NM_005804, NM_021190, NM_004960, NM_006347, NM_032940, NM_001078166, NM_024571, NM_001018069, NM_004814, NM_014829, NM_001017392, NM_032758, NM_004818	1.81	1.93E-08
mRNA processing	3.23	NM_173797, NM_006527, NM_005008, NM_138558, NM_005156, NM_001007230, NM_006109, NM_025265, NM_001033521, NM_003017, NM_006938, NM_003016, NM_001031732, NM_005826, NM_015721, NM_006328, NM_006925, NM_018077, NM_032102, NM_015629, NM_031203, NM_005839, NM_004719, NM_000249, NM_014740, NM_002138, NM_002896, NM_014884, NM_003096, NM_001127394, NM_003095, NM_003092, NM_001358, NM_001357, NM_002687, NM_001039619, NM_031263, NM_016424, NM_004501, NM_016199, NM_003089, NM_018109, NM_005850, NM_023010, NM_001293, NM_007279, NM_012426, NM_004596, NM_006831, NM_017612, NM_003407, NM_006445, NM_001112, NM_024102, NM_032864, NM_014110, NM_032361, NM_018047, NM_001077442, NM_015465, NM_080632, NM_032177, NM_001042581, NM_004247, NM_194247, NM_194431, NM_007006, NM_003587, NM_001042588, NM_014502, NM_006232, NM_005968, NM_003594, NM_001098477, NM_006372, NM_006627, NM_024831, NM_001031684, NM_006857, NM_015496, NM_012321, NM_152858, NM_007362, NM_052965, NM_014281, NM_001324, NM_004559, NM_001325, NM_003946, NM_001033505, NM_021177, NM_004697, NM_006559, NM_031369, NM_184234, NM_003769, NM_005804, NM_021190, NM_004960, NM_006347, NM_032940, NM_001078166, NM_024571, NM_004814, NM_014829, NM_001017392, NM_032758, NM_004818	1.84	8.43E-08
programmed cell death	4.32	NM_000165, NM_002305, NM_024900, NM_001066, NM_006281, NM_000600, NM_015331, NM_007051, NM_018844, NM_006875, NM_014959, NM_002094, NM_000417, NM_005605, NM_016561, NM_001024465, NM_024091, NM_145341, NM_198057, NM_144781, NM_003842, NM_001168, NM_021709, NM_014634, NM_021999, NM_031301, NM_001047160, NM_133328, NM_001013739, NM_016454, NM_002513, NM_005920, NM_024544, NM_015318, NM_003028, NM_016077, NM_001067, NM_015316, NM_001008540, NM_007315, NM_032992, NM_016640, NM_014341, NM_004330, NM_138578, NM_003467, NM_022157, NM_021127, NM_000595, NM_002727, NM_000051, NM_000594, NM_030782, NM_006698, NM_004708, NM_182962, NM_032983, NM_006290, NM_001092, NM_000633, NM_014430, NM_152240, NM_032989, NM_005658, NM_030900, NM_013374, NM_016424, NM_001033030, NM_005789, NM_016009, NM_003311, NM_006705, NM_024755, NM_018098, NM_001037333, NM_004064, NM_201559, NM_002157, NM_012291, NM_002156, NM_014762, NM_003879, NM_021960, NM_033027, NM_145018, NM_033339, NM_015367, NM_024051, NM_003820, NM_024948, NM_004740, NM_003680, NM_006570, NM_003789, NM_001621, NM_133171, NM_079421, NM_145813, NM_201430, NM_013258, NM_133369, NM_003810, NM_005041, NM_012486, NM_006427, NM_024065, NM_001226, NM_197962, NM_004935, NM_001375, NM_019887, NM_002654, NM_001031685, NM_198120, NM_001013843, NM_001040668, NM_182471, NM_004089, NM_032477, NM_003897, NM_003374, NM_001098525, NM_033294, NM_014281, NM_201397, NM_003946, NM_003805, NM_003804, NM_199122, NM_005428, NM_003768, NM_003217, NM_198282, NM_139266, NM_031365, NM_001316, NM_002583, NM_005118, NM_012138	1.29	0.03875463
protein localization	6.23	NM_012461, NM_032520, NM_031885, NM_007051, NM_004355, NM_004910, NM_001033002, NM_002093, NM_006342, NM_001042535, NM_007342, NM_012456, NM_005488, NM_004206, NM_003135, NM_018261, NM_016131, NM_003139, NM_007044, NM_006327, NM_001003786, NM_006325, NM_138361, NM_031482, NM_001040439, NM_004356, NM_021168, NM_016553, NM_002868, NM_004217, NM_014933, NM_003128, NM_001025159, NM_001001433, NM_024844, NM_032846, NM_002473, NM_002266, NM_002267, NM_194356, NM_002268, NM_012414, NM_018668, NM_005796, NM_199344, NM_004343, NM_022440, NM_016009, NM_003666, NM_014044, NM_016106, NM_005370, NM_153485, NM_001008213, NM_018090, NM_004637, NM_012388, NM_006380, NM_022575, NM_020401, NM_006573, NM_006827, NM_006826, NM_016226, NM_138712, NM_012486, NM_014700, NM_001001716, NM_002371, NM_033375, NM_025196, NM_031217, NM_013248, NM_153719, NM_024923, NM_198883, NM_006178, NM_001014840, NM_005698, NM_012479, NM_001098794, NM_005733, NM_181353, NM_031899, NM_006347, NM_004604, NM_013321, NM_004603, NM_016154, NM_006947, NM_016530, NM_001014838, NM_004162, NM_020150, NM_020673, NM_018844, NM_001013437, NM_006114, NM_018087, NM_015231, NM_152841, NM_018314, NM_015517, NM_018080, NM_000319, NM_020243, NM_002503, NM_015135, NM_014488, NM_152227, NM_170662, NM_005829, NM_016343, NM_017785, NM_016338, NM_005667, NM_152233, NM_001136232, NM_018571, NM_017966, NM_144636, NM_006817, NM_015254, NM_000594, NM_002727, NM_001980, NM_020750, NM_001981, NM_001283, NM_152524, NM_014758, NM_018975, NM_000835, NM_001005753, NM_145648, NM_001457, NM_152244, NM_001660, NM_152240, NM_013374, NM_030805, NM_001970, NM_004069, NM_015049, NM_014669, NM_024658, NM_014762, NM_212535, NM_033028, NM_003405, NM_015373, NM_014765, NM_032970, NM_018206, NM_018144, NM_033542, NM_018047, NM_003826, NM_014396, NM_005638, NM_032177, NM_001042581, NM_015869, NM_003292, NM_012215, NM_001031677, NM_024057, NM_001042588, NM_003299, NM_025132, NM_001813, NM_003917, NM_014453, NM_002455, NM_004935, NM_001037633, NM_015044, NM_178270, NM_198971, NM_004085, NM_015026, NM_003164, NM_020919, NM_015354, NM_001013839, NM_182916, NM_018230, NM_005128, NM_001183, NM_024663, NM_006101, NM_014177, NM_003565, NM_001316, NM_018445, NM_004813, NM_004371, NM_014169, NM_138285, NM_005115, NM_003977	1.29	0.00398332

**protein transport**	5.31	NM_004162, NM_032520, NM_020673, NM_020150, NM_018844, NM_004355, NM_001013437, NM_006114, NM_001033002, NM_004910, NM_002093, NM_015231, NM_018087, NM_018314, NM_152841, NM_001042535, NM_020243, NM_000319, NM_007342, NM_012456, NM_005488, NM_015135, NM_004206, NM_003135, NM_014488, NM_016131, NM_018261, NM_152227, NM_003139, NM_170662, NM_005829, NM_006327, NM_001003786, NM_138361, NM_006325, NM_016343, NM_031482, NM_016338, NM_021168, NM_016553, NM_002868, NM_003128, NM_014933, NM_005667, NM_001025159, NM_152233, NM_001136232, NM_018571, NM_001001433, NM_017966, NM_024844, NM_144636, NM_015254, NM_006817, NM_000594, NM_001980, NM_032846, NM_001981, NM_020750, NM_002473, NM_002266, NM_001283, NM_002267, NM_194356, NM_002268, NM_012414, NM_014758, NM_018668, NM_145648, NM_001005753, NM_152244, NM_001660, NM_005796, NM_152240, NM_199344, NM_004343, NM_013374, NM_030805, NM_014044, NM_003666, NM_001970, NM_016106, NM_005370, NM_015049, NM_004069, NM_014669, NM_153485, NM_001008213, NM_024658, NM_018090, NM_212535, NM_003405, NM_032970, NM_014765, NM_018206, NM_004637, NM_012388, NM_006380, NM_022575, NM_018144, NM_033542, NM_018047, NM_020401, NM_003826, NM_006573, NM_014396, NM_005638, NM_032177, NM_001042581, NM_006827, NM_016226, NM_006826, NM_003292, NM_015869, NM_024057, NM_001031677, NM_012215, NM_001042588, NM_003299, NM_138712, NM_025132, NM_012486, NM_003917, NM_014700, NM_014453, NM_002455, NM_004935, NM_001037633, NM_178270, NM_015044, NM_004085, NM_015026, NM_033375, NM_003164, NM_025196, NM_015354, NM_013248, NM_031217, NM_001013839, NM_153719, NM_024923, NM_018230, NM_182916, NM_006178, NM_198883, NM_012479, NM_005698, NM_001098794, NM_005128, NM_005733, NM_001183, NM_031899, NM_024663, NM_006347, NM_004604, NM_014177, NM_013321, NM_004603, NM_016154, NM_006947, NM_018445, NM_001316, NM_004813, NM_004371, NM_014169, NM_016530, NM_138285, NM_005115, NM_003977	1.28	0.022014752
**regulation of apoptosis**	5.87	NM_002305, NM_000601, NM_006281, NM_000600, NM_015331, NM_007051, NM_004355, NM_006875, NM_014959, NM_002094, NM_002093, NM_198175, NM_001010932, NM_178014, NM_004964, NM_000417, NM_000477, NM_005605, NM_001540, NM_000882, NM_016561, NM_001024465, NM_000179, NM_004155, NM_032637, NM_006472, NM_003842, NM_014739, NM_001168, NM_021709, NM_021999, NM_005346, NM_002715, NM_031301, NM_001047160, NM_133328, NM_022168, NM_078467, NM_002513, NM_016553, NM_001007593, NM_020920, NM_024544, NM_015318, NM_181696, NM_001067, NM_004879, NM_015316, NM_000249, NM_007315, NM_032992, NM_032431, NM_001025159, NM_000389, NM_019071, NM_021141, NM_001018677, NM_014341, NM_004330, NM_018571, NM_138578, NM_005983, NM_147173, NM_000598, NM_021127, NM_006079, NM_000595, NM_000594, NM_000051, NM_006793, NM_000961, NM_001908, NM_006811, NM_182962, NM_004708, NM_032983, NM_006290, NM_001092, NM_000633, NM_001605, NM_018384, NM_021972, NM_014430, NM_004448, NM_002890, NM_152240, NM_032989, NM_005658, NM_004343, NM_022440, NM_006708, NM_001033030, NM_147184, NM_016009, NM_005789, NM_002133, NM_006088, NM_001970, NM_181738, NM_002982, NM_014225, NM_016291, NM_018098, NM_001007277, NM_002634, NM_004064, NM_201559, NM_152405, NM_002157, NM_002156, NM_003183, NM_014762, NM_003879, NM_032494, NM_021960, NM_003877, NM_001752, NM_172014, NM_058246, NM_015367, NM_003925, NM_001018077, NM_000024, NM_001083617, NM_004309, NM_000022, NM_080923, NM_000123, NM_004740, NM_002448, NM_012094, NM_006573, NM_016037, NM_003789, NM_181652, NM_079421, NM_145813, NM_003295, NM_001786, NM_003883, NM_003299, NM_013258, NM_003885, NM_005238, NM_000758, NM_003810, NM_000852, NM_020310, NM_012486, NM_006427, NM_001226, NM_003955, NM_004935, NM_006378, NM_019887, NM_032374, NM_004417, NM_001031685, NM_002371, NM_005431, NM_003897, NM_030755, NM_033294, NM_004379, NM_001731, NM_005531, NM_153719, NM_201397, NM_004280, NM_003946, NM_003805, NM_003804, NM_015920, NM_003807, NM_005428, NM_003768, NM_003217, NM_021935, NM_001189, NM_002648, NM_139266, NM_018441, NM_031365, NM_018445, NM_002583, NM_001104, NM_018941, NM_004551, NM_005118, NM_012138	1.34	0.001259982
**regulation of cell cycle**	3.43	NM_052988, NM_018454, NM_001923, NM_023015, NM_078487, NM_006109, NM_006342, NM_000075, NM_006341, NM_004964, NM_015895, NM_015517, NM_013376, NM_145341, NM_002497, NM_001269, NM_032637, NM_002105, NM_001168, NM_016343, NM_016446, NM_006925, NM_001007793, NM_078468, NM_001527, NM_078467, NM_015316, NM_000389, NM_001018677, NM_005983, NM_006079, NM_000595, NM_000051, NM_000594, NM_015113, NM_014750, NM_018685, NM_003903, NM_016567, NM_012177, NM_000633, NM_001031716, NM_021972, NM_000057, NM_001039619, NM_006597, NM_004343, NM_012406, NM_004336, NM_016195, NM_006806, NM_003318, NM_016290, NM_006705, NM_181839, NM_004064, NM_012291, NM_003183, NM_014264, NM_001826, NM_001827, NM_139076, NM_199246, NM_001005413, NM_080923, NM_005192, NM_153201, NM_016038, NM_015057, NM_003920, NM_004725, NM_004628, NM_006823, NM_004629, NM_079421, NM_001789, NM_003292, NM_001786, NM_014303, NM_030928, NM_003883, NM_001760, NM_003885, NM_015276, NM_005238, NM_001813, NM_001790, NM_020310, NM_002358, NM_004935, NM_003503, NM_003504, NM_014708, NM_198971, NM_001896, NM_024602, NM_001031685, NM_022809, NM_001013836, NM_024068, NM_018164, NM_182687, NM_002567, NM_024800, NM_015920, NM_181803, NM_002648, NM_025180, NM_017858, NM_012341, NM_012138, NM_181800, NM_031966, NM_015201	1.90	4.47E-09
**regulation of cell death**	5.94	NM_002305, NM_000601, NM_006281, NM_000600, NM_015331, NM_007051, NM_004355, NM_006875, NM_014959, NM_002094, NM_002093, NM_198175, NM_001010932, NM_178014, NM_004964, NM_000417, NM_000477, NM_005605, NM_001540, NM_000882, NM_016561, NM_001024465, NM_000179, NM_004155, NM_032637, NM_006472, NM_003842, NM_014739, NM_001168, NM_021709, NM_021999, NM_005346, NM_002715, NM_031301, NM_001047160, NM_133328, NM_022168, NM_078467, NM_002513, NM_016553, NM_001007593, NM_020920, NM_024544, NM_015318, NM_181696, NM_001067, NM_004879, NM_015316, NM_000249, NM_007315, NM_032992, NM_032431, NM_001025159, NM_000389, NM_019071, NM_021141, NM_001018677, NM_014341, NM_004330, NM_018571, NM_138578, NM_005983, NM_147173, NM_000598, NM_021127, NM_006079, NM_000595, NM_000594, NM_000051, NM_006793, NM_000961, NM_001908, NM_006811, NM_182962, NM_004708, NM_032983, NM_006290, NM_001092, NM_000633, NM_001605, NM_018384, NM_021972, NM_014430, NM_004448, NM_002890, NM_152240, NM_032989, NM_005658, NM_004343, NM_022440, NM_006708, NM_001033030, NM_001151, NM_147184, NM_016009, NM_005789, NM_002133, NM_006088, NM_001970, NM_181738, NM_002982, NM_014225, NM_016291, NM_018098, NM_001007277, NM_002634, NM_004064, NM_201559, NM_152405, NM_002157, NM_002156, NM_003183, NM_014762, NM_003879, NM_032494, NM_021960, NM_003877, NM_001752, NM_172014, NM_058246, NM_015367, NM_003925, NM_001018077, NM_000024, NM_001083617, NM_004309, NM_000022, NM_080923, NM_000123, NM_004740, NM_002448, NM_012094, NM_006573, NM_016037, NM_006570, NM_003789, NM_181652, NM_079421, NM_145813, NM_003295, NM_001786, NM_003883, NM_003299, NM_013258, NM_003885, NM_005238, NM_000758, NM_003810, NM_000852, NM_020310, NM_012486, NM_006427, NM_001226, NM_003955, NM_004935, NM_006378, NM_019887, NM_032374, NM_004417, NM_001031685, NM_002371, NM_005431, NM_003897, NM_030755, NM_033294, NM_004379, NM_001731, NM_005531, NM_153719, NM_201397, NM_004280, NM_003946, NM_003805, NM_003804, NM_015920, NM_003807, NM_005428, NM_003768, NM_003217, NM_021935, NM_001189, NM_002648, NM_139266, NM_018441, NM_031365, NM_018445, NM_002583, NM_001104, NM_018941, NM_004551, NM_005118, NM_012138	1.33	0.001243153
**regulation of programmed cell death**	5.87	NM_002305, NM_000601, NM_006281, NM_000600, NM_015331, NM_007051, NM_004355, NM_006875, NM_014959, NM_002094, NM_002093, NM_198175, NM_001010932, NM_178014, NM_004964, NM_000417, NM_000477, NM_005605, NM_001540, NM_000882, NM_016561, NM_001024465, NM_000179, NM_004155, NM_032637, NM_006472, NM_003842, NM_014739, NM_001168, NM_021709, NM_021999, NM_005346, NM_002715, NM_031301, NM_001047160, NM_133328, NM_022168, NM_078467, NM_002513, NM_016553, NM_001007593, NM_020920, NM_024544, NM_015318, NM_181696, NM_001067, NM_004879, NM_015316, NM_000249, NM_007315, NM_032992, NM_032431, NM_001025159, NM_000389, NM_019071, NM_021141, NM_001018677, NM_014341, NM_004330, NM_018571, NM_138578, NM_005983, NM_147173, NM_000598, NM_021127, NM_006079, NM_000595, NM_000594, NM_000051, NM_006793, NM_000961, NM_001908, NM_006811, NM_182962, NM_004708, NM_032983, NM_006290, NM_001092, NM_000633, NM_001605, NM_018384, NM_021972, NM_014430, NM_004448, NM_002890, NM_152240, NM_032989, NM_005658, NM_004343, NM_022440, NM_006708, NM_001033030, NM_147184, NM_016009, NM_005789, NM_002133, NM_006088, NM_001970, NM_181738, NM_002982, NM_014225, NM_016291, NM_018098, NM_001007277, NM_002634, NM_004064, NM_201559, NM_152405, NM_002157, NM_002156, NM_003183, NM_014762, NM_003879, NM_032494, NM_021960, NM_003877, NM_001752, NM_172014, NM_058246, NM_015367, NM_003925, NM_001018077, NM_000024, NM_001083617, NM_004309, NM_000022, NM_080923, NM_000123, NM_004740, NM_002448, NM_012094, NM_006573, NM_016037, NM_003789, NM_181652, NM_079421, NM_145813, NM_003295, NM_001786, NM_003883, NM_003299, NM_013258, NM_003885, NM_005238, NM_000758, NM_003810, NM_000852, NM_020310, NM_012486, NM_006427, NM_001226, NM_003955, NM_004935, NM_006378, NM_019887, NM_032374, NM_004417, NM_001031685, NM_002371, NM_005431, NM_003897, NM_030755, NM_033294, NM_004379, NM_001731, NM_005531, NM_153719, NM_201397, NM_004280, NM_003946, NM_003805, NM_003804, NM_015920, NM_003807, NM_005428, NM_003768, NM_003217, NM_021935, NM_001189, NM_002648, NM_139266, NM_018441, NM_031365, NM_018445, NM_002583, NM_001104, NM_018941, NM_004551, NM_005118, NM_012138	1.32	0.00210217
**response to DNA damage stimulus**	3.86	NM_003350, NM_003146, NM_017518, NM_006666, NM_001923, NM_001033002, NM_002907, NM_023015, NM_002876, NM_015517, NM_005483, NM_005484, NM_001042618, NM_006479, NM_199420, NM_005485, NM_001024465, NM_000179, NM_006020, NM_006027, NM_001071, NM_002911, NM_032444, NM_002105, NM_016448, NM_006328, NM_002916, NM_018319, NM_001003931, NM_001001655, NM_058216, NM_078468, NM_078467, NM_181573, NM_152633, NM_181471, NM_001042697, NM_001067, NM_004219, NM_182640, NM_000249, NM_000247, NM_001990, NM_007317, NM_000389, NM_021141, NM_001080449, NM_030811, NM_002896, NM_002528, NM_080649, NM_198948, NM_001127218, NM_000051, NM_006515, NM_030660, NM_018975, NM_016567, NM_178150, NM_001031716, NM_014430, NM_000057, NM_182776, NM_006297, NM_152240, NM_002129, NM_006304, NM_004029, NM_002691, NM_080911, NM_002692, NM_002693, NM_002133, NM_018193, NM_013282, NM_016290, NM_004237, NM_006705, NM_002947, NM_002946, NM_002945, NM_201559, NM_152405, NM_021821, NM_003579, NM_014311, NM_001618, NM_198954, NM_003925, NM_139076, NM_014967, NM_000123, NM_005445, NM_003920, NM_001018115, NM_005441, NM_004628, NM_004629, NM_005591, NM_001006622, NM_079421, NM_145813, NM_001786, NM_005590, NM_001003686, NM_000234, NM_014502, NM_005395, NM_198971, NM_001893, NM_005431, NM_024068, NM_005531, NM_173627, NM_014481, NM_013975, NM_145043, NM_152463, NM_024800, NM_015920, NM_021218, NM_001641, NM_007370, NM_003362, NM_025180, NM_007027, NM_014071, NM_018248, NM_017858, NM_012138	1.89	3.19E-10
**RNA processing**	5.87	NM_173797, NM_144982, NM_006527, NM_019037, NM_005008, NM_017706, NM_005156, NM_018256, NM_138558, NM_016001, NM_017793, NM_001007230, NM_023015, NM_001033714, NM_006109, NM_031472, NM_025265, NM_001033521, NM_006938, NM_003017, NM_003016, NM_001031732, NM_005826, NM_015721, NM_006328, NM_019083, NM_019082, NM_013393, NM_006925, NM_002715, NM_058219, NM_018077, NM_032102, NM_020158, NM_133328, NM_015629, NM_133644, NM_033109, NM_025065, NM_003142, NM_198202, NM_031203, NM_015004, NM_018072, NM_005839, NM_032993, NM_033661, NM_004719, NM_000249, NM_014740, NM_003703, NM_018127, NM_002138, NM_005837, NM_014884, NM_002896, NM_181581, NM_022157, NM_014886, NM_003096, NM_005777, NM_002897, NM_001127394, NM_003095, NM_003092, NM_001031712, NM_001358, NM_001357, NM_001605, NM_022915, NM_003729, NM_017646, NM_017647, NM_001039619, NM_002687, NM_018285, NM_006392, NM_004704, NM_005791, NM_031263, NM_016424, NM_019042, NM_005371, NM_004501, NM_016199, NM_003089, NM_018109, NM_005850, NM_023010, NM_001293, NM_007279, NM_012426, NM_018983, NM_014225, NM_001363, NM_004596, NM_015659, NM_006831, NM_003407, NM_017612, NM_020175, NM_006445, NM_001002019, NM_001112, NM_015908, NM_024102, NM_014110, NM_032864, NM_003683, NM_032361, NM_018047, NM_015056, NM_016038, NM_001077442, NM_015465, NM_016037, NM_080632, NM_032177, NM_001042581, NM_194247, NM_004247, NM_001436, NM_194431, NM_007006, NM_003587, NM_001042588, NM_014303, NM_017838, NM_014502, NM_005968, NM_006232, NM_003594, NM_003819, NM_001098477, NM_006567, NM_006372, NM_175066, NM_006627, NM_024831, NM_001031684, NM_006857, NM_015496, NM_012321, NM_025193, NM_007362, NM_152858, NM_015029, NM_004493, NM_014285, NM_001034194, NM_052965, NM_004559, NM_001324, NM_014281, NM_001325, NM_001039937, NM_182916, NM_014793, NM_022366, NM_003946, NM_022805, NM_001033505, NM_012123, NM_017722, NM_004697, NM_021177, NM_006170, NM_006559, NM_003769, NM_184234, NM_031369, NM_005804, NM_004960, NM_021190, NM_006347, NM_032940, NM_001078166, NM_024571, NM_006453, NM_014473, NM_004814, NM_058163, NM_020230, NM_014829, NM_006649, NM_033416, NM_001017392, NM_015201, NM_032758, NM_004818	1.97	2.93E-18
**cell division**	3.83	NM_052988, NM_018454, NM_001255, NM_017518, NM_182620, NM_001013437, NM_018101, NM_000075, NM_006341, NM_080668, NM_006739, NM_016948, NM_145060, NM_015329, NM_145061, NM_182513, NM_002497, NM_001269, NM_013299, NM_007044, NM_001168, NM_006325, NM_016343, NM_017668, NM_006729, NM_018719, NM_004358, NM_018715, NM_004217, NM_002710, NM_004219, NM_001002800, NM_024322, NM_003707, NM_005667, NM_001012413, NM_030919, NM_001980, NM_022909, NM_000430, NM_002473, NM_018685, NM_152524, NM_194356, NM_003903, NM_138443, NM_024333, NM_013277, NM_012177, NM_001005753, NM_001040694, NM_006396, NM_002890, NM_017760, NM_013374, NM_004701, NM_004336, NM_016195, NM_006600, NM_004856, NM_199413, NM_033331, NM_003390, NM_145800, NM_016237, NM_012291, NM_015261, NM_033028, NM_018944, NM_018097, NM_022346, NM_001008938, NM_001826, NM_001827, NM_199246, NM_001005413, NM_014865, NM_005445, NM_003920, NM_001789, NM_024057, NM_001786, NM_001761, NM_139286, NM_001760, NM_000234, NM_004523, NM_002263, NM_003914, NM_005964, NM_001813, NM_001790, NM_018136, NM_001011699, NM_018131, NM_002358, NM_004935, NM_003503, NM_031299, NM_014708, NM_001042550, NM_007221, NM_022809, NM_001037281, NM_145697, NM_001013836, NM_006461, NM_015282, NM_181716, NM_138484, NM_024808, NM_005030, NM_013975, NM_152562, NM_181803, NM_006101, NM_194260, NM_025180, NM_031423, NM_017858, NM_033417, NM_012138, NM_181800, NM_031966	2.37	3.07E-18
**cell proliferation**	3.46	NM_003350, NM_000601, NM_000801, NM_004577, NM_001040138, NM_004355, NM_138558, NM_018256, NM_006875, NM_007053, NM_002201, NM_006109, NM_001010932, NM_006342, NM_000417, NM_000884, NM_020242, NM_000883, NM_015013, NM_003749, NM_003035, NM_032637, NM_016343, NM_017668, NM_003744, NM_054014, NM_001007793, NM_005211, NM_018171, NM_006400, NM_004356, NM_002417, NM_181696, NM_003028, NM_004598, NM_003851, NM_001008540, NM_001025159, NM_021141, NM_005983, NM_003467, NM_002893, NM_001981, NM_000430, NM_014750, NM_003903, NM_013277, NM_006494, NM_000633, NM_004448, NM_199184, NM_182776, NM_001039619, NM_021976, NM_032989, NM_006889, NM_004335, NM_003088, NM_012406, NM_004336, NM_030808, NM_000585, NM_006600, NM_002133, NM_001552, NM_003666, NM_013282, NM_181738, NM_001363, NM_198336, NM_002156, NM_172014, NM_033023, NM_001826, NM_015908, NM_001827, NM_080923, NM_014110, NM_016038, NM_004725, NM_006573, NM_007111, NM_000235, NM_001789, NM_001030, NM_014303, NM_001760, NM_001127709, NM_003885, NM_003377, NM_007109, NM_005964, NM_014502, NM_001790, NM_001033056, NM_018136, NM_006624, NM_012112, NM_004935, NM_152854, NM_022809, NM_030755, NM_006764, NM_005531, NM_201397, NM_005030, NM_003807, NM_006559, NM_001250, NM_006845, NM_021935, NM_005807, NM_002648, NM_001311, NM_031365, NM_001316, NM_015201	1.45	0.002397965
**cellular macromolecule catabolic process**	5.18	NM_003350, NM_001255, NM_015331, NM_001923, NM_017703, NM_007051, NM_138558, NM_002201, NM_003730, NM_001007230, NM_002094, NM_203467, NM_006936, NM_018314, NM_015984, NM_153341, NM_004159, NM_148177, NM_015327, NM_022039, NM_006537, NM_170662, NM_003342, NM_002911, NM_018998, NM_032637, NM_032444, NM_080678, NM_016448, NM_138361, NM_005346, NM_003745, NM_198157, NM_172070, NM_080875, NM_031482, NM_001007793, NM_031301, NM_133328, NM_021168, NM_033109, NM_024544, NM_015004, NM_012164, NM_152329, NM_006503, NM_000249, NM_014740, NM_002795, NM_002794, NM_032431, NM_022457, NM_001080449, NM_002138, NM_001033024, NM_005983, NM_006321, NM_002528, NM_000051, NM_015255, NM_012174, NM_002473, NM_134265, NM_001005849, NM_001009934, NM_003903, NM_002788, NM_002931, NM_002787, NM_006290, NM_178150, NM_012177, NM_004793, NM_006397, NM_001040876, NM_019108, NM_001144, NM_015435, NM_016297, NM_023010, NM_005789, NM_023011, NM_002791, NM_013282, NM_006704, NM_022079, NM_002947, NM_002946, NM_002945, NM_014871, NM_005647, NM_003407, NM_016237, NM_003183, NM_012308, NM_003877, NM_016617, NM_014311, NM_002808, NM_002809, NM_014819, NM_003925, NM_080599, NM_014814, NM_002804, NM_002800, NM_014110, NM_000123, NM_002801, NM_021633, NM_015057, NM_018561, NM_004725, NM_004628, NM_015176, NM_080632, NM_005101, NM_024646, NM_016172, NM_014674, NM_145813, NM_012199, NM_001786, NM_139286, NM_183419, NM_015276, NM_003299, NM_002818, NM_025133, NM_012486, NM_002816, NM_003819, NM_024955, NM_002810, NM_020765, NM_080687, NM_003955, NM_002812, NM_002358, NM_001375, NM_031299, NM_178270, NM_024602, NM_007362, NM_015029, NM_152132, NM_001109662, NM_006462, NM_013316, NM_000527, NM_015542, NM_006263, NM_178588, NM_025241, NM_199129, NM_020799, NM_031369, NM_181803, NM_194260, NM_005805, NM_014176, NM_031229, NM_018445, NM_024087, NM_145032, NM_018941, NM_022818, NM_152550, NM_031966, NM_181800	1.31	0.009334563
**DNA repair**	3.17	NM_003350, NM_003146, NM_017518, NM_001923, NM_006666, NM_001033002, NM_002907, NM_023015, NM_002876, NM_015517, NM_005483, NM_005484, NM_001042618, NM_006479, NM_005485, NM_199420, NM_001024465, NM_000179, NM_006020, NM_006027, NM_001071, NM_032444, NM_002911, NM_002105, NM_006328, NM_018319, NM_001003931, NM_002916, NM_058216, NM_001001655, NM_078468, NM_152633, NM_181573, NM_181471, NM_001042697, NM_001067, NM_004219, NM_000249, NM_001990, NM_007317, NM_021141, NM_001080449, NM_002896, NM_080649, NM_002528, NM_198948, NM_001127218, NM_000051, NM_030660, NM_006515, NM_016567, NM_178150, NM_001031716, NM_000057, NM_006297, NM_002129, NM_006304, NM_002691, NM_002692, NM_080911, NM_002693, NM_018193, NM_013282, NM_016290, NM_006705, NM_004237, NM_002947, NM_002946, NM_002945, NM_152405, NM_003579, NM_014311, NM_198954, NM_001618, NM_003925, NM_014967, NM_139076, NM_000123, NM_005445, NM_005441, NM_001018115, NM_004628, NM_004629, NM_005591, NM_001006622, NM_079421, NM_005590, NM_001003686, NM_000234, NM_014502, NM_005395, NM_198971, NM_001893, NM_005431, NM_024068, NM_173627, NM_014481, NM_013975, NM_145043, NM_152463, NM_015920, NM_021218, NM_001641, NM_007370, NM_003362, NM_007027, NM_014071, NM_018248	2.04	3.29E-10

**M phase**	4.85	NM_018454, NM_000601, NM_001255, NM_017518, NM_182620, NM_001013437, NM_006875, NM_018101, NM_001010932, NM_006342, NM_002876, NM_006341, NM_178014, NM_080668, NM_006530, NM_020242, NM_032117, NM_145060, NM_145061, NM_015329, NM_000179, NM_182513, NM_152431, NM_001269, NM_002497, NM_006027, NM_013299, NM_007044, NM_001070, NM_198795, NM_002105, NM_001168, NM_006325, NM_016343, NM_017785, NM_017668, NM_024094, NM_058216, NM_001007793, NM_006400, NM_004358, NM_002417, NM_018715, NM_004217, NM_004219, NM_001002800, NM_000249, NM_003707, NM_007317, NM_181042, NM_001012413, NM_000051, NM_030919, NM_004516, NM_015255, NM_000430, NM_002473, NM_014750, NM_002266, NM_152524, NM_018685, NM_138443, NM_003903, NM_007068, NM_024333, NM_203401, NM_012177, NM_001040694, NM_006396, NM_017760, NM_004036, NM_004701, NM_004336, NM_006600, NM_006086, NM_016195, NM_004856, NM_005563, NM_003318, NM_006704, NM_199413, NM_152513, NM_004237, NM_002945, NM_003390, NM_016237, NM_012291, NM_015261, NM_003579, NM_198436, NM_015374, NM_198434, NM_018944, NM_018097, NM_001008938, NM_022346, NM_018492, NM_001827, NM_199246, NM_001005413, NM_014865, NM_005445, NM_003920, NM_004725, NM_001018115, NM_002956, NM_005591, NM_012218, NM_001789, NM_024057, NM_001786, NM_005590, NM_139286, NM_001761, NM_014303, NM_003883, NM_004523, NM_002263, NM_003914, NM_001813, NM_001790, NM_018136, NM_018131, NM_001011699, NM_012112, NM_002358, NM_014708, NM_031299, NM_015169, NM_001042550, NM_005132, NM_007221, NM_022809, NM_025193, NM_145697, NM_005431, NM_001013836, NM_006461, NM_015282, NM_181716, NM_031217, NM_138484, NM_024808, NM_005030, NM_182687, NM_013975, NM_004943, NM_006031, NM_152562, NM_181803, NM_006845, NM_006101, NM_194260, NM_025180, NM_031423, NM_017858, NM_033417, NM_031966, NM_181800	2.70	3.22E-30
**M phase of mitotic cell cycle**	3.59	NM_018454, NM_000601, NM_001255, NM_017518, NM_182620, NM_001013437, NM_018101, NM_001010932, NM_006341, NM_178014, NM_080668, NM_006530, NM_020242, NM_145060, NM_015329, NM_145061, NM_182513, NM_002497, NM_001269, NM_013299, NM_007044, NM_001168, NM_006325, NM_016343, NM_017785, NM_017668, NM_024094, NM_001007793, NM_006400, NM_004358, NM_018715, NM_004217, NM_004219, NM_001002800, NM_003707, NM_007317, NM_181042, NM_001012413, NM_030919, NM_000430, NM_014750, NM_018685, NM_003903, NM_138443, NM_024333, NM_012177, NM_001040694, NM_006396, NM_017760, NM_004701, NM_004336, NM_006086, NM_016195, NM_006600, NM_004856, NM_006704, NM_003390, NM_016237, NM_012291, NM_015261, NM_198436, NM_198434, NM_018944, NM_018097, NM_022346, NM_001008938, NM_018492, NM_199246, NM_001005413, NM_014865, NM_005445, NM_003920, NM_004725, NM_002956, NM_001789, NM_024057, NM_001786, NM_001761, NM_014303, NM_139286, NM_004523, NM_002263, NM_003914, NM_001813, NM_001790, NM_018136, NM_001011699, NM_018131, NM_012112, NM_002358, NM_031299, NM_014708, NM_015169, NM_001042550, NM_007221, NM_022809, NM_145697, NM_001013836, NM_006461, NM_015282, NM_031217, NM_181716, NM_138484, NM_024808, NM_005030, NM_182687, NM_152562, NM_181803, NM_006101, NM_006845, NM_194260, NM_025180, NM_031423, NM_017858, NM_033417, NM_181800, NM_031966	2.94	6.49E-26
**macromolecule catabolic process**	5.54	NM_003350, NM_001255, NM_015331, NM_000801, NM_001923, NM_017703, NM_007051, NM_138558, NM_002201, NM_003730, NM_001007230, NM_002094, NM_203467, NM_006936, NM_018314, NM_015984, NM_153341, NM_004159, NM_148177, NM_015327, NM_022039, NM_006537, NM_170662, NM_003342, NM_018998, NM_032637, NM_032444, NM_002911, NM_080678, NM_016448, NM_138361, NM_005346, NM_003745, NM_198157, NM_054014, NM_172070, NM_080875, NM_031482, NM_001007793, NM_031301, NM_133328, NM_002862, NM_021168, NM_033109, NM_024544, NM_015004, NM_012164, NM_152329, NM_006503, NM_000249, NM_014740, NM_002795, NM_002794, NM_032431, NM_022457, NM_001080449, NM_002138, NM_002076, NM_001033024, NM_005983, NM_006321, NM_002528, NM_000051, NM_000391, NM_015255, NM_012174, NM_002473, NM_134265, NM_001005849, NM_001009934, NM_003903, NM_002788, NM_002787, NM_002931, NM_006290, NM_178150, NM_012177, NM_004793, NM_006397, NM_001040876, NM_019108, NM_001144, NM_015435, NM_016297, NM_005908, NM_023010, NM_005789, NM_023011, NM_002791, NM_013282, NM_006704, NM_022079, NM_002947, NM_002946, NM_014871, NM_002945, NM_005647, NM_003407, NM_016237, NM_003183, NM_014762, NM_012308, NM_003877, NM_016617, NM_014311, NM_002808, NM_002809, NM_014819, NM_003925, NM_080599, NM_014814, NM_002804, NM_002800, NM_014110, NM_000123, NM_002801, NM_021633, NM_015057, NM_018561, NM_004725, NM_004628, NM_015176, NM_080632, NM_005101, NM_024646, NM_016172, NM_014674, NM_145813, NM_012199, NM_012215, NM_001786, NM_139286, NM_183419, NM_015276, NM_003299, NM_002818, NM_025133, NM_012486, NM_002816, NM_003819, NM_024955, NM_002810, NM_020765, NM_080687, NM_003955, NM_002812, NM_004935, NM_002358, NM_001375, NM_031299, NM_178270, NM_024602, NM_007362, NM_015029, NM_152132, NM_001109662, NM_006462, NM_013316, NM_000527, NM_174898, NM_015542, NM_004388, NM_006263, NM_178588, NM_025241, NM_199129, NM_020799, NM_031369, NM_181803, NM_016058, NM_194260, NM_005805, NM_014176, NM_031229, NM_018445, NM_024087, NM_145032, NM_018941, NM_022818, NM_152550, NM_031966, NM_181800	1.30	0.007990546
**mitosis**	3.59	NM_018454, NM_000601, NM_001255, NM_017518, NM_182620, NM_001013437, NM_018101, NM_001010932, NM_006341, NM_178014, NM_080668, NM_006530, NM_020242, NM_145060, NM_015329, NM_145061, NM_182513, NM_002497, NM_001269, NM_013299, NM_007044, NM_001168, NM_006325, NM_016343, NM_017785, NM_017668, NM_024094, NM_001007793, NM_006400, NM_004358, NM_018715, NM_004217, NM_004219, NM_001002800, NM_003707, NM_007317, NM_181042, NM_001012413, NM_030919, NM_000430, NM_014750, NM_018685, NM_003903, NM_138443, NM_024333, NM_012177, NM_001040694, NM_006396, NM_017760, NM_004701, NM_004336, NM_006086, NM_016195, NM_006600, NM_004856, NM_006704, NM_003390, NM_016237, NM_012291, NM_015261, NM_198436, NM_198434, NM_018944, NM_018097, NM_022346, NM_001008938, NM_018492, NM_199246, NM_001005413, NM_014865, NM_005445, NM_003920, NM_004725, NM_002956, NM_001789, NM_024057, NM_001786, NM_001761, NM_014303, NM_139286, NM_004523, NM_002263, NM_003914, NM_001813, NM_001790, NM_018136, NM_001011699, NM_018131, NM_012112, NM_002358, NM_031299, NM_014708, NM_015169, NM_001042550, NM_007221, NM_022809, NM_145697, NM_001013836, NM_006461, NM_015282, NM_031217, NM_181716, NM_138484, NM_024808, NM_005030, NM_182687, NM_152562, NM_181803, NM_006101, NM_006845, NM_194260, NM_025180, NM_031423, NM_017858, NM_033417, NM_181800, NM_031966	2.99	1.07E-26
**nuclear division**	3.59	NM_018454, NM_000601, NM_001255, NM_017518, NM_182620, NM_001013437, NM_018101, NM_001010932, NM_006341, NM_178014, NM_080668, NM_006530, NM_020242, NM_145060, NM_015329, NM_145061, NM_182513, NM_002497, NM_001269, NM_013299, NM_007044, NM_001168, NM_006325, NM_016343, NM_017785, NM_017668, NM_024094, NM_001007793, NM_006400, NM_004358, NM_018715, NM_004217, NM_004219, NM_001002800, NM_003707, NM_007317, NM_181042, NM_001012413, NM_030919, NM_000430, NM_014750, NM_018685, NM_003903, NM_138443, NM_024333, NM_012177, NM_001040694, NM_006396, NM_017760, NM_004701, NM_004336, NM_006086, NM_016195, NM_006600, NM_004856, NM_006704, NM_003390, NM_016237, NM_012291, NM_015261, NM_198436, NM_198434, NM_018944, NM_018097, NM_022346, NM_001008938, NM_018492, NM_199246, NM_001005413, NM_014865, NM_005445, NM_003920, NM_004725, NM_002956, NM_001789, NM_024057, NM_001786, NM_001761, NM_014303, NM_139286, NM_004523, NM_002263, NM_003914, NM_001813, NM_001790, NM_018136, NM_001011699, NM_018131, NM_012112, NM_002358, NM_031299, NM_014708, NM_015169, NM_001042550, NM_007221, NM_022809, NM_145697, NM_001013836, NM_006461, NM_015282, NM_031217, NM_181716, NM_138484, NM_024808, NM_005030, NM_182687, NM_152562, NM_181803, NM_006101, NM_006845, NM_194260, NM_025180, NM_031423, NM_017858, NM_033417, NM_181800, NM_031966	2.99	1.07E-26
**organelle fission**	3.63	NM_018454, NM_000601, NM_001255, NM_017518, NM_182620, NM_001013437, NM_018101, NM_001010932, NM_006341, NM_178014, NM_080668, NM_006530, NM_020242, NM_145060, NM_015329, NM_145061, NM_182513, NM_002497, NM_001269, NM_013299, NM_007044, NM_001168, NM_006325, NM_016343, NM_017785, NM_017668, NM_024094, NM_001007793, NM_006400, NM_004358, NM_018715, NM_024544, NM_004217, NM_004219, NM_001002800, NM_003707, NM_007317, NM_181042, NM_001012413, NM_030919, NM_000430, NM_014750, NM_018685, NM_003903, NM_138443, NM_024333, NM_012177, NM_001040694, NM_006396, NM_017760, NM_004701, NM_004336, NM_006086, NM_016195, NM_006600, NM_004856, NM_006704, NM_003390, NM_016237, NM_012291, NM_015261, NM_198436, NM_198434, NM_018944, NM_018097, NM_022346, NM_001008938, NM_018492, NM_199246, NM_001005413, NM_014865, NM_005445, NM_003920, NM_004725, NM_002956, NM_001789, NM_024057, NM_001786, NM_001761, NM_014303, NM_139286, NM_004523, NM_002263, NM_003914, NM_001813, NM_001790, NM_018136, NM_001011699, NM_018131, NM_012112, NM_002358, NM_031299, NM_014708, NM_015169, NM_001042550, NM_007221, NM_022809, NM_145697, NM_001013836, NM_006461, NM_015282, NM_031217, NM_181716, NM_138484, NM_024808, NM_005030, NM_182687, NM_152562, NM_181803, NM_006101, NM_006845, NM_194260, NM_025180, NM_031423, NM_017858, NM_033417, NM_181800, NM_031966	2.90	1.27E-25
**protein complex assembly**	3.73	NM_000165, NM_004781, NM_002014, NM_002293, NM_000801, NM_004355, NM_173515, NM_173039, NM_018310, NM_018087, NM_178014, NM_020243, NM_005483, NM_004865, NM_000319, NM_006009, NM_002495, NM_015135, NM_004774, NM_001079866, NM_001024465, NM_006473, NM_001070, NM_016447, NM_000377, NM_016343, NM_005348, NM_054014, NM_014166, NM_014165, NM_016338, NM_001042546, NM_005138, NM_001080975, NM_001902, NM_000249, NM_004140, NM_005359, NM_001025159, NM_032390, NM_016437, NM_001667, NM_032704, NM_000101, NM_018685, NM_002267, NM_015380, NM_182962, NM_001097577, NM_012177, NM_004793, NM_006000, NM_001359, NM_080651, NM_000057, NM_005658, NM_002086, NM_004343, NM_001144, NM_004029, NM_006087, NM_006086, NM_006088, NM_006082, NM_012426, NM_014225, NM_181598, NM_004069, NM_005644, NM_024658, NM_003201, NM_002156, NM_014260, NM_014317, NM_001752, NM_001017980, NM_000024, NM_003826, NM_015465, NM_005441, NM_004726, NM_024120, NM_001033, NM_001629, NM_001813, NM_006232, NM_015459, NM_002154, NM_004935, NM_014708, NM_033091, NM_022745, NM_152854, NM_030752, NM_001025235, NM_004493, NM_199069, NM_004492, NM_001034, NM_001017963, NM_002567, NM_001014840, NM_000289, NM_007166, NM_032151, NM_004269, NM_021830, NM_003172, NM_001250, NM_006347, NM_004604, NM_032940, NM_014071, NM_001316, NM_178191, NM_198576, NM_004169, NM_004552, NM_001014838, NM_005119	1.35	0.020067662
**protein complex biogenesis**	3.73	NM_000165, NM_004781, NM_002014, NM_002293, NM_000801, NM_004355, NM_173515, NM_173039, NM_018310, NM_018087, NM_178014, NM_020243, NM_005483, NM_004865, NM_000319, NM_006009, NM_002495, NM_015135, NM_004774, NM_001079866, NM_001024465, NM_006473, NM_001070, NM_016447, NM_000377, NM_016343, NM_005348, NM_054014, NM_014166, NM_014165, NM_016338, NM_001042546, NM_005138, NM_001080975, NM_001902, NM_000249, NM_004140, NM_005359, NM_001025159, NM_032390, NM_016437, NM_001667, NM_032704, NM_000101, NM_018685, NM_002267, NM_015380, NM_182962, NM_001097577, NM_012177, NM_004793, NM_006000, NM_001359, NM_080651, NM_000057, NM_005658, NM_002086, NM_004343, NM_001144, NM_004029, NM_006087, NM_006086, NM_006088, NM_006082, NM_012426, NM_014225, NM_181598, NM_004069, NM_005644, NM_024658, NM_003201, NM_002156, NM_014260, NM_014317, NM_001752, NM_001017980, NM_000024, NM_003826, NM_015465, NM_005441, NM_004726, NM_024120, NM_001033, NM_001629, NM_001813, NM_006232, NM_015459, NM_002154, NM_004935, NM_014708, NM_033091, NM_022745, NM_152854, NM_030752, NM_001025235, NM_004493, NM_199069, NM_004492, NM_001034, NM_001017963, NM_002567, NM_001014840, NM_000289, NM_007166, NM_032151, NM_004269, NM_021830, NM_003172, NM_001250, NM_006347, NM_004604, NM_032940, NM_014071, NM_001316, NM_178191, NM_198576, NM_004169, NM_004552, NM_001014838, NM_005119	1.35	0.020067662
**RNA splicing**	3.03	NM_005008, NM_138558, NM_006109, NM_031472, NM_025265, NM_001033521, NM_006938, NM_003017, NM_003016, NM_001031732, NM_005826, NM_015721, NM_006328, NM_002715, NM_006925, NM_018077, NM_032102, NM_015629, NM_031203, NM_005839, NM_004719, NM_014740, NM_002138, NM_014884, NM_002896, NM_022157, NM_003096, NM_003095, NM_003092, NM_001358, NM_001357, NM_002687, NM_001039619, NM_005791, NM_031263, NM_016424, NM_004501, NM_016199, NM_003089, NM_005850, NM_023010, NM_001293, NM_007279, NM_014225, NM_012426, NM_004596, NM_006831, NM_017612, NM_006445, NM_024102, NM_014110, NM_032864, NM_032361, NM_018047, NM_001077442, NM_015465, NM_080632, NM_032177, NM_001042581, NM_194247, NM_004247, NM_007006, NM_003587, NM_001042588, NM_014502, NM_005968, NM_006232, NM_003594, NM_006372, NM_024831, NM_001031684, NM_006857, NM_015496, NM_012321, NM_007362, NM_152858, NM_001324, NM_004559, NM_014281, NM_001325, NM_003946, NM_022805, NM_001033505, NM_021177, NM_004697, NM_031369, NM_184234, NM_003769, NM_005804, NM_021190, NM_004960, NM_006347, NM_032940, NM_001078166, NM_024571, NM_004814, NM_020230, NM_014829, NM_001017392, NM_004818, NM_032758	1.96	1.11E-08

KM-H2/L-428				
Process	% Genes	Genes	Fold Enrichment	Benjamini
cell cycle	6.69	NM_001259, NM_052988, NM_001018160, NM_018454, NM_002208, NM_016556, NM_000700, NM_017518, NM_006480, NM_018101, NM_206861, NM_002094, NM_002093, NM_005915, NM_002878, NM_006342, NM_002876, NM_000075, NM_170713, NM_000785, NM_021009, NM_020739, NM_022034, NM_004159, NM_015329, NM_182513, NM_002497, NM_006027, NM_002911, NM_032637, NM_006472, NM_001070, NM_001003786, NM_016343, NM_017668, NM_024094, NM_172166, NM_001007793, NM_004091, NM_078468, NM_001078645, NM_078469, NM_003333, NM_004217, NM_002710, NM_004219, NM_006997, NM_006503, NM_152896, NM_003707, NM_181042, NM_003858, NM_013290, NM_000051, NM_004516, NM_015255, NM_000430, NM_002473, NM_014750, NM_031459, NM_058195, NM_003903, NM_024333, NM_002788, NM_002787, NM_203401, NM_016567, NM_018667, NM_005556, NM_182776, NM_178012, NM_016426, NM_004343, NM_013374, NM_019063, NM_206876, NM_004701, NM_017815, NM_004336, NM_021145, NM_004642, NM_006600, NM_004450, NM_005789, NM_005563, NM_002791, NM_001042426, NM_004237, NM_006640, NM_003183, NM_014762, NM_033028, NM_014454, NM_018410, NM_014060, NM_015374, NM_005504, NM_182666, NM_002809, NM_018492, NM_014814, NM_005197, NM_199246, NM_001005413, NM_005652, NM_018353, NM_014397, NM_079421, NM_007111, NM_024057, NM_001786, NM_002048, NM_013345, NM_014303, NM_001760, NM_002263, NM_000234, NM_018223, NM_003591, NM_018031, NM_002815, NM_002816, NM_078626, NM_014874, NM_003504, NM_015169, NM_198971, NM_004417, NM_005132, NM_025193, NM_005334, NM_152132, NM_002754, NM_002755, NM_181716, NM_002226, NM_018234, NM_199122, NM_004943, NM_006559, NM_002441, NM_181803, NM_002386, NM_007234, NM_025180, NM_002746, NM_017489, NM_017853, NM_007018	1.56	2.27E-05
cell cycle phase	3.94	NM_052988, NM_001259, NM_018454, NM_016556, NM_017518, NM_003183, NM_006480, NM_018101, NM_206861, NM_015374, NM_002094, NM_005504, NM_002878, NM_006342, NM_000075, NM_018492, NM_002876, NM_005197, NM_199246, NM_001005413, NM_018353, NM_015329, NM_014397, NM_182513, NM_002497, NM_006027, NM_079421, NM_001070, NM_032637, NM_024057, NM_001786, NM_016343, NM_013345, NM_017668, NM_014303, NM_024094, NM_002263, NM_018223, NM_003591, NM_172166, NM_001007793, NM_001078645, NM_078626, NM_004217, NM_015169, NM_004219, NM_198971, NM_005132, NM_006997, NM_003707, NM_025193, NM_181042, NM_003858, NM_002755, NM_181716, NM_013290, NM_000051, NM_004516, NM_015255, NM_002473, NM_000430, NM_014750, NM_199122, NM_058195, NM_004943, NM_003903, NM_006559, NM_024333, NM_203401, NM_002441, NM_181803, NM_002386, NM_005556, NM_007234, NM_025180, NM_016426, NM_178012, NM_019063, NM_017815, NM_004701, NM_206876, NM_017489, NM_017853, NM_004336, NM_006600, NM_004642, NM_005563, NM_004237	1.72	1.62E-04
cell cycle process	5.13	NM_001259, NM_052988, NM_018454, NM_016556, NM_017518, NM_006480, NM_018101, NM_206861, NM_002094, NM_002093, NM_002878, NM_006342, NM_000075, NM_002876, NM_170713, NM_000785, NM_021009, NM_004159, NM_015329, NM_182513, NM_002497, NM_006027, NM_032637, NM_001070, NM_016343, NM_017668, NM_024094, NM_172166, NM_001007793, NM_001078645, NM_003333, NM_004217, NM_004219, NM_006997, NM_006503, NM_003707, NM_181042, NM_003858, NM_013290, NM_000051, NM_004516, NM_015255, NM_000430, NM_002473, NM_014750, NM_058195, NM_031459, NM_003903, NM_024333, NM_002788, NM_002787, NM_203401, NM_005556, NM_016426, NM_178012, NM_019063, NM_004343, NM_206876, NM_004701, NM_017815, NM_004336, NM_006600, NM_004642, NM_005789, NM_005563, NM_002791, NM_001042426, NM_004237, NM_003183, NM_014762, NM_033028, NM_014454, NM_015374, NM_005504, NM_182666, NM_002809, NM_018492, NM_014814, NM_005197, NM_199246, NM_001005413, NM_018353, NM_014397, NM_079421, NM_024057, NM_001786, NM_002048, NM_013345, NM_014303, NM_002263, NM_018223, NM_003591, NM_018031, NM_002815, NM_002816, NM_078626, NM_014874, NM_015169, NM_198971, NM_005132, NM_025193, NM_152132, NM_002755, NM_181716, NM_199122, NM_004943, NM_006559, NM_002441, NM_181803, NM_002386, NM_007234, NM_025180, NM_017489, NM_017853	1.64	5.93E-05
cell death	5.84	NM_001018160, NM_024900, NM_000600, NM_015331, NM_002094, NM_021009, NM_001165, NM_005605, NM_004862, NM_020247, NM_030974, NM_004208, NM_001079864, NM_001540, NM_001024465, NM_001024466, NM_198057, NM_020529, NM_004091, NM_133328, NM_007350, NM_003334, NM_003333, NM_016553, NM_016077, NM_004765, NM_001008540, NM_025137, NM_007315, NM_016640, NM_004331, NM_003467, NM_021127, NM_000595, NM_000391, NM_000051, NM_000594, NM_058195, NM_004840, NM_004708, NM_006986, NM_032983, NM_006290, NM_016222, NM_057178, NM_001092, NM_005165, NM_033657, NM_152240, NM_005658, NM_001040021, NM_013374, NM_001033030, NM_005789, NM_016399, NM_145074, NM_024755, NM_004435, NM_024622, NM_201559, NM_001008213, NM_002157, NM_022767, NM_002156, NM_014762, NM_003404, NM_000945, NM_017852, NM_016498, NM_019555, NM_003824, NM_015367, NM_003583, NM_024948, NM_032667, NM_003680, NM_014397, NM_022873, NM_004052, NM_133171, NM_079421, NM_020396, NM_145813, NM_033015, NM_201430, NM_002048, NM_012215, NM_025126, NM_003810, NM_015356, NM_004394, NM_006427, NM_014452, NM_175069, NM_014800, NM_001375, NM_019887, NM_001035235, NM_002654, NM_015675, NM_001735, NM_016068, NM_182471, NM_182470, NM_004089, NM_020919, NM_003897, NM_013247, NM_003374, NM_033007, NM_012329, NM_001636, NM_153719, NM_002228, NM_004281, NM_018234, NM_003946, NM_003806, NM_003804, NM_004282, NM_199122, NM_181676, NM_001005332, NM_213566, NM_003768, NM_004401, NM_003217, NM_198282, NM_031892, NM_017489, NM_018941, NM_199139, NM_015913	1.47	0.00126469
cellular macromolecular complex assembly	3.32	NM_002014, NM_024658, NM_001024959, NM_002156, NM_003201, NM_000801, NM_014260, NM_018410, NM_001303, NM_032025, NM_003541, NM_018139, NM_000024, NM_024102, NM_020243, NM_033445, NM_006938, NM_080593, NM_005015, NM_002495, NM_001079866, NM_032177, NM_001042581, NM_006026, NM_001070, NM_015721, NM_024120, NM_015171, NM_001017990, NM_000377, NM_002107, NM_016343, NM_001042588, NM_001017991, NM_005348, NM_054014, NM_177925, NM_003542, NM_014165, NM_182691, NM_006766, NM_003750, NM_003548, NM_004939, NM_002154, NM_016101, NM_004719, NM_030752, NM_007362, NM_181716, NM_016437, NM_001017963, NM_000101, NM_003512, NM_003096, NM_207312, NM_005324, NM_003095, NM_004893, NM_015380, NM_199202, NM_003172, NM_006001, NM_002386, NM_001013699, NM_001039619, NM_178012, NM_001078166, NM_004343, NM_017953, NM_001040874, NM_005319, NM_018154, NM_006087, NM_004552, NM_001042426	1.89	5.65E-05
cellular macromolecular complex subunit organization	3.51	NM_002014, NM_024658, NM_001024959, NM_000801, NM_002156, NM_003201, NM_014260, NM_018410, NM_001303, NM_032025, NM_003541, NM_018139, NM_000024, NM_024102, NM_020243, NM_033445, NM_006938, NM_080593, NM_005015, NM_002495, NM_001079866, NM_032177, NM_001042581, NM_006026, NM_001070, NM_015721, NM_024120, NM_015171, NM_001017990, NM_000377, NM_002107, NM_016343, NM_001042588, NM_001017991, NM_005348, NM_054014, NM_177925, NM_003542, NM_014165, NM_182691, NM_007192, NM_006766, NM_003750, NM_003548, NM_004939, NM_002154, NM_016101, NM_004719, NM_030752, NM_007362, NM_181716, NM_016437, NM_001017963, NM_000101, NM_003512, NM_003096, NM_207312, NM_005324, NM_003095, NM_004893, NM_015380, NM_199202, NM_003172, NM_203401, NM_006001, NM_002386, NM_001013699, NM_001039619, NM_019041, NM_178012, NM_001078166, NM_004343, NM_017953, NM_001040874, NM_005319, NM_018154, NM_006087, NM_004552, NM_199177, NM_005563, NM_001042426	1.78	1.79E-04
cellular response to stress	4.84	NM_001018160, NM_003146, NM_024295, NM_017518, NM_001014436, NM_001923, NM_001033002, NM_015713, NM_002093, NM_002878, NM_002876, NM_004208, NM_005485, NM_003348, NM_001024465, NM_001024466, NM_014489, NM_006027, NM_002911, NM_004905, NM_152221, NM_005347, NM_002916, NM_080876, NM_153824, NM_001039847, NM_172166, NM_002412, NM_078468, NM_001527, NM_078469, NM_001042697, NM_181696, NM_004219, NM_001990, NM_021141, NM_030811, NM_002528, NM_198948, NM_001127218, NM_006793, NM_000051, NM_000594, NM_001048172, NM_003907, NM_006812, NM_004840, NM_016567, NM_004793, NM_001605, NM_001031716, NM_182776, NM_001706, NM_152240, NM_016426, NM_005066, NM_206876, NM_006304, NM_004454, NM_004029, NM_020135, NM_016399, NM_016290, NM_004237, NM_002350, NM_002947, NM_198336, NM_201559, NM_022767, NM_003392, NM_014454, NM_014060, NM_001618, NM_198954, NM_003583, NM_005197, NM_005652, NM_000123, NM_003211, NM_001033858, NM_002957, NM_001013703, NM_079421, NM_145813, NM_001786, NM_001003686, NM_000234, NM_001005291, NM_182908, NM_014502, NM_007192, NM_175069, NM_005395, NM_198971, NM_052966, NM_005531, NM_002228, NM_014481, NM_003806, NM_003804, NM_206839, NM_015920, NM_002441, NM_025180, NM_018445, NM_000486, NM_018248, NM_015913, NM_001029835	1.55	0.001029616
chromosome organization	3.89	NM_012461, NM_024900, NM_017575, NM_018454, NM_002634, NM_018410, NM_018101, NM_003541, NM_004739, NM_001618, NM_182666, NM_002876, NM_004964, NM_003070, NM_014292, NM_001005413, NM_033445, NM_005652, NM_003074, NM_017745, NM_003072, NM_003078, NM_004208, NM_001033858, NM_080593, NM_003348, NM_015329, NM_002497, NM_004052, NM_006026, NM_024644, NM_145813, NM_001017990, NM_002107, NM_016343, NM_031407, NM_024094, NM_001017991, NM_002263, NM_005189, NM_177925, NM_001039847, NM_003542, NM_172166, NM_001007793, NM_007192, NM_031488, NM_001527, NM_006766, NM_003548, NM_015169, NM_004219, NM_005132, NM_021078, NM_004755, NM_003707, NM_001990, NM_032590, NM_181042, NM_021141, NM_019071, NM_022743, NM_181716, NM_004456, NM_003512, NM_005324, NM_003655, NM_004893, NM_014750, NM_152998, NM_206839, NM_002931, NM_199141, NM_002441, NM_024665, NM_001079514, NM_001013699, NM_001039619, NM_005517, NM_001040874, NM_017489, NM_005319, NM_018154, NM_006601, NM_001042426, NM_183359, NM_016290, NM_001363	1.45	0.029213906
death	5.84	NM_001018160, NM_024900, NM_000600, NM_015331, NM_002094, NM_021009, NM_001165, NM_005605, NM_004862, NM_020247, NM_030974, NM_004208, NM_001079864, NM_001540, NM_001024465, NM_001024466, NM_198057, NM_020529, NM_004091, NM_133328, NM_007350, NM_003334, NM_003333, NM_016553, NM_016077, NM_004765, NM_001008540, NM_025137, NM_007315, NM_016640, NM_004331, NM_003467, NM_021127, NM_000595, NM_000391, NM_000051, NM_000594, NM_058195, NM_004840, NM_004708, NM_006986, NM_032983, NM_006290, NM_016222, NM_057178, NM_001092, NM_005165, NM_033657, NM_152240, NM_005658, NM_001040021, NM_013374, NM_001033030, NM_005789, NM_016399, NM_145074, NM_024755, NM_004435, NM_024622, NM_201559, NM_001008213, NM_002157, NM_022767, NM_002156, NM_014762, NM_003404, NM_000945, NM_017852, NM_016498, NM_019555, NM_003824, NM_015367, NM_003583, NM_024948, NM_032667, NM_003680, NM_014397, NM_022873, NM_004052, NM_133171, NM_079421, NM_020396, NM_145813, NM_033015, NM_201430, NM_002048, NM_012215, NM_025126, NM_003810, NM_015356, NM_004394, NM_006427, NM_014452, NM_175069, NM_014800, NM_001375, NM_019887, NM_001035235, NM_002654, NM_015675, NM_001735, NM_016068, NM_182471, NM_182470, NM_004089, NM_020919, NM_003897, NM_013247, NM_003374, NM_033007, NM_012329, NM_001636, NM_153719, NM_002228, NM_004281, NM_018234, NM_003946, NM_003806, NM_003804, NM_004282, NM_199122, NM_181676, NM_001005332, NM_213566, NM_003768, NM_004401, NM_003217, NM_198282, NM_031892, NM_017489, NM_018941, NM_199139, NM_015913	1.46	0.001564798
DNA metabolic process	4.89	NM_012461, NM_001018160, NM_003146, NM_018518, NM_016556, NM_152308, NM_017518, NM_001923, NM_018845, NM_001033002, NM_015713, NM_005915, NM_002878, NM_002876, NM_006190, NM_017660, NM_205843, NM_006739, NM_004208, NM_005485, NM_003348, NM_001024465, NM_001024466, NM_006027, NM_002911, NM_016343, NM_152221, NM_002916, NM_020661, NM_024094, NM_017676, NM_172166, NM_002412, NM_078468, NM_078469, NM_001042697, NM_003143, NM_004219, NM_003707, NM_001990, NM_021130, NM_021141, NM_002528, NM_021137, NM_198948, NM_001127218, NM_013290, NM_000051, NM_002897, NM_001048172, NM_002824, NM_003860, NM_058195, NM_016567, NM_004793, NM_005556, NM_006397, NM_001031716, NM_182776, NM_003258, NM_005066, NM_002936, NM_006304, NM_178863, NM_020135, NM_006601, NM_004642, NM_016290, NM_001363, NM_004237, NM_004435, NM_002947, NM_017575, NM_002634, NM_003201, NM_002156, NM_001618, NM_198954, NM_020201, NM_005652, NM_000123, NM_003211, NM_001033858, NM_004052, NM_079421, NM_145813, NM_001003686, NM_000234, NM_001953, NM_014502, NM_007192, NM_002553, NM_175069, NM_003504, NM_001375, NM_005395, NM_198971, NM_005132, NM_025193, NM_030755, NM_001034, NM_014481, NM_206839, NM_015920, NM_002441, NM_213566, NM_004401, NM_014374, NM_033222, NM_017489, NM_018248, NM_015913	1.75	8.34E-06
establishment of protein localization	5.79	NM_024295, NM_032520, NM_004355, NM_004910, NM_001033002, NM_006114, NM_002093, NM_001042535, NM_020243, NM_007342, NM_022340, NM_005488, NM_004206, NM_007347, NM_014488, NM_003135, NM_018261, NM_005829, NM_178326, NM_006327, NM_001003786, NM_014353, NM_016343, NM_020529, NM_006120, NM_016553, NM_000308, NM_000466, NM_207629, NM_002868, NM_003145, NM_003715, NM_152238, NM_001025159, NM_001136232, NM_017966, NM_001001433, NM_144636, NM_000390, NM_015254, NM_000594, NM_201434, NM_003134, NM_016208, NM_020750, NM_001565, NM_002473, NM_001283, NM_020857, NM_015638, NM_012414, NM_032985, NM_000253, NM_144497, NM_032511, NM_007065, NM_057178, NM_145648, NM_175624, NM_152244, NM_005796, NM_001706, NM_152240, NM_013374, NM_004343, NM_000428, NM_006809, NM_002738, NM_030805, NM_014044, NM_001970, NM_006803, NM_015509, NM_002350, NM_001034025, NM_015049, NM_014669, NM_015161, NM_153485, NM_001008213, NM_024658, NM_003404, NM_139279, NM_032970, NM_052948, NM_018144, NM_138798, NM_033542, NM_006573, NM_005638, NM_032177, NM_001042581, NM_016226, NM_006826, NM_003292, NM_015171, NM_024057, NM_001031677, NM_012215, NM_001042588, NM_003299, NM_003916, NM_003917, NM_014700, NM_014874, NM_015044, NM_003794, NM_198971, NM_001007067, NM_004085, NM_003164, NM_013312, NM_001013839, NM_001014451, NM_153719, NM_002228, NM_198883, NM_012479, NM_000288, NM_014177, NM_013321, NM_004603, NM_013322, NM_006947, NM_018445, NM_014169	1.36	0.020012276
intracellular transport	5.27	NM_024295, NM_032520, NM_004355, NM_001487, NM_001033002, NM_006114, NM_002093, NM_001687, NM_001042535, NM_020243, NM_007342, NM_022340, NM_015327, NM_005488, NM_007347, NM_004206, NM_003135, NM_005829, NM_002911, NM_006327, NM_001003786, NM_000377, NM_020529, NM_017668, NM_005348, NM_017983, NM_000308, NM_000466, NM_207629, NM_003145, NM_003715, NM_001025159, NM_001136232, NM_001001433, NM_006795, NM_000390, NM_015254, NM_000594, NM_003134, NM_020750, NM_000430, NM_002473, NM_001283, NM_015638, NM_012414, NM_032985, NM_144497, NM_032511, NM_007065, NM_057178, NM_175624, NM_005796, NM_001706, NM_004343, NM_000428, NM_030808, NM_006809, NM_030805, NM_006803, NM_001970, NM_001034025, NM_015049, NM_017575, NM_015161, NM_153485, NM_001008213, NM_024658, NM_033028, NM_003404, NM_032970, NM_080599, NM_000024, NM_080598, NM_018144, NM_153201, NM_005638, NM_032177, NM_001042581, NM_004052, NM_006826, NM_003292, NM_015171, NM_012215, NM_001042588, NM_003916, NM_003917, NM_015459, NM_014874, NM_015044, NM_001007067, NM_004085, NM_001081491, NM_003164, NM_007362, NM_020919, NM_013312, NM_030755, NM_002755, NM_001636, NM_001014451, NM_002228, NM_001017963, NM_198883, NM_012479, NM_000288, NM_001011658, NM_000146, NM_006362, NM_012140, NM_004603, NM_005895, NM_006947, NM_001744, NM_018445, NM_002032, NM_170784	1.45	0.004273962
macromolecular complex assembly	5.41	NM_002014, NM_000801, NM_004355, NM_003541, NM_014298, NM_001165, NM_020243, NM_001261, NM_033445, NM_006938, NM_005015, NM_002495, NM_001079866, NM_001024465, NM_001024466, NM_006026, NM_001070, NM_015721, NM_000377, NM_002107, NM_016343, NM_006120, NM_005348, NM_054014, NM_177925, NM_014166, NM_003542, NM_014165, NM_182691, NM_004091, NM_003750, NM_003548, NM_003648, NM_001902, NM_004719, NM_001025159, NM_032390, NM_016437, NM_212461, NM_000101, NM_003096, NM_003512, NM_207312, NM_003095, NM_021128, NM_015638, NM_015380, NM_199202, NM_006001, NM_021974, NM_004793, NM_001013699, NM_005165, NM_001039619, NM_005658, NM_178012, NM_002086, NM_004446, NM_004343, NM_017953, NM_001040874, NM_001154, NM_004029, NM_006087, NM_001042426, NM_012426, NM_024658, NM_001024959, NM_006640, NM_003201, NM_002156, NM_003404, NM_014260, NM_001303, NM_014317, NM_018410, NM_032025, NM_018139, NM_003824, NM_000026, NM_000024, NM_024102, NM_080593, NM_004726, NM_001382, NM_032177, NM_001042581, NM_024120, NM_015171, NM_001017990, NM_001042588, NM_001629, NM_001017991, NM_001025243, NM_006766, NM_004939, NM_015459, NM_002154, NM_016101, NM_033091, NM_022745, NM_030752, NM_007362, NM_001025235, NM_004493, NM_002755, NM_181716, NM_001034, NM_001017963, NM_002567, NM_005324, NM_004893, NM_000289, NM_003172, NM_002386, NM_001078166, NM_017489, NM_178191, NM_005319, NM_018154, NM_004552, NM_000878	1.47	0.002203183
macromolecular complex subunit organization	5.70	NM_002014, NM_000801, NM_004355, NM_003541, NM_014298, NM_001165, NM_020243, NM_033445, NM_001261, NM_006938, NM_005015, NM_002495, NM_001079866, NM_001024465, NM_001024466, NM_006026, NM_001070, NM_015721, NM_000377, NM_002107, NM_016343, NM_006120, NM_005348, NM_054014, NM_177925, NM_014166, NM_003542, NM_014165, NM_182691, NM_004091, NM_003750, NM_003548, NM_003648, NM_207629, NM_001902, NM_004719, NM_001025159, NM_032390, NM_016437, NM_212461, NM_000101, NM_003096, NM_003512, NM_207312, NM_003095, NM_021128, NM_015638, NM_015380, NM_199202, NM_203401, NM_006001, NM_021974, NM_004793, NM_001013699, NM_005165, NM_001039619, NM_019041, NM_005658, NM_178012, NM_002086, NM_004446, NM_004343, NM_001040874, NM_017953, NM_001154, NM_004029, NM_006087, NM_199177, NM_005563, NM_001042426, NM_012426, NM_024658, NM_001024959, NM_006640, NM_003201, NM_002156, NM_003404, NM_014260, NM_001303, NM_014317, NM_018410, NM_032025, NM_018139, NM_005505, NM_003824, NM_000026, NM_000024, NM_024102, NM_080593, NM_004726, NM_001382, NM_032177, NM_001042581, NM_024120, NM_001017990, NM_015171, NM_001042588, NM_001629, NM_001017991, NM_001025243, NM_007192, NM_006766, NM_004939, NM_015459, NM_002154, NM_016101, NM_033091, NM_022745, NM_030752, NM_007362, NM_001025235, NM_004493, NM_002755, NM_181716, NM_001034, NM_001017963, NM_002567, NM_005324, NM_004893, NM_000289, NM_003172, NM_002386, NM_001078166, NM_017489, NM_005319, NM_178191, NM_018154, NM_004552, NM_000878	1.45	0.002345707
mitotic cell cycle	3.80	NM_052988, NM_001259, NM_018454, NM_017518, NM_003183, NM_006480, NM_018101, NM_015374, NM_002094, NM_005504, NM_182666, NM_002809, NM_000075, NM_018492, NM_014814, NM_005197, NM_021009, NM_199246, NM_001005413, NM_018353, NM_004159, NM_015329, NM_014397, NM_182513, NM_002497, NM_079421, NM_032637, NM_024057, NM_001786, NM_016343, NM_017668, NM_014303, NM_013345, NM_024094, NM_002263, NM_018223, NM_003591, NM_001007793, NM_001078645, NM_002815, NM_002816, NM_003333, NM_078626, NM_004217, NM_015169, NM_004219, NM_198971, NM_006503, NM_003707, NM_152132, NM_181042, NM_003858, NM_002755, NM_181716, NM_000430, NM_014750, NM_199122, NM_058195, NM_003903, NM_006559, NM_002788, NM_024333, NM_002787, NM_203401, NM_181803, NM_002386, NM_007234, NM_025180, NM_016426, NM_178012, NM_019063, NM_017815, NM_004701, NM_206876, NM_017489, NM_017853, NM_004336, NM_006600, NM_004642, NM_005789, NM_005563, NM_002791, NM_001042426	1.86	2.03E-05

**mRNA metabolic process**	3.65	NM_173797, NM_017575, NM_006527, NM_014871, NM_017612, NM_012207, NM_017892, NM_006112, NM_002094, NM_080599, NM_080598, NM_024102, NM_025265, NM_032864, NM_006938, NM_015327, NM_006196, NM_001077442, NM_080632, NM_032177, NM_001042581, NM_194247, NM_002911, NM_015721, NM_015360, NM_001042588, NM_020661, NM_203456, NM_018077, NM_014502, NM_182691, NM_203457, NM_031844, NM_004396, NM_004939, NM_006372, NM_003142, NM_004719, NM_006857, NM_007362, NM_002137, NM_052965, NM_004559, NM_002138, NM_001325, NM_014884, NM_152624, NM_003096, NM_016306, NM_003946, NM_003095, NM_032881, NM_003092, NM_001033505, NM_016207, NM_021128, NM_007359, NM_006558, NM_021177, NM_032905, NM_004698, NM_006559, NM_031369, NM_021931, NM_016222, NM_001358, NM_021974, NM_021190, NM_002685, NM_001039619, NM_001078166, NM_031263, NM_005066, NM_019108, NM_003089, NM_017853, NM_004814, NM_023010, NM_014829, NM_004593, NM_001017392, NM_001005335, NM_198220, NM_012426	1.79	1.06E-04
**mRNA processing**	3.13	NM_173797, NM_006527, NM_017612, NM_012207, NM_017892, NM_006112, NM_080598, NM_024102, NM_025265, NM_032864, NM_006938, NM_006196, NM_001077442, NM_080632, NM_032177, NM_001042581, NM_194247, NM_015721, NM_015360, NM_001042588, NM_020661, NM_203456, NM_018077, NM_014502, NM_182691, NM_203457, NM_031844, NM_004396, NM_004939, NM_006372, NM_004719, NM_006857, NM_007362, NM_002137, NM_052965, NM_004559, NM_002138, NM_001325, NM_014884, NM_003096, NM_003946, NM_003095, NM_032881, NM_003092, NM_001033505, NM_016207, NM_021128, NM_006558, NM_021177, NM_032905, NM_007359, NM_004698, NM_006559, NM_031369, NM_021931, NM_016222, NM_001358, NM_021974, NM_021190, NM_001039619, NM_001078166, NM_031263, NM_005066, NM_003089, NM_017853, NM_004814, NM_023010, NM_014829, NM_004593, NM_001017392, NM_001005335, NM_198220, NM_012426	1.76	8.58E-04
**programmed cell death**	5.13	NM_001018160, NM_024900, NM_000600, NM_015331, NM_002094, NM_021009, NM_001165, NM_005605, NM_004862, NM_030974, NM_001079864, NM_004208, NM_001024465, NM_001024466, NM_198057, NM_020529, NM_004091, NM_133328, NM_007350, NM_003333, NM_016077, NM_004765, NM_001008540, NM_007315, NM_016640, NM_004331, NM_003467, NM_021127, NM_000595, NM_000051, NM_000594, NM_058195, NM_004840, NM_004708, NM_006986, NM_032983, NM_006290, NM_057178, NM_016222, NM_001092, NM_005165, NM_033657, NM_152240, NM_005658, NM_001040021, NM_013374, NM_001033030, NM_005789, NM_016399, NM_145074, NM_024755, NM_004435, NM_024622, NM_201559, NM_002157, NM_002156, NM_022767, NM_014762, NM_003404, NM_000945, NM_016498, NM_017852, NM_019555, NM_003824, NM_015367, NM_003583, NM_024948, NM_003680, NM_014397, NM_004052, NM_133171, NM_022873, NM_079421, NM_020396, NM_145813, NM_033015, NM_201430, NM_002048, NM_025126, NM_003810, NM_015356, NM_004394, NM_006427, NM_014452, NM_014800, NM_001375, NM_019887, NM_001035235, NM_002654, NM_015675, NM_016068, NM_182471, NM_182470, NM_004089, NM_003897, NM_013247, NM_003374, NM_033007, NM_001636, NM_002228, NM_004281, NM_018234, NM_003946, NM_003806, NM_003804, NM_004282, NM_199122, NM_001005332, NM_213566, NM_003768, NM_004401, NM_003217, NM_198282, NM_031892, NM_017489, NM_199139, NM_015913	1.52	0.001254259
**protein localization**	6.69	NM_012461, NM_024295, NM_031885, NM_032520, NM_004355, NM_004910, NM_001033002, NM_006114, NM_002093, NM_006342, NM_001042535, NM_020243, NM_007342, NM_022340, NM_005488, NM_005015, NM_004206, NM_007347, NM_003135, NM_014488, NM_018261, NM_021992, NM_005829, NM_178326, NM_006327, NM_001003786, NM_014353, NM_016343, NM_020529, NM_006120, NM_004356, NM_016553, NM_000308, NM_000466, NM_207629, NM_002868, NM_004217, NM_003145, NM_003715, NM_152238, NM_001025159, NM_001136232, NM_017966, NM_001001433, NM_144636, NM_032387, NM_000390, NM_015254, NM_000594, NM_201434, NM_003134, NM_016208, NM_020750, NM_001565, NM_002473, NM_001283, NM_020857, NM_015638, NM_006812, NM_012414, NM_032985, NM_000253, NM_144497, NM_032511, NM_007065, NM_057178, NM_145648, NM_152244, NM_175624, NM_005796, NM_001706, NM_152240, NM_004343, NM_013374, NM_000428, NM_006809, NM_002738, NM_030805, NM_014044, NM_001970, NM_006803, NM_015509, NM_002350, NM_001034025, NM_015049, NM_014669, NM_015161, NM_153485, NM_001008213, NM_024658, NM_014762, NM_003404, NM_033028, NM_015373, NM_139279, NM_032970, NM_006156, NM_052948, NM_018144, NM_005652, NM_138798, NM_033542, NM_006573, NM_005638, NM_032177, NM_001042581, NM_021955, NM_016226, NM_006826, NM_001039802, NM_003292, NM_015171, NM_024057, NM_001031677, NM_012215, NM_001042588, NM_003299, NM_003916, NM_003917, NM_014700, NM_014874, NM_015044, NM_003794, NM_198971, NM_001001716, NM_001007067, NM_004085, NM_003164, NM_020919, NM_013312, NM_001013839, NM_001014451, NM_153719, NM_002228, NM_198883, NM_012479, NM_000288, NM_014177, NM_013321, NM_004603, NM_013322, NM_003565, NM_006947, NM_018445, NM_014169	1.37	0.005604536
**protein transport**	5.74	NM_024295, NM_032520, NM_004355, NM_004910, NM_001033002, NM_006114, NM_002093, NM_001042535, NM_020243, NM_007342, NM_022340, NM_005488, NM_004206, NM_007347, NM_014488, NM_003135, NM_018261, NM_005829, NM_178326, NM_006327, NM_001003786, NM_014353, NM_016343, NM_020529, NM_006120, NM_016553, NM_000308, NM_000466, NM_207629, NM_002868, NM_003145, NM_003715, NM_152238, NM_001025159, NM_001136232, NM_017966, NM_001001433, NM_144636, NM_000390, NM_015254, NM_000594, NM_201434, NM_003134, NM_016208, NM_020750, NM_001565, NM_002473, NM_001283, NM_020857, NM_015638, NM_012414, NM_032985, NM_000253, NM_144497, NM_032511, NM_007065, NM_057178, NM_145648, NM_175624, NM_152244, NM_005796, NM_001706, NM_152240, NM_013374, NM_004343, NM_000428, NM_006809, NM_002738, NM_030805, NM_014044, NM_001970, NM_006803, NM_015509, NM_002350, NM_001034025, NM_015049, NM_014669, NM_015161, NM_153485, NM_001008213, NM_024658, NM_003404, NM_139279, NM_032970, NM_052948, NM_018144, NM_138798, NM_033542, NM_006573, NM_005638, NM_032177, NM_001042581, NM_016226, NM_006826, NM_003292, NM_015171, NM_024057, NM_001031677, NM_012215, NM_001042588, NM_003299, NM_003916, NM_003917, NM_014700, NM_014874, NM_015044, NM_003794, NM_001007067, NM_004085, NM_003164, NM_013312, NM_001013839, NM_001014451, NM_153719, NM_002228, NM_198883, NM_012479, NM_000288, NM_014177, NM_013321, NM_004603, NM_013322, NM_006947, NM_018445, NM_014169	1.36	0.020206876
**regulation of apoptosis**	6.79	NM_001018160, NM_000600, NM_015331, NM_000700, NM_016558, NM_004355, NM_015713, NM_002094, NM_002093, NM_198175, NM_004964, NM_021009, NM_001165, NM_005605, NM_004208, NM_001079864, NM_001540, NM_001024465, NM_001024466, NM_014489, NM_006472, NM_032637, NM_020529, NM_005347, NM_002412, NM_133328, NM_007350, NM_003333, NM_016553, NM_012448, NM_181696, NM_007315, NM_001025159, NM_019071, NM_021141, NM_001018677, NM_004331, NM_147173, NM_021127, NM_000595, NM_006793, NM_000051, NM_000594, NM_003655, NM_003327, NM_058195, NM_004840, NM_004708, NM_006986, NM_032983, NM_006290, NM_001092, NM_001605, NM_033657, NM_001706, NM_152240, NM_005658, NM_004343, NM_006708, NM_001154, NM_004994, NM_001033030, NM_005789, NM_016399, NM_001970, NM_145074, NM_001556, NM_001007277, NM_004435, NM_002634, NM_201559, NM_002157, NM_022767, NM_003183, NM_002156, NM_014762, NM_032494, NM_003404, NM_000945, NM_017852, NM_019555, NM_005507, NM_172014, NM_058246, NM_003824, NM_015367, NM_206955, NM_003583, NM_000024, NM_004309, NM_000123, NM_001001391, NM_006573, NM_002957, NM_153498, NM_016037, NM_022873, NM_004052, NM_079421, NM_020396, NM_145813, NM_033015, NM_003295, NM_001786, NM_002048, NM_003299, NM_003591, NM_003810, NM_182908, NM_000852, NM_001025243, NM_015356, NM_004394, NM_006427, NM_078626, NM_006378, NM_003690, NM_019887, NM_004417, NM_003897, NM_013247, NM_030755, NM_033007, NM_004379, NM_001731, NM_001014451, NM_005531, NM_153719, NM_002226, NM_002228, NM_004281, NM_003946, NM_013974, NM_003806, NM_003804, NM_015920, NM_003807, NM_002167, NM_001005332, NM_213566, NM_003768, NM_004401, NM_003217, NM_001189, NM_018441, NM_002027, NM_018445, NM_017489, NM_018941, NM_001102, NM_015913, NM_000878	1.53	5.54E-05
**regulation of cell cycle**	3.51	NM_052988, NM_001018160, NM_001259, NM_181839, NM_018454, NM_006837, NM_001923, NM_003183, NM_006342, NM_000075, NM_004964, NM_005197, NM_199246, NM_013376, NM_001005413, NM_017900, NM_002166, NM_153201, NM_003072, NM_014397, NM_002497, NM_079421, NM_032637, NM_001039802, NM_003292, NM_002048, NM_001786, NM_016446, NM_016343, NM_014303, NM_001760, NM_018223, NM_015356, NM_001007793, NM_004091, NM_078468, NM_001078645, NM_001527, NM_078469, NM_078626, NM_012448, NM_003504, NM_198971, NM_001896, NM_015675, NM_152896, NM_005334, NM_003858, NM_001018677, NM_002229, NM_002228, NM_000595, NM_000051, NM_000594, NM_002567, NM_015113, NM_014750, NM_058195, NM_015920, NM_003903, NM_002167, NM_016567, NM_007065, NM_181803, NM_001031716, NM_025180, NM_001039619, NM_001706, NM_016426, NM_002027, NM_004343, NM_206876, NM_017489, NM_004336, NM_016399, NM_012341, NM_015201, NM_016290	1.92	2.13E-05
**regulation of cell death**	6.83	NM_001018160, NM_000600, NM_015331, NM_000700, NM_016558, NM_004355, NM_015713, NM_002094, NM_002093, NM_198175, NM_004964, NM_021009, NM_001165, NM_005605, NM_004208, NM_001079864, NM_001540, NM_001024465, NM_001024466, NM_014489, NM_006472, NM_032637, NM_020529, NM_005347, NM_002412, NM_133328, NM_007350, NM_003333, NM_016553, NM_012448, NM_181696, NM_007315, NM_001025159, NM_019071, NM_021141, NM_001018677, NM_004331, NM_147173, NM_021127, NM_000595, NM_006793, NM_000051, NM_000594, NM_003655, NM_003327, NM_058195, NM_004840, NM_004708, NM_006986, NM_032983, NM_006290, NM_001092, NM_001605, NM_033657, NM_001706, NM_152240, NM_005658, NM_004343, NM_006708, NM_001154, NM_004994, NM_001033030, NM_001151, NM_005789, NM_016399, NM_001970, NM_145074, NM_001556, NM_001007277, NM_004435, NM_002634, NM_201559, NM_002157, NM_022767, NM_003183, NM_002156, NM_014762, NM_032494, NM_003404, NM_000945, NM_019555, NM_017852, NM_005507, NM_172014, NM_058246, NM_003824, NM_015367, NM_206955, NM_003583, NM_000024, NM_004309, NM_000123, NM_001001391, NM_006573, NM_002957, NM_153498, NM_016037, NM_022873, NM_004052, NM_079421, NM_020396, NM_145813, NM_033015, NM_003295, NM_001786, NM_002048, NM_003299, NM_003591, NM_003810, NM_182908, NM_000852, NM_001025243, NM_015356, NM_004394, NM_006427, NM_078626, NM_006378, NM_003690, NM_019887, NM_004417, NM_003897, NM_013247, NM_030755, NM_033007, NM_004379, NM_001731, NM_001014451, NM_005531, NM_153719, NM_002226, NM_002228, NM_004281, NM_003946, NM_013974, NM_003806, NM_003804, NM_015920, NM_003807, NM_002167, NM_001005332, NM_213566, NM_003768, NM_004401, NM_003217, NM_001189, NM_018441, NM_002027, NM_018445, NM_017489, NM_018941, NM_001102, NM_015913, NM_000878	1.52	5.72E-05
**regulation of programmed cell death**	6.79	NM_001018160, NM_000600, NM_015331, NM_000700, NM_016558, NM_004355, NM_015713, NM_002094, NM_002093, NM_198175, NM_004964, NM_021009, NM_001165, NM_005605, NM_004208, NM_001079864, NM_001540, NM_001024465, NM_001024466, NM_014489, NM_006472, NM_032637, NM_020529, NM_005347, NM_002412, NM_133328, NM_007350, NM_003333, NM_016553, NM_012448, NM_181696, NM_007315, NM_001025159, NM_019071, NM_021141, NM_001018677, NM_004331, NM_147173, NM_021127, NM_000595, NM_006793, NM_000051, NM_000594, NM_003655, NM_003327, NM_058195, NM_004840, NM_004708, NM_006986, NM_032983, NM_006290, NM_001092, NM_001605, NM_033657, NM_001706, NM_152240, NM_005658, NM_004343, NM_006708, NM_001154, NM_004994, NM_001033030, NM_005789, NM_016399, NM_001970, NM_145074, NM_001556, NM_001007277, NM_004435, NM_002634, NM_201559, NM_002157, NM_022767, NM_003183, NM_002156, NM_014762, NM_032494, NM_003404, NM_000945, NM_017852, NM_019555, NM_005507, NM_172014, NM_058246, NM_003824, NM_015367, NM_206955, NM_003583, NM_000024, NM_004309, NM_000123, NM_001001391, NM_006573, NM_002957, NM_153498, NM_016037, NM_022873, NM_004052, NM_079421, NM_020396, NM_145813, NM_033015, NM_003295, NM_001786, NM_002048, NM_003299, NM_003591, NM_003810, NM_182908, NM_000852, NM_001025243, NM_015356, NM_004394, NM_006427, NM_078626, NM_006378, NM_003690, NM_019887, NM_004417, NM_003897, NM_013247, NM_030755, NM_033007, NM_004379, NM_001731, NM_001014451, NM_005531, NM_153719, NM_002226, NM_002228, NM_004281, NM_003946, NM_013974, NM_003806, NM_003804, NM_015920, NM_003807, NM_002167, NM_001005332, NM_213566, NM_003768, NM_004401, NM_003217, NM_001189, NM_018441, NM_002027, NM_018445, NM_017489, NM_018941, NM_001102, NM_015913, NM_000878	1.51	6.72E-05
**response to DNA damage stimulus**	3.37	NM_002350, NM_001018160, NM_002947, NM_003146, NM_201559, NM_017518, NM_001923, NM_022767, NM_014454, NM_001033002, NM_014060, NM_015713, NM_198954, NM_001618, NM_002878, NM_002876, NM_003583, NM_005197, NM_005652, NM_000123, NM_003211, NM_004208, NM_001033858, NM_005485, NM_003348, NM_001024465, NM_001024466, NM_014489, NM_006027, NM_079421, NM_002911, NM_145813, NM_152221, NM_001786, NM_002916, NM_001003686, NM_000234, NM_172166, NM_014502, NM_002412, NM_007192, NM_078468, NM_078469, NM_175069, NM_001042697, NM_004219, NM_005395, NM_198971, NM_001990, NM_021141, NM_030811, NM_005531, NM_002528, NM_198948, NM_001127218, NM_014481, NM_000051, NM_001048172, NM_206839, NM_015920, NM_002441, NM_016567, NM_001031716, NM_025180, NM_182776, NM_152240, NM_001706, NM_016426, NM_005066, NM_206876, NM_006304, NM_004029, NM_020135, NM_018248, NM_015913, NM_016399, NM_016290, NM_004237	1.63	0.003303519
**RNA processing**	5.51	NM_173797, NM_006527, NM_019037, NM_017706, NM_017892, NM_018256, NM_006112, NM_016001, NM_001033714, NM_025265, NM_006938, NM_006196, NM_018648, NM_015721, NM_019083, NM_020661, NM_203456, NM_018077, NM_058219, NM_203457, NM_182691, NM_020158, NM_133328, NM_139243, NM_003142, NM_018072, NM_015004, NM_032993, NM_004719, NM_002137, NM_005837, NM_002138, NM_014884, NM_003096, NM_002897, NM_003095, NM_003092, NM_016207, NM_021128, NM_058195, NM_032905, NM_203288, NM_016222, NM_001358, NM_021974, NM_001605, NM_003729, NM_017646, NM_002685, NM_001039619, NM_018285, NM_006392, NM_005066, NM_031263, NM_005371, NM_003089, NM_017755, NM_023010, NM_004593, NM_198220, NM_012426, NM_018983, NM_001363, NM_015659, NM_017612, NM_012207, NM_020175, NM_001002019, NM_080598, NM_024102, NM_032864, NM_003683, NM_014672, NM_015056, NM_001077442, NM_016037, NM_080632, NM_032177, NM_001042581, NM_194247, NM_001436, NM_015360, NM_001042588, NM_014303, NM_017838, NM_014502, NM_031844, NM_004396, NM_004939, NM_006372, NM_003690, NM_006857, NM_025193, NM_007362, NM_004493, NM_001034194, NM_052965, NM_004559, NM_001325, NM_022366, NM_197958, NM_003946, NM_032881, NM_001033505, NM_012123, NM_007359, NM_006170, NM_021177, NM_006558, NM_006559, NM_004698, NM_031369, NM_021931, NM_021190, NM_001078166, NM_006743, NM_017853, NM_020236, NM_004814, NM_020230, NM_014829, NM_033416, NM_001017392, NM_001005335, NM_015201	1.82	2.00E-07
**apoptosis**	4.98	NM_001018160, NM_024900, NM_000600, NM_015331, NM_002094, NM_021009, NM_001165, NM_005605, NM_004862, NM_030974, NM_001079864, NM_004208, NM_001024465, NM_001024466, NM_198057, NM_020529, NM_004091, NM_133328, NM_007350, NM_003333, NM_016077, NM_004765, NM_001008540, NM_007315, NM_016640, NM_004331, NM_003467, NM_021127, NM_000595, NM_000051, NM_000594, NM_058195, NM_004840, NM_004708, NM_006986, NM_032983, NM_006290, NM_057178, NM_016222, NM_001092, NM_005165, NM_033657, NM_152240, NM_005658, NM_001040021, NM_013374, NM_001033030, NM_005789, NM_016399, NM_145074, NM_024755, NM_004435, NM_024622, NM_201559, NM_002157, NM_002156, NM_022767, NM_014762, NM_000945, NM_003404, NM_016498, NM_017852, NM_019555, NM_003824, NM_015367, NM_003583, NM_024948, NM_003680, NM_014397, NM_004052, NM_133171, NM_022873, NM_020396, NM_145813, NM_033015, NM_201430, NM_025126, NM_003810, NM_015356, NM_004394, NM_006427, NM_014452, NM_014800, NM_001375, NM_019887, NM_001035235, NM_015675, NM_016068, NM_004089, NM_003897, NM_013247, NM_003374, NM_033007, NM_001636, NM_002228, NM_004281, NM_018234, NM_003946, NM_003806, NM_003804, NM_004282, NM_199122, NM_001005332, NM_213566, NM_003768, NM_004401, NM_003217, NM_198282, NM_031892, NM_017489, NM_199139, NM_015913	1.50	0.002276344

cellular macromolecule localization	3.65	NM_001034025, NM_012461, NM_015049, NM_024295, NM_015161, NM_001008213, NM_032520, NM_024658, NM_031885, NM_004355, NM_033028, NM_003404, NM_001033002, NM_006114, NM_002093, NM_018144, NM_001042535, NM_020243, NM_005652, NM_007342, NM_005488, NM_005015, NM_007347, NM_003135, NM_001042581, NM_005829, NM_006826, NM_001039802, NM_003292, NM_006327, NM_001003786, NM_015171, NM_012215, NM_001042588, NM_020529, NM_003916, NM_003917, NM_000308, NM_000466, NM_004217, NM_014874, NM_003145, NM_015044, NM_001007067, NM_004085, NM_003715, NM_003164, NM_001025159, NM_001136232, NM_001001433, NM_002228, NM_015254, NM_000390, NM_000594, NM_198883, NM_012479, NM_003134, NM_020750, NM_000288, NM_001283, NM_015638, NM_007359, NM_012414, NM_032985, NM_144497, NM_032511, NM_057178, NM_007065, NM_175624, NM_004603, NM_005796, NM_001706, NM_004343, NM_006947, NM_018445, NM_000428, NM_006809, NM_006803, NM_001970	1.60	0.003289594
cellular protein localization	3.61	NM_001034025, NM_012461, NM_015049, NM_024295, NM_015161, NM_001008213, NM_032520, NM_024658, NM_031885, NM_004355, NM_033028, NM_003404, NM_001033002, NM_006114, NM_002093, NM_018144, NM_001042535, NM_020243, NM_005652, NM_007342, NM_005488, NM_005015, NM_007347, NM_003135, NM_001042581, NM_005829, NM_006826, NM_001039802, NM_003292, NM_006327, NM_001003786, NM_015171, NM_012215, NM_001042588, NM_020529, NM_003916, NM_003917, NM_000308, NM_000466, NM_004217, NM_014874, NM_003145, NM_015044, NM_001007067, NM_004085, NM_003715, NM_003164, NM_001025159, NM_001136232, NM_001001433, NM_002228, NM_015254, NM_000390, NM_000594, NM_198883, NM_012479, NM_003134, NM_020750, NM_000288, NM_001283, NM_015638, NM_012414, NM_032985, NM_144497, NM_032511, NM_057178, NM_007065, NM_175624, NM_004603, NM_005796, NM_001706, NM_004343, NM_006947, NM_018445, NM_000428, NM_006809, NM_006803, NM_001970	1.59	0.004208927
intracellular protein transport	3.23	NM_001034025, NM_015049, NM_024295, NM_015161, NM_001008213, NM_032520, NM_024658, NM_004355, NM_003404, NM_001033002, NM_006114, NM_002093, NM_018144, NM_001042535, NM_020243, NM_007342, NM_005488, NM_007347, NM_003135, NM_001042581, NM_005829, NM_006826, NM_006327, NM_003292, NM_001003786, NM_015171, NM_012215, NM_001042588, NM_020529, NM_003916, NM_003917, NM_000308, NM_000466, NM_014874, NM_003145, NM_015044, NM_001007067, NM_004085, NM_003715, NM_003164, NM_001025159, NM_001136232, NM_001001433, NM_002228, NM_015254, NM_000390, NM_000594, NM_198883, NM_012479, NM_003134, NM_020750, NM_000288, NM_001283, NM_015638, NM_012414, NM_032985, NM_144497, NM_032511, NM_057178, NM_007065, NM_004603, NM_005796, NM_001706, NM_004343, NM_006947, NM_018445, NM_000428, NM_006809, NM_006803, NM_001970	1.56	0.014887636
negative regulation of apoptosis	3.13	NM_000600, NM_000700, NM_002156, NM_003183, NM_014762, NM_004355, NM_005507, NM_002093, NM_198175, NM_206955, NM_004964, NM_021009, NM_004309, NM_001165, NM_000123, NM_001079864, NM_001540, NM_001024465, NM_006573, NM_001024466, NM_014489, NM_004052, NM_022873, NM_079421, NM_032637, NM_020396, NM_001786, NM_003295, NM_005347, NM_020529, NM_003299, NM_182908, NM_001025243, NM_000852, NM_016553, NM_003333, NM_012448, NM_006378, NM_003897, NM_001025159, NM_021141, NM_030755, NM_001018677, NM_004331, NM_153719, NM_004281, NM_000594, NM_000051, NM_006793, NM_003946, NM_013974, NM_003655, NM_003806, NM_032983, NM_006290, NM_003768, NM_213566, NM_003217, NM_004401, NM_001605, NM_001189, NM_001706, NM_002027, NM_018445, NM_006708, NM_001154, NM_001033030, NM_018941, NM_016399, NM_000878, NM_001556	1.60	0.009780471
negative regulation of cell death	3.18	NM_000600, NM_000700, NM_002156, NM_003183, NM_014762, NM_004355, NM_005507, NM_002093, NM_198175, NM_206955, NM_004964, NM_021009, NM_004309, NM_001165, NM_000123, NM_001079864, NM_001540, NM_001024465, NM_006573, NM_001024466, NM_014489, NM_004052, NM_022873, NM_079421, NM_032637, NM_020396, NM_001786, NM_003295, NM_005347, NM_020529, NM_003299, NM_182908, NM_001025243, NM_000852, NM_016553, NM_003333, NM_012448, NM_006378, NM_003897, NM_001025159, NM_021141, NM_030755, NM_001018677, NM_004331, NM_153719, NM_004281, NM_000594, NM_000051, NM_006793, NM_003946, NM_013974, NM_003655, NM_003806, NM_032983, NM_006290, NM_003768, NM_213566, NM_003217, NM_004401, NM_001605, NM_001189, NM_001706, NM_002027, NM_018445, NM_006708, NM_001154, NM_001033030, NM_001151, NM_018941, NM_016399, NM_000878, NM_001556	1.60	0.009381883
negative regulation of programmed cell death	3.13	NM_000600, NM_000700, NM_002156, NM_003183, NM_014762, NM_004355, NM_005507, NM_002093, NM_198175, NM_206955, NM_004964, NM_021009, NM_004309, NM_001165, NM_000123, NM_001079864, NM_001540, NM_001024465, NM_006573, NM_001024466, NM_014489, NM_004052, NM_022873, NM_079421, NM_032637, NM_020396, NM_001786, NM_003295, NM_005347, NM_020529, NM_003299, NM_182908, NM_001025243, NM_000852, NM_016553, NM_003333, NM_012448, NM_006378, NM_003897, NM_001025159, NM_021141, NM_030755, NM_001018677, NM_004331, NM_153719, NM_004281, NM_000594, NM_000051, NM_006793, NM_003946, NM_013974, NM_003655, NM_003806, NM_032983, NM_006290, NM_003768, NM_213566, NM_003217, NM_004401, NM_001605, NM_001189, NM_001706, NM_002027, NM_018445, NM_006708, NM_001154, NM_001033030, NM_018941, NM_016399, NM_000878, NM_001556	1.58	0.013952046
translation	4.13	NM_015659, NM_002948, NM_006837, NM_001014438, NM_213725, NM_002949, NM_152295, NM_016497, NM_014763, NM_032025, NM_002094, NM_015904, NM_001013, NM_001961, NM_021009, NM_032111, NM_001967, NM_002161, NM_000983, NM_001969, NM_033362, NM_000985, NM_003680, NM_001013703, NM_033363, NM_013406, NM_013302, NM_002109, NM_018997, NM_001024921, NM_003292, NM_017840, NM_033301, NM_001040436, NM_001029, NM_024996, NM_198244, NM_146387, NM_031903, NM_003750, NM_001024, NM_003333, NM_000991, NM_007208, NM_022734, NM_016077, NM_000995, NM_014180, NM_033251, NM_016067, NM_022061, NM_001037283, NM_003751, NM_020320, NM_016640, NM_024580, NM_032479, NM_017827, NM_170738, NM_172177, NM_138395, NM_015969, NM_024077, NM_006360, NM_004461, NM_182917, NM_021121, NM_176792, NM_003907, NM_015920, NM_001412, NM_003321, NM_014078, NM_001605, NM_145644, NM_023937, NM_006295, NM_014175, NM_019041, NM_016050, NM_004446, NM_001402, NM_006743, NM_020236, NM_016093, NM_002887, NM_000976, NM_016622, NM_199177, NM_025150, NM_001970, NM_012423, NM_015956	2.26	9.55E-10

Supplementary Table S2. Filtered biological processes in HL cells 24 hours after AEZS-136 treatment. DAVID Bioinformatics Resources were used to identify the enriched biological processes for the genes modulated in the L-540 and SUP-HD1 cells or the KM-H2 and L-428 cells at 24 hours. The data show biological processes filtered for fold-enrichment >1.2, adjusted $P < 0.05$ and percentage of genes > 3.

Supplementary Table S3.

		log2FC			
**RefSeq mRNA**	**GENE**	**L-540**	**SUP-HD1**	**L-428**	**KM-H2**
NM_145813	**AIFM1**	-0.48	-0.50	-0.51	0.00
NM_019887	**DIABLO**	0.67	0.00	0.57	0.69
NM_133171	**ELMO2**	0.69	0.45	0.45	0.00
NM_201559	**FOXO3**	0.49	0.98	0.60	0.00
NM_002157	**HSPE1**	-1.08	-1.16	-0.77	0.00
NM_000595	**LTA**	-1.40	0.68	0.00	0.65
NM_016640	**MRPS30**	-0.46	-0.45	-0.47	0.00
NM_024900	**PHF17**	-0.73	0.00	-0.67	-0.44
NM_005789	**PSME3**	-0.66	0.00	-0.77	-0.41
NM_016077	**PTRH2**	-0.68	-0.44	-0.50	0.00
NM_003804	**RIPK1**	0.74	0.40	0.91	0.00
NM_201430	**RTN3**	0.52	0.47	0.86	0.00
NM_006427	**SIVA1**	-1.03	-0.46	0.00	-0.42
NM_024755	**SLTM**	0.49	0.56	0.49	0.00
NM_005658	**TRAF1**	0.56	0.78	0.50	0.00
NM_198057	**TSC22D3**	0.76	1.63	1.99	0.00
NM_004089	**TSC22D3**	0.43	1.34	1.31	0.00
NM_152240	**ZMAT3**	0.51	0.77	0.46	0.00
NM_002156	**HSPD1**	-0.72	-0.66	-0.70	0.00
NM_004355	**CD74**	-0.48	0.00	0.44	-0.52
NM_198175	**NME1**	-0.89	-0.58	-0.52	0.00
NM_001540	**HSPB1**	-0.47	0.00	-0.86	-0.56
NM_001024465	**SOD2**	-0.60	-0.69	-0.59	0.00
NM_032637	**SKP2**	-0.53	-0.50	0.00	-0.47
NM_001786	**CDK1**	-1.01	-0.88	0.00	-0.41
NM_003295	**TPT1**	0.70	0.44	0.65	0.00
NM_016553	**NUP62**	0.00	-0.72	-0.49	-0.66
NM_006378	**SEMA4D**	-0.91	0.00	-0.44	-0.45
NM_003897	**IER3**	-0.90	0.00	0.60	0.54
NM_001025159	**CD74**	0.00	0.49	0.87	-0.63
NM_001018677	**FNTA**	0.63	0.00	0.77	0.64
NM_000051	**ATM**	0.84	0.44	0.00	0.58
NM_003946	**NOL3**	0.56	0.00	0.48	0.62
NM_006290	**TNFAIP3**	0.45	0.78	0.42	0.00
NM_003768	**PEA15**	0.91	0.57	0.00	0.85
NM_003217	**TMBIM6**	0.91	0.93	0.73	0.00
NM_001605	**AARS**	0.77	0.65	0.00	0.90

NM_018445	**VIMP**	0.53	0.00	0.47	0.58
NM_006708	**GLO1**	-0.85	-0.60	-0.44	0.00
NM_018941	**CLN8**	1.00	0.00	0.54	0.58
NM_001092	**ABR**	0.69	0.00	-0.67	0.00
NM_019555	**ARHGEF3**	0.00	0.00	-0.58	-0.54
NM_015367	**BCL2L13**	1.07	0.00	0.00	0.61
NM_001008540	**CXCR4**	-0.43	0.00	0.00	-0.63
NM_003467	**CXCR4**	-0.44	0.00	0.00	-0.68
NM_033657	**DAP3**	0.00	0.00	0.41	0.53
NM_133328	**DEDD2**	0.74	0.00	0.00	0.43
NM_001375	**DNASE2**	1.08	0.00	0.61	0.00
NM_024948	**FAM188A**	0.49	0.00	0.00	0.70
NM_015675	**GADD45B**	0.00	0.00	0.71	0.97
NM_002094	**GSPT1**	0.00	-0.43	-0.63	0.00
NM_013247	**HTRA2**	0.00	0.00	0.56	0.44
NM_145074	**HTRA2**	0.00	0.00	0.69	0.42
NM_015331	**NCSTN**	0.00	0.46	0.00	0.41
NM_014397	**NEK6**	0.00	0.00	-1.49	-0.66
NM_004708	**PDCD5**	-0.73	0.00	-0.62	0.00
NM_013374	**PDCD6IP**	0.00	0.48	0.73	0.00
NM_021127	**PMAIP1**	0.62	0.00	0.00	0.45
NM_005605	**PPP3CC**	0.51	0.00	0.00	0.56
NM_007315	**STAT1**	-0.86	0.00	0.46	0.00
NM_199122	**TBRG4**	0.00	-0.55	-0.43	0.00
NM_198282	**TMEM173**	0.74	0.00	0.00	-0.58
NM_003810	**TNFSF10**	1.02	0.00	0.87	0.00
NM_003374	**VDAC1**	-0.41	0.00	-0.43	0.00
NM_003680	**YARS**	0.65	0.00	0.00	1.05
NM_000600	**IL6**	0.00	0.78	0.00	0.47
NM_003183	**ADAM17**	0.68	0.00	0.42	0.00
NM_014762	**DHCR24**	0.00	-0.64	-0.58	0.00
NM_002093	**GSK3B**	0.00	-0.54	-0.51	0.00
NM_206955	**PRAME**	0.00	0.00	-0.53	-0.61
NM_004964	**HDAC1**	-0.58	0.00	-0.45	0.00
NM_004309	**ARHGDIA**	0.00	-0.47	0.00	-0.48
NM_000123	**ERCC5**	0.40	0.00	0.43	0.00
NM_006573	**TNFSF13B**	-0.50	0.00	0.00	-0.80
NM_079421	**CDKN2D**	0.42	0.00	0.58	0.00
NM_020529	**NFKBIA**	0.00	0.00	1.38	1.47
NM_003299	**HSP90B1**	-0.87	0.00	-0.80	0.00

NM_182908	**DHRS2**	0.00	0.00	1.06	0.47
NM_000852	**GSTP1**	-0.44	0.00	-0.54	0.00
NM_021141	**XRCC5**	-0.51	0.00	-0.57	0.00
NM_030755	**TMX1**	0.00	-0.57	-0.47	0.00
NM_004331	**BNIP3L**	0.00	0.00	0.45	-0.44
NM_153719	**NUP62**	0.00	-0.58	0.00	-0.58
NM_000594	**TNF**	-0.55	0.00	0.00	0.76
NM_006793	**PRDX3**	-0.58	0.00	-0.54	0.00
NM_032983	**CASP2**	0.00	-0.42	0.41	0.00
NM_001189	**NKX3-2**	0.00	0.50	0.57	0.00
NM_002027	**FNTA**	0.00	0.00	0.49	0.41
NM_001033030	**FAIM**	-0.46	0.00	0.00	-0.40
NM_016399	**TRIAP1**	0.00	0.00	-0.49	-0.54
NM_001151	**SLC25A4**	-0.48	0.00	-0.44	0.00
NM_022767	**AEN**	0.00	0.00	-0.75	0.00
NM_004208	**AIFM1**	0.00	0.00	-0.57	0.00
NM_005165	**ALDOC**	0.00	0.00	0.00	-0.42
NM_004840	**ARHGEF6**	0.00	0.00	0.00	-0.53
NM_004282	**BAG2**	0.00	0.00	-0.43	0.00
NM_004765	**BCL7C**	0.00	0.00	0.00	-0.60
NM_001040021	**CD14**	0.00	0.00	0.00	-0.68
NM_058195	**CDKN2A**	0.00	0.00	0.53	0.00
NM_004394	**DAP**	0.00	0.00	0.60	0.00
NM_016222	**DDX41**	0.00	0.00	0.42	0.00
NM_003583	**DYRK2**	0.00	0.00	-0.49	0.00
NM_004091	**E2F2**	0.00	0.00	-0.48	0.00
NM_014800	**ELMO1**	0.00	0.00	0.00	-0.44
NM_004435	**ENDOG**	0.00	0.00	-0.49	0.00
NM_003824	**FADD**	0.00	0.00	-0.41	0.00
NM_033015	**FASTK**	0.00	0.00	-0.42	0.00
NM_024622	**FASTKD1**	0.00	0.00	-0.45	0.00
NM_016068	**FIS1**	0.00	0.00	0.00	-0.51
NM_002228	**JUN**	0.00	0.00	0.74	0.00
NM_004862	**LITAF**	0.00	0.00	0.42	0.00
NM_006986	**MAGED1**	0.00	0.00	0.00	-0.40
NM_001005332	**MAGED1**	0.00	0.00	0.00	-0.49
NM_016498	**MTFP1**	0.00	0.00	-0.51	0.00
NM_001018160	**NAE1**	0.00	0.00	-0.59	0.00
NM_033007	**NLRP1**	0.00	0.00	0.48	0.00
NM_017852	**NLRP2**	0.00	0.00	0.00	-0.42

NM_007350	**PHLDA1**	0.00	0.00	0.41	0.00
NM_000945	**PPP3R1**	0.00	0.00	-0.59	0.00
NM_057178	**RFFL**	0.00	0.00	0.87	0.00
NM_025126	**RNF34**	0.00	0.00	-0.45	0.00
NM_015356	**SCRIB**	0.00	0.00	-0.48	0.00
NM_031892	**SH3KBP1**	0.00	0.00	0.00	-0.95
NM_030974	**SHARPIN**	0.00	0.00	0.56	0.00
NM_001636	**SLC25A6**	0.00	0.00	0.00	0.43
NM_001035235	**SRA1**	0.00	0.00	-0.49	0.00
NM_018234	**STEAP3**	0.00	0.00	-0.99	0.00
NM_017489	**TERF1**	0.00	0.00	0.43	0.00
NM_014452	**TNFRSF21**	0.00	0.00	-0.51	0.00
NM_015913	**TXNDC12**	0.00	0.00	0.77	0.00
NM_199139	**XAF1**	0.00	0.00	1.04	0.00
NM_003404	**YWHAB**	0.00	0.00	0.00	-0.41
NM_000700	**ANXA1**	0.00	0.00	-0.41	0.00
NM_005507	**CFL1**	0.00	0.00	0.00	-0.42
NM_021009	**UBC**	0.00	0.00	0.44	0.00
NM_001165	**BIRC3**	0.00	0.00	-0.56	0.00
NM_001079864	**TAX1BP1**	0.00	0.00	0.43	0.00
NM_001024466	**SOD2**	0.00	0.00	-0.53	0.00
NM_014489	**PGAP2**	0.00	0.00	0.00	0.46
NM_004052	**BNIP3**	0.00	0.00	-0.53	0.00
NM_022873	**IFI6**	0.00	0.00	0.00	-0.83
NM_020396	**BCL2L10**	0.00	0.00	-0.74	0.00
NM_005347	**HSPA5**	0.00	0.00	-0.43	0.00
NM_001025243	**IRAK1**	0.00	0.00	0.00	0.42
NM_003333	**UBA52**	0.00	0.00	0.00	0.74
NM_012448	**STAT5B**	0.00	0.00	0.51	0.00
NM_004281	**BAG3**	0.00	0.00	-0.51	0.00
NM_013974	**DDAH2**	0.00	0.00	0.00	0.41
NM_003655	**CBX4**	0.00	0.00	0.42	0.00
NM_003806	**HRK**	0.00	0.00	0.00	0.76
NM_213566	**DFFA**	0.00	0.00	0.00	0.50
NM_004401	**DFFA**	0.00	0.00	0.00	0.61
NM_001706	**BCL6**	0.00	0.00	0.00	-0.67
NM_001154	**ANXA5**	0.00	0.00	0.94	0.00
NM_000878	**IL2RB**	0.00	0.00	0.00	-0.41
NM_001556	**IKBKB**	0.00	0.00	0.53	0.00

Supplementary Table S3. HL-modulated genes in KM-H2- and L-428-specific processes. The table shows the modulated genes involved in specific biological processes in the KM-H2 and L-428 HL cell lines (Fig. 3a, blue nodes) at 24 hours. The values are presented as the log_2 fold-changes. Red= upregulated genes; Green= dowreguleted genes; Grey= not significative modulated genes.
